# Genome-Wide Analysis of mRNA and Long Noncoding RNA Profiles in Chronic Actinic Dermatitis

**DOI:** 10.1155/2017/7479523

**Published:** 2017-11-14

**Authors:** Dongyun Lei, Lechun Lv, Li Yang, Wenjuan Wu, Yong Liu, Ying Tu, Dan Xu, Yumei Jin, Xiang Nong, Li He

**Affiliations:** ^1^Department of Dermatology, First Affiliated Hospital of Kunming Medical University, Kunming, Yunnan, China; ^2^Yan'an Affiliated Hospital of Kunming Medical University, Kunming, Yunnan, China

## Abstract

Chronic actinic dermatitis (CAD), a photosensitive dermatosis, is characterized by inflammatory lesions, especially on sun-exposed skin. However, its pathogenesis remains unclear. In this study, second-generation RNA sequencing and comprehensive bioinformatics analyses of mRNAs and long noncoding RNAs (lncRNAs) were performed to determine the transcriptome profiles of patients with CAD. A total 6889 annotated lncRNAs, 341 novel lncRNAs, and 65091 mRNAs were identified. Interestingly, patients with CAD and healthy controls showed distinct transcriptome profiles. Indeed, 198 annotated (81.48%) and 45 novel (18.52%) lncRNAs were differentially expressed between the two groups. GO, KEGG, and RGSEA analyses of lncRNAs showed that inflammatory and immune response related pathways played crucial roles in the pathogenetic mechanism of CAD. In addition, we unveiled key differentially expressed lncRNAs, including lncRNA RP11-356I2.4 which plays a role probably by regulating TNFAIP3 and inflammation. qRT-PCR data validated the differentially expressed genes. The newly identified lncRNAs may have potential roles in the development of CAD; these findings lay a solid foundation for subsequent functional exploration of lncRNAs and mRNAs as therapeutic targets for CAD.

## 1. Introduction

Chronic actinic dermatitis (CAD) is an immunologically mediated photosensitive disorder, which shows pruritic eczematous lesions, mainly on sun-exposed skin [1]. The morbidity rates of CAD in Europe, United States, and Asia overtly increase in hot and tropical regions or in summer [2–4]. Severe cases can evolve into cutaneous T-cell lymphoma, which is considered a malignant neoplasm [5]. Moreover, noncosmetic appearance and conscious itching induced by CAD affect the physical and psychological health of patients severely [6]. Previously, immunologic dysregulations, such as T-cell-mediated delayed-type hypersensitivity and loss of immunosuppression to UV-induced neoantigen, were proposed to play a critical role in CAD [7, 8]; however, the underlying pathogenic mechanisms of CAD remain incompletely elucidated and need further investigation.

The rapid evolution of whole transcriptome profiling using next-generation high-throughput RNA sequencing (RNA-Seq) has shed light on the complex mechanisms and pathways of multiple dermatoses, including squamous cell carcinoma (SCC) and basal cell carcinoma (BCC) [9–11]. However, few studies have assessed the differential gene expression profiles of skin samples from patients with CAD or explored mRNA and long noncoding RNA (lncRNA) profiles for their associations with CAD pathogenesis. LncRNAs have long been mistaken for “transcription junk” or “dark matter” without biological functions, but defining their functions has become an increasingly interesting field [12–14]. LncRNAs with multiexon and longer than 200 nucleotides belong to noncoding RNAs, which do not code for proteins and are less abundant than mRNAs [15]. LncRNAs are ubiquitous in nature, but highly specific to tissues or cells. LncRNA functions have gained much attention, and these molecules play important roles in physiological and pathological processes by regulating transcriptional activity and chromatin modification [14, 16]. Indeed, the roles of lncRNAs in regulating inflammatory disorders, such as psoriasis, asthma, and immunologic signaling pathways, have recently been demonstrated [17–19]. However, whether lncRNAs participate in the pathogenesis of CAD remains largely unknown. Therefore, it is urgent to identify CAD-related lncRNAs and establish a firm foundation for further functional analyses, which may elucidate the pathogenesis of CAD.

In this study, lncRNA and mRNA profiles of skin tissues from the exposed sites of patients with CAD and normal subjects were systematically identified and comprehensively analyzed using RNA-Seq, and both annotated and novel transcripts were detected. This study aimed to reveal discrepancies in lncRNA and mRNA expression levels and to identify critical pathways and pivotal pathogenic genes which may be important in the pathogenesis of CAD.

## 2. Materials and Methods

### 2.1. Sample Collection

Study participants were all Chinese recruited at the Department of Dermatology of First Affiliated Hospital of Kunming Medical University. The enrolled participants were assigned into patients with CAD (*n* = 4) and controls (*n* = 4) for RNA-Seq. To further evaluate RNA-Seq findings, 4 additional patients with CAD and 4 normal controls were included for quantitative RT-PCR (qRT-PCR) verification. The study was approved by the Medical Ethics Committee of Kunming Medical University (number 2016-33); informed written consent was provided by each participant. The diagnosis was based on clinical and photobiological evaluation by a photodermatologist. Details of subjects' characteristics are shown in Supplementary Table S1 in Supplementary Material available online at https://doi.org/10.1155/2017/7479523. There is no statistical difference in gender and age distributions in patients with CAD and controls, respectively. What is more, analysis revealed that results were not biased by gender and age (*p* > 0.05, Supplementary Table S1). None of the patients had received corticosteroids or immunosuppressants for at least 6 weeks preceding the study. In addition, patients with underlying chronic diseases, such as hypertension, diabetes, and dermatitis (including eczema and lupus erythematosus), were excluded.

**Figure 1:**
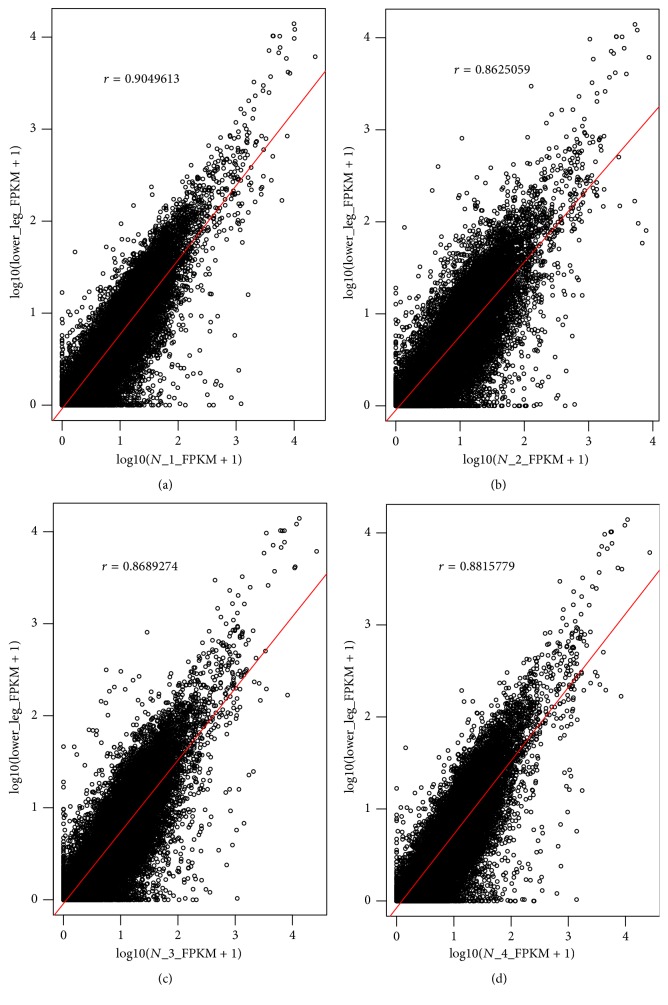
Comparisons of 4 skin samples of controls with normal skin samples of the public (sun-exposed skin) in GTEx study.

CAD tissues were collected during the pathologic examination of skin lesions. Tissue samples from patients with CAD were collected from infiltrated plaques in opisthotonic sites located at sun-exposed areas. Samples of controls were obtained from the same sun-exposed regions to avoid unnecessary discrepancy, and all the normal samples for RNA-seq are all qualified (*r* > 0.80) through testing with GTEx (genotype tissue expression project) study (Figure 1). IDs (Identities) of GTEx were represented in Supplementary Table S2. The collected tissue samples were immediately soaked in RNA preservation solution (QIAGEN, Germany) and stored at −80°C.

### 2.2. RNA Isolation and Assessment

Total RNA was isolated using the miRNeasy Mini Kit (QIAGEN, Germany) according to the manufacturer's instructions. RNA contamination and degradation were assessed by 1% agarose gel electrophoresis (AGE). RNA purity was detected by determining the OD260/280 ratio on a NanoPhotometer® spectrophotometer (IMPLEN, CA, USA). RNA integrity and concentration were quantified with RNA Nano 6000 Assay Kit on a Bioanalyzer 2100 system (Agilent Technologies, CA, USA) and Qubit® RNA Assay Kit in Qubit® 2.0 Flurometer (Life Technologies, CA, USA), respectively.

### 2.3. Genomic Library Construction and Sequencing

A total of 3 *μ*g RNA was used as input material for genomic library construction. First, ribosomal RNA (rRNA) was removed with Epicentre Ribo-zero™ rRNA Removal Kit (Epicentre, USA). Afterwards, sequencing libraries were generated with the dUTP method using rRNA-free RNA with NEBNext® Ultra™ Directional RNA Library Prep Kit for Illumina® (NEB, USA), according to the manufacturer's instructions. Then, RT-PCR was performed with Phusion High-Fidelity DNA polymerase, Index (*X*) Primer, and Universal PCR primers. Finally, the products were purified using the AMPure XP system, and library quality was evaluated on an Agilent Bioanalyzer 2100 system. The RNA library was sequenced on an Illumina Hiseq 4000 platform, and 150 bp paired-end reads were generated.

### 2.4. Data Analysis

Sequenced reads (raw data or raw reads) in the FASTQ format were first processed using in-house Perl scripts. In this step, clean reads were prepared by deleting reads containing ploy-N, adapters and low quality reads from raw data. Meanwhile, GC content, Q20, and Q30 of the cleaned data were also determined. All downstream analyses were based upon clean data, with high quality. Clean reads were mapped to the reference genome built with Bowtie (v2.0.6) [20]. Paired-end clean reads were aligned to the reference genome using TopHat (v2.0.9), and the parameter was set as ‘--library-type fr-firststrand'. The mapped reads were assembled by Cufflinks (v2.1.1) [21] in a reference-based approach. Cufflinks was run with ‘--library-type' and ‘min-frags-per-transfrag=0', and others were set as default parameters.

#### 2.4.1. Coding Potential and Conservation Analyses

Coding Potential Calculator (CPC), Coding Non-Coding Index (CNCI), phylogenetic codon substitution frequency (PhyloCSF), and fragments per kilo-base of exon per million fragments mapped (PFAM) were used to distinguish mRNAs from lncRNAs. CNCI profiles with default parameters were used in this study, effectively distinguishing noncoding and protein-coding sequences by adjoining nucleotide triplets [22]. By assessing the quality and extent of the ORF in a transcript, CPC searches sequences in the known protein sequence database to identify noncoding and coding transcripts [23]. PhyloCSF can distinguish noncoding and protein-coding transcripts by examining characteristic evolutionary signatures in alignment with conserved coding regions [24]. The transcripts predicted with coding potential by one or all four tools above were filtered out, with those without coding potential considered candidate lncRNAs.

Phylogenetic models for conserved and nonconserved regions among species were computed using phyloFit; then, model and HMM transition parameters were sent to PhastCons to compute a set of conservation scores of the coding genes and lncRNAs. Phast (v1.3) is a software package which contains multiple statistical programs and mostly used in phylogenetic analyses [25]. PhastCons is a conservation scoring and identification program for conserved elements.

#### 2.4.2. Differential Expression Analysis and Target Gene Prediction

Differences in gene or digital transcript expression were determined by Cuffdiff statistical routines [21]. Genes or transcripts with *p* < 0.05 were considered to be differentially expressed.

Target gene prediction of lncRNAs was performed as previously described [26]. Coding genes 100k upstream and downstream of lncRNAs were searched as the target genes in cis. Expression associations of coding genes with lncRNAs in different chromosomes were determined by Pearson correlation coefficient (PCC); with PCC > 0.95 or <−0.95, the lncRNA-mRNA pair was considered to represent target genes in trans.

#### 2.4.3. GO and KEGG Enrichment Analyses and RGSEA Analyses

Differentially expressed genes or transcripts were submitted to Gene Ontology (GO) enrichment and Kyoto Encyclopedia of Genes and Genomes (KEGG) pathway analyses, to assess overrepresented functional terms in the genomic background. GO enrichment was performed with the GO Seq R package, with GO terms showing *p* < 0.05 regarded as significantly enriched. KEGG is a database resource providing insights into high-level functions and utilities of the biological system (http://www.genome.jp/kegg/). The KOBAS software was used to assess statistical enrichment in KEGG pathways. Random Gene Set Enrichment Analysis (RGSEA) was also used to assess pathways enrichment using random set of lncRNA in the genome.

#### 2.4.4. PPI Analysis

PPI analysis of differentially expressed genes was based on the STRING database (http://string-db.org/) to further identify the functional roles of lncRNAs in regulating mRNAs. PPI analysis was carried out with the Cytoscape software (v7.0).

### 2.5. Quantitative Real Time-Polymerase Chain Reaction (qRT-PCR)

RNA isolation was performed as described above. Total cDNA was synthesized with RT^2^PreAMP cDNA Synthesis Kit (QIAGEN, Germany). All qRT-PCR primers (Supplementary Table S3) were confirmed to produce specific PCR products. RT-PCR reactions were performed on a ROTOR-GENE Q RT-PCR Facility (QIAGEN, Germany). The SYBR Green amplification system (20 *μ*l) included 10 *μ*l mix, 1 *μ*l of each primer, 1 *μ*l cDNA, and 7 *μ*l H_2_O. Cycling conditions included initial denaturation at 95°C for 10 minutes, followed by 40 cycles at 95°C for 10 s, 56°C for 20 s, and 72°C for 30 s. *β*-Actin was used as an internal control. Relative expression of target genes was evaluated by the 2^−ΔΔCt^ method. Dissociation curves were analyzed after amplification for all products. Independent replicates were set up for each target gene.

Statistical differences in gene expression were analyzed by determining *p* values based on Student's *t*-test for each gene in the controls and patients with CAD. *p* < 0.05 was considered statistically significant.

## 3. Results

### 3.1. Overview of RNA Sequencing, and mRNA and lncRNA Identification

Strand specific RNA-Seq of the whole transcriptome was applied to assess rRNA-depleted RNAs from 4 patients with CAD and 4 normal individuals to comprehensively identify lncRNAs and mRNAs related to CAD. A total of 776 million clean reads were obtained from 810 million raw reads. 716 million (92.27%) of them were mapped to the human genome (hg19), with 32 million reads (4.12%) aligned to multilocations; 603 million (77.70%) reads were aligned to unique-locations in the reference genome (Supplementary Table S4).

**Figure 2:**
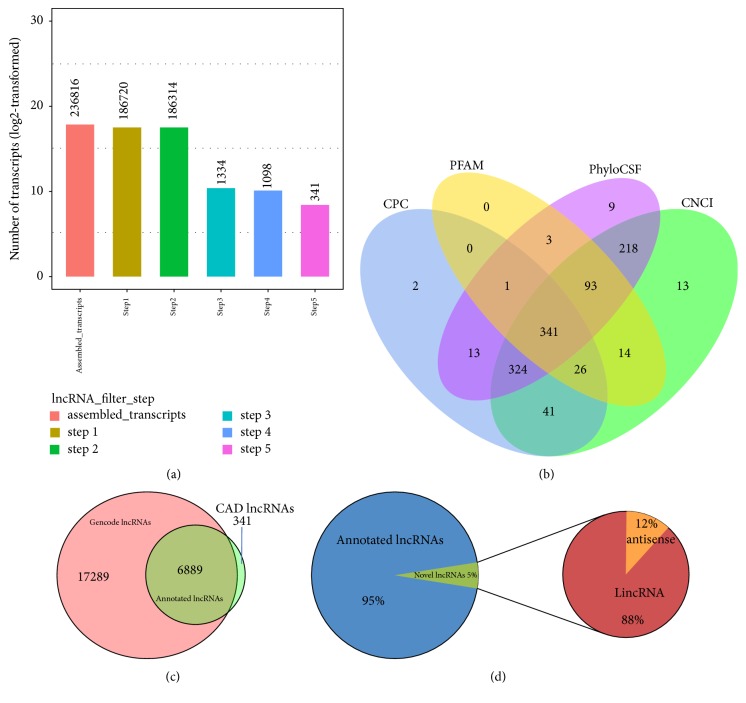
Computational pipeline for the systematic filtration of CAD lncRNAs and mRNAs. (a) Bar chart of lncRNAs screening outcome in each step of the computational pipeline for the systematic identification of CAD lncRNAs and mRNAs. Step 1, transcripts with exons ≥ 2 were retained; Step 2, transcripts > 200 bp in length were retained; Step 3, transcripts with >3 reads coverage and 0.5 FPKM were retained; Step 4, known non-lncRNA annotations were identified as annotated lncRNAs, and known classes of RNAs, including known protein-coding genes, microRNAs, tRNAs, miscRNA, rRNAs, and pseudogenes, were eliminated; Step 5, transcripts without coding potential detected using CPC, CNCI, PFAM, and PhyloCSF were considered novel lncRNAs. (b) Coding potency filter with 4 mainstream coding potential analysis methods, including CPC analysis, CNCI analysis, FFAM, and PhyloCSF. (c) A Venn diagram describing the overlap between our catalog of CAD lncRNAs and those of Gencode v19. (d) Pie chart describing the classification of identified lncRNAs.

A stringent filtering pipeline for transcripts of CAD was developed to identify lncRNAs from 236,816 assembled transcripts (Figures 2(a) and 2(b)). 7230 reliably expressed lncRNA isoforms derived from 5213 lncRNA loci and 65091 mRNA isoforms from 18179 mRNA loci were obtained via the filtering pipeline. 6889 (93.3%) were identified in Gencode v19 lncRNA annotation; the remaining 341 were novel lncRNAs, including 300 (87.98%) long intergenic ncRNAs (lincRNAs) and 41 (12.02%) antisense lncRNAs (Figures 2(c) and 2(d)).

### 3.2. Comparative Analysis of lncRNAs and mRNAs

**Figure 3:**
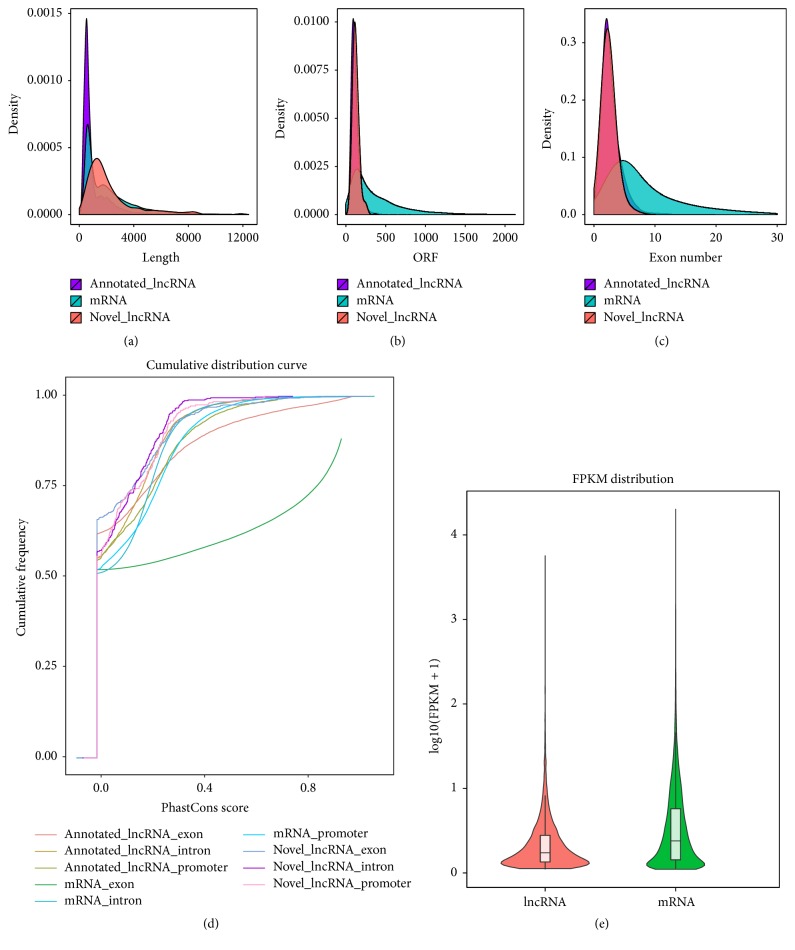
Comparative analyses of CAD lncRNAs and mRNAs. (a) Transcript size distribution of lncRNA and mRNA transcripts. (b) ORF lengths of lncRNA and mRNA transcripts. (c) Number of exons in lncRNA and mRNA transcripts. (d) Conservation levels of lncRNAs and mRNAs transcripts. (e) Violin plot of expression levels (shown in log_10_(FPKM + 1)) of lncRNA and mRNA transcripts.

The basic features of the lncRNAs were analyzed and compared with mRNAs. In agreement with previous studies [27], lncRNAs had shorter transcripts (Figure 3(a)), shorter ORFs (Figure 3(b)), fewer exons (Figure 3(c)), less conserved sequences (Figure 3(d)), and lower expression levels (Figure 3(e)). On overage, lncRNAs were 1168 bp in length and contained 2.84 exons. We also found that annotated and novel lncRNAs had similar exon numbers and ORF lengths, but different transcription lengths, and there were significant differences between putative lncRNAs and mRNAs. Putative lncRNAs had shorter ORFs and lower expression levels, in agreement with previous studies. Furthermore, in our dataset, predicted lncRNAs contained less exons than mRNAs. The characteristics of putative novel lncRNAs, including gene type, exon number, ORF length, and chromatin status, were summarized in Supplementary Table S5.

### 3.3. Differential Expression and Clustering Analyses

**Figure 4:**
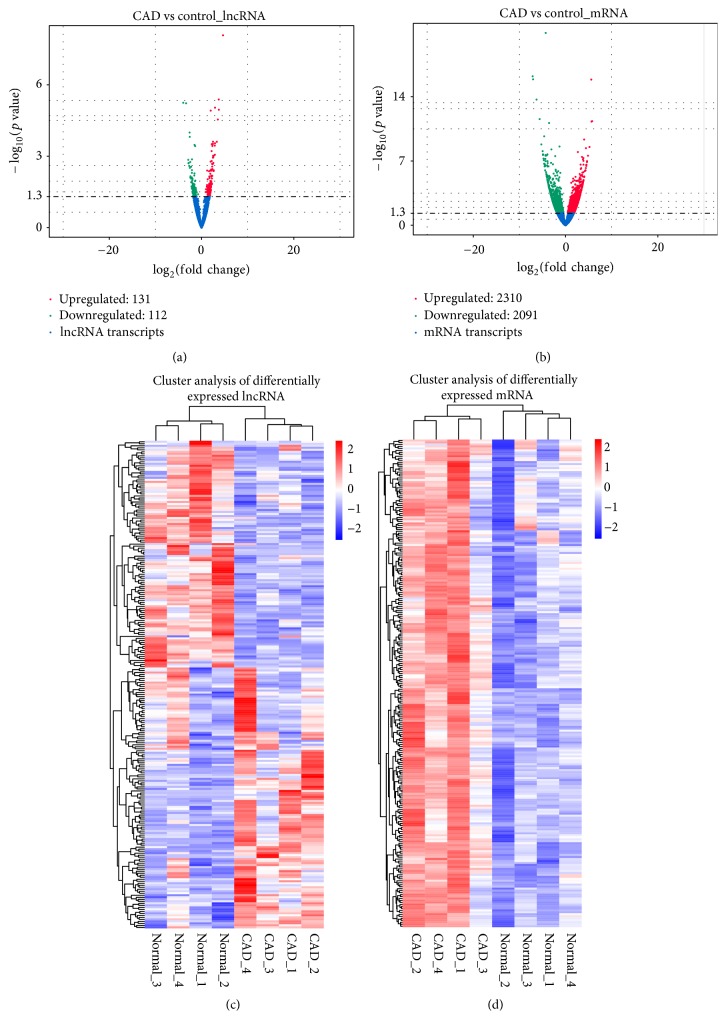
Differential expression of lncRNAs and mRNAs in patients with CAD and controls. Volcano plots of differentially expressed lncRNA (a) and mRNA (b) transcripts. Hierarchical clustering of the expression profiles of differentially expressed lncRNAs (c) and mRNAs (d).

Gene expression profiles in patients with CAD and controls were remarkably different. In patients with CAD, a total of 243 lncRNA transcripts (131 upregulated and 112 downregulated) and 4401 mRNA transcripts (2310 upregulated and 2091 downregulated) were differentially expressed compared with the controls (Figures 4(a) and 4(b)). Among the differentially expressed lncRNAs, 198 (81.48%) and 45 (18.52%) were annotated and novel, respectively. Differentially expressed lncRNAs and mRNAs were widely distributed in the genome, and found on almost all chromosomes. Hierarchical clustering analysis was performed for the differentially expressed lncRNAs and mRNAs, respectively. Heat maps showed overt self-segregated clusters in patients with CAD and controls (Figures 4(c) and 4(d)).

Among the most distinctively expressed mRNAs in patients with CAD in comparison with controls, 30 mRNAs had *p* value ≤ 5.93*E* − 08, with 11 upregulated and 19 downregulated (Table 1). The top 15 differentially expressed annotated (*p* < 0.014) and novel (*p* < 0.007) lncRNAs between the patients with CAD and controls are listed in Tables 2 and 3, respectively; 10 annotated and 14 novel lncRNAs were upregulated in patients with CAD, while 5 lncRNAs and 1 novel lncRNAs were downregulated. These data indicated remarkable differences between the lncRNA and mRNA expression profiles, suggesting potential roles for their activation or suppression in CAD development, or indirect association with CAD pathogenesis.

### 3.4. GO and KEGG Analyses Based on Differentially Expressed mRNAs

**Figure 5:**
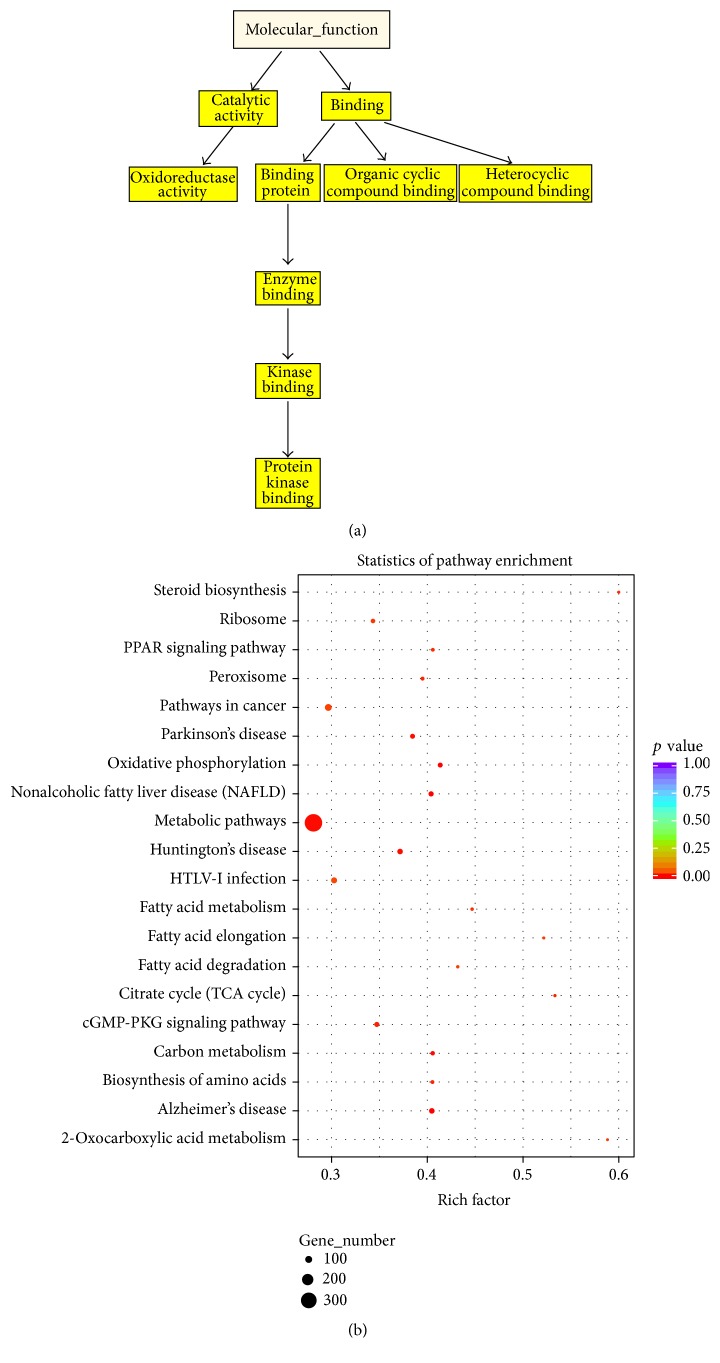
Dysregulated mRNAs of patients with CAD compared with controls. (a) Directed acyclic graph (DAG) of GO analysis based on common differentially expressed genes. GO terms marked in yellow are over-represented terms with statistical significance. (b) KEGG enrichment analysis of dysregulated mRNAs.

GO and KEGG pathway enrichment analyses were performed with differentially expressed mRNAs to confirm their functions. Significant overrepresented GO terms included oxidoreductase activity, protein binging, and protein kinase binding (Figure 5(a)). To infer systematic biological behaviors of the mRNAs, KEGG pathway analyses were conducted by mapping dysregulated mRNAs to KEGG reference pathways. Interestingly, metabolic pathways, peroxisome proliferator activated receptor (PPAR) signaling pathway, cancer pathway, human T lymphotropic virus type-I (HTLV-I) infection, and cyclic guanosine 3′,5′-monophosphate (cGMP-PKG) signaling pathway were significantly overrepresented (Figure 5(b), Supplementary Table S6).

Inflammatory and immune response related differentially expressed genes (DEGs) between patients with CAD and controls were prominent in the whole mRNA expression profile. Most DEGs had close associations with the immune response or inflammation. For example, among the top 10 differentially expressed mRNAs with *p* value ranging from 1.12*E* − 21 to 2.04*E* − 10, most mRNAs had immunological functions (Table 4). Additionally, skin barrier genes, such as CLDN5 and CLDN7, were dysregulated, indicating skin injury in CAD development.

### 3.5. Potential Target Prediction of lncRNAs and Functional Analyses

**Figure 6:**
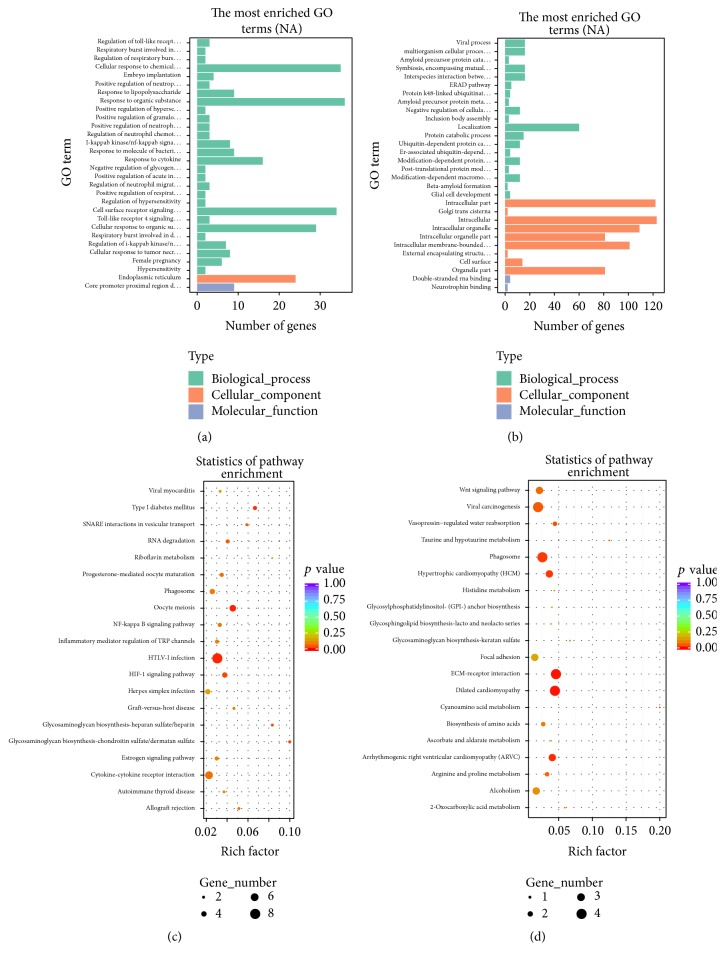
KEGG enrichment analyses of dysregulated lncRNAs. Analysis of the top 30 most enriched GO terms of lncRNAs with effects in cis (a) and trans (b) patterns. Analysis of the top 20 overrepresented KEGG pathways of lncRNAs with effects in cis (c) and trans (d) patterns. The enrichment factor was calculated as the number of enriched genes by that of all background genes in each pathway. Pathways with *p* value < 0.05 were identified as statistically significantly overrepresented.

To further predict the functions of lncRNAs differentially expressed between patients with CAD and controls, we performed GO analysis of cis- and trans-regulated target mRNAs, respectively. The most enriched GO terms in cis were related to immune response and inflammation, including “regulation of toll-like receptor 4 signaling pathway,” “respiratory burst involved in inflammatory response,” “regulation of respiratory burst involved in inflammatory response,” “cellular response to chemical stimulus embryo implantation,” “positive regulation of neutrophil chemotaxis,” “positive regulation of hypersensitivity,” and “positive regulation of granulocyte chemotaxis” (Figure 6(a)). Meanwhile, the most enriched Go terms in trans included “viral process,” “intracellular part,” “Golgi trans cisterna,” and “multiorganism cellular process” (Figure 6(b)). KEGG analyses in cis and in trans were performed to predict systematic biological behaviors. KEGG analysis of lncRNAs with effects in cis revealed that the top 20 significantly enriched pathways included “hypoxia Inducible Factor-1 (HIF-1) pathway,” “cytokine-cytokine receptor interaction,” “NF-*κ*B signaling pathway,” “inflammatory mediator regulation of transient receptor potential channels,” and “autoimmune thyroid disease” (Figure 6(c)). The 20 most enriched KEGG pathways of lncRNAs with effects in the trans pattern included “extracellular matrix- (ECM-) receptor interaction,” “Wnt signaling pathway,” “HTLV-I infection,” “estrogen signaling pathway,” and “focal adhesion” (Figure 6(d)). These results showed lncRNAs probably played essential roles in CAD pathogenesis by regulating inflammatory and immune responses.

### 3.6. RGSEA Analyses Based on lncRNAs Expressions

To further improve the reliability of pathways enriched of lncRNAs, RGSEA analysis was conducted to survey overall expression of cis- and trans-regulated target mRNAs, respectively. Interestingly, in the top 20 enriched pathways in KEGG analysis based on cis-regulated target mRNAs of lncRNAs, “cytokine-cytokine receptor interaction,” “NF-*κ*B signaling pathway,” “estrogen signaling pathway,” “phagosome,” “HTLV-I infection,” and “HIF-1 signaling pathway” were also enriched in RGSEA analysis (Supplementary Figure  1(A)). Additionally, “Wnt signaling pathway,” “focal adhesion,” “ECM-receptor interaction,” and “phagosome” were also enriched in RGSEA analysis (Supplementary Figure  1(B)), which were significantly enriched in the top 20 pathways in KEGG analysis based on trans-regulated target mRNAs of lncRNAs.

### 3.7. RNA-Seq Data Validation by qRT-PCR

**Figure 7:**
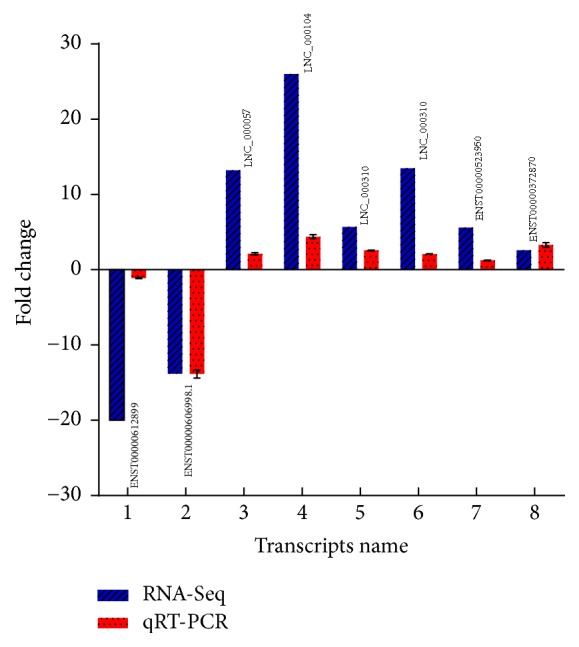
RNA-Seq and qRT-PCR outcomes of the 5 lncRNAs and 3mRNAs selected.

A total of 5 differentially expressed lncRNAs (RP11-356I2.4, LNC_000057, LNC_000104, LNC_000310, and LNC_000311) and 3 mRNA (TNFAIP3, VEGFA, and SLC27A4) were randomly selected to verify RNA-Seq data by qRT-PCR in a completely independent cohort of 4 patients with CAD and 4 healthy controls. We calculated log_2_ of fold change values for each lncRNA in qRT-PCR, comparatively with RNA-Seq data. Interestingly, qRT-PCR and RNA-Seq showed similar trends (up or downregulated) for each lncRNA. This consistency verified the accuracy and reliability of RNA-Seq findings (Figure 7).

### 3.8. Functions of Differentially Expressed lncRNAs Based on lncRNA-mRNA Coexpression and Colocation Networks

**Figure 8:**
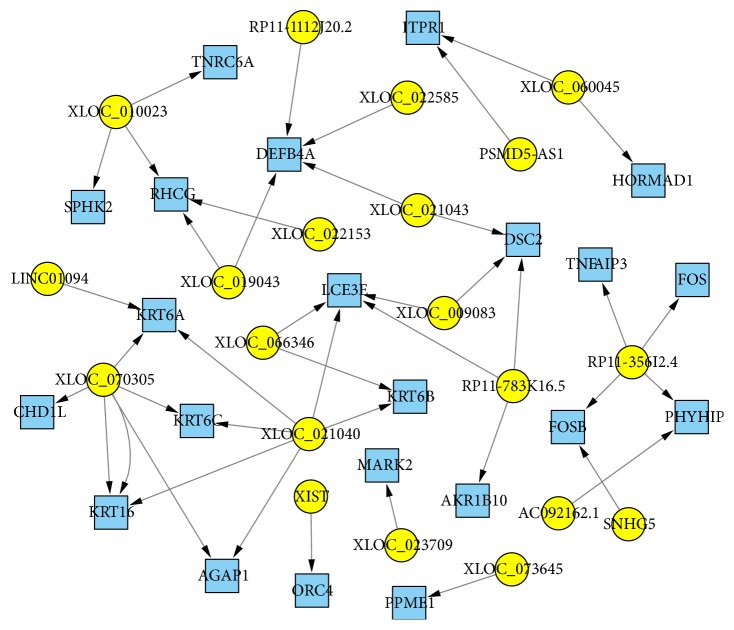
Visualization of differentially expressed lncRNA-mRNA target pairs in CAD. The nodes with blue colors represent dysregulated mRNAs, and those in yellow are dysregulated lncRNAs.

In total, 42 lncRNA-mRNA pairs were selected from the top 30 differentially expressed mRNAs (*p* < 5.93*E* − 08) (Table 2) and 30 lncRNAs, including the top 15 annotated lncRNAs (*p* < 1.41*E* − 03) and first 15 novel lncRNAs (*p* < 6.68*E* − 03). All lncRNA-mRNA pairs had correlation coefficients of more than 0.95 (*p* < 0.001; Supplementary Table S7) or regulatory associations in the cis pattern. For example, in the lncRNA-mRNA network, TNFAIP3 with the most significantly difference in mRNA levels was predicted to be regulated by the lncRNA RP11-356I2.4, which was also significantly differentially expressed. The maximum number of nodes was 4 for mRNAs and 6 for lncRNAs. These findings indicated that lncRNAs with robust associations may play pivotal roles in various regulatory networks (Figure 8).

### 3.9. The lncRNA RP11-356I2.4 Is Significantly Associated with TNFAIP3

**Figure 9:**
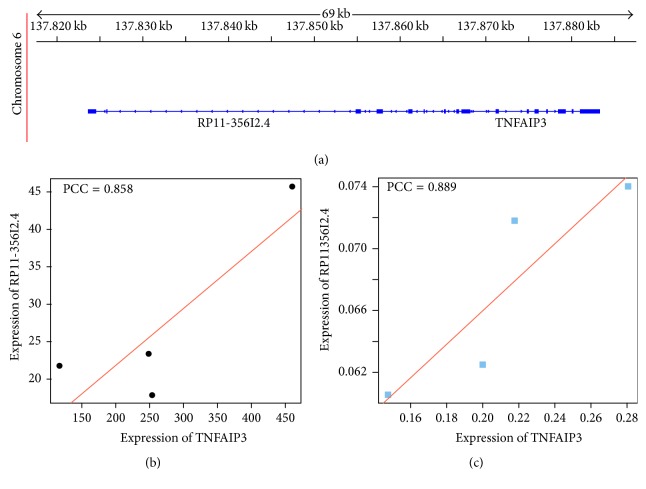
Significant correlation between lncRNA RP11-356I2.4 and TNFAIP3. (a) Genomic locations of RP11-356I2.4 and TNFAIP3. (b) Scatterplot of RP11-356I2.4 and TNFAIP3 expression levels determined by RNA-Seq in patients with CAD. (c) Scatterplot of RP11-356I2.4 and TNFAIP3 expression levels in CAD samples determined by RT-PCT.

In predicting lncRNA functions in cis (Figure 9(a)), the lncRNA RP11-356I2.4 was shown to be highly correlated with its predicted cis-regulated target TNFAIP3 (PCC = 0.858, Figure 9(b)). They are both located adjacently on chromosome 6. RT-PCR performed in an independent cohort of patients further supported their possible regulatory correlation (PCC = 0.889, Figure 9(c)). These data indicated lncRNA RP11-356I2.4 are likely to have a close relation with TNFAIP3.

## 4. Discussion

Increasing evidence demonstrates the essential roles of lncRNAs in several dermatoses [9–11]. In this study, 4401 mRNAs (including 2310 upregulated and 2091 downregulated genes) and 243 lncRNAs (198 annotated and 45 novel lncRNAs) with differential expression levels were obtained between 4 CAD samples and 4 normal controls. qRT-PCR in another 4 CAD patients and 4 healthy controls validated the findings in RNA-Seq. To the best of our knowledge, this is the first study assessing gene expression profiles by sequencing in CAD samples, identifying lncRNAs and mRNAs associated with CAD. The current findings provided an overall view of the molecular changes in CAD, with clues for subsequent CAD research.

Distinct gene expression profiles and self-segregated features in hierarchical clustering analyses were identified in patients with patients with CAD and healthy controls, suggesting the essential roles of the transcriptome and offering avenues for CAD treatment. By analyzing the dysregulated mRNAs, we found that oxidoreductase activity was included in the overrepresented GO terms. Previous research proposed that patients with CAD might suffer from defective management of oxygen radical induced damage, consistent with the above findings [28]. Among the significantly enriched KEGG pathways was the PPAR pathway, in line with the notion that CAD is immune-mediated dermatosis. Moreover, cancer related pathways were also enriched, corroborating the finding that severe CAD may evolve to cutaneous T-cell lymphoma [5].

Recent findings revealed that lncRNAs played regulatory roles in the expression of mRNAs by cis/trans patterns, rather than depending on the gene morphology and molecular structure [29]. Among the 30 most differentially expressed mRNAs and lncRNAs, respectively, 42 “lncRNAs-mRNAs” target pairs were identified, demonstrating the interactions of lncRNAs with their target mRNAs. As shown in Figure 6, most enriched GO terms, top 20 pathways in KEGG analysis based on cis-regulated target mRNAs of lncRNAs were related to inflammation and immune response, indicating the essential roles of lncRNAs in CAD pathogenesis. Among the top 20 enriched pathways for trans-regulated target mRNAs, immune response related pathways, such as ECM-receptor interaction and Wnt signaling pathway, skin barrier related pathways, and focal adhesion, were identified, in addition to the estrogen signaling pathway. This indicated that sex hormone disorders may participate in CAD development, corroborating previous clinical findings that CAD occurs more often in males [30]. Interestingly, among the enriched pathways, cytokine-cytokine receptor interaction, NF-*κ*B signaling pathway, estrogen signaling pathway, phagosome, and HIF-1 signaling pathway were enriched in both top 20 significantly enriched pathways in KEGG analysis and RGSEA analysis based on cis-regulated target mRNAs of lncRNAs, which further verified that immune response and oxidative stress play important roles in CAD development. Additionally, in pathways enriched of lncRNAs with effects in trans pattern, Wnt signaling pathway, focal adhesion, ECM-receptor interaction, and phagosome were enriched in both RGSEA analysis and top 20 pathways in KEGG analysis, further suggesting the essential role of immune response and oxidative stress, and indicate damage of skin barrier in CAD development.

We found that the HTLV-I infection pathway was enriched not only in both mRNAs and lncRNAs analyses by KEGG analysis but in lncRNAs by RGSEA analysis. Previous clinical studies demonstrated that patients with HIV infection have remarkably higher CAD morbidity, and molecular and cellular interactions of human immunodeficiency virus-1/human T-cell lymphotropic virus (HIV-1/HTLV) coinfection have also been demonstrated [31, 32], in agreement with the current findings of HTLV-I infection pathway enrichment in both mRNAs and lncRNAs.

Previous studies suggested that lncRNAs were species- and tissue-specific [33, 34]. Here, in agreement with such notions, transcript lengths of lncRNAs in exposed skin sites were reduced compared with mouse (1.65 kb on average), pig (1.45 kb on average), and zebrafish (3.34 kb on average) counterparts, with 2.84 exons, that is, more than the values for sheep (2.3 exons on average), pig (2.4 exons on average), and zebrafish (2.8 exons on average) [27, 35].

When predicting the functions of extremely differentially expressed lncRNAs, an interesting lncRNA RP11-356I2.4 was identified and highly correlated with its predicted target gene TNFAIP3. TNFAIP3 plays an essential role in the inhibition of NF-*κ*B signaling, which mediates the inflammatory response [36]. KEGG enriched pathways for both differentially expressed mRNAs and lncRNAs were found in the present study, which furthermore contribute to reduce remarkably the production of proinflammatory cytokines, including tumor necrosis factor-*α* (TNF-*α*), interleukin- (IL-) 6, and IL-1*β*, all of which are involved in CAD development, alleviating the severity of inflammatory diseases [37]. These findings suggested a potential role for TNFAIP3 in CAD development. Interestingly, both RP11-356I2.4 and TNFAIP3 were among the top 7 remarkably differentially expressed gene transcripts while comparing samples from patients with CAD and healthy individuals. TNFAIP3 and RP11-356I2.4 levels in healthy individuals were more than 19-fold (*p* = 1.12*E* − 21) and 5-fold (*p* = 1.53*E* − 04) higher than those of CAD cases, respectively. In agreement with RNA-Seq outcomes, qRT-PCR showed that RP11-356I2.4 and TNFAIP3 were both obviously downregulated in CAD. These findings suggested that RP11-356I2.4 plays an important role in the inflammatory response probably by regulating TNFAIP3.

Preciseness and reliability are important in the whole process of genome identification and analysis. Identification of lncRNAs was strictly according to a 5 step pipeline, in which only lncRNAs with transcript length ≥ 200 bp, exon number ≥ 2, and reads coverage degree ≥ 3 were included; in addition, 4 coding potential methods including CPC analysis, CNCI analysis, FFAM, and PhyloCSF were simultaneously used for lncRNA filtration, which provided more accurate data than the application of only one or two tools and helped reduce error remarkably. Additionally, expression trends of 5 differentially expressed lncRNAs and 3 mRNAs selected randomly for qRT-PCR analyses corroborated with RNA-seq findings. This consistency further verified the reliability and exactness of RNA-seq data. Additionally, expression trends of 5 differentially expressed lncRNAs and 3 mRNAs selected randomly for qRT-PCR analyses corroborated with RNA-seq findings. This consistency further verified the reliability and exactness of RNA-seq data. However, this study has some limitations. Due to limited number of sample size, there may exist observational bias; further analysis with large sample size is warranted to confirm the results observed in the present study. And RNA-Seq results and regulatory relationship of lncRNA RP11356I2.4 and TNFAIP3 should also be verified in larger cohorts of well-controlled trials, to demonstrate the functional roles of lncRNAs.

In conclusion, for the first time, lncRNA and mRNA profiles in sun-exposed skin sites of patients with CAD and healthy individuals were assessed by RNA-Seq. Bioinformatics analyses performed to comprehensively identify differentially expressed lncRNAs and mRNAs between the patients with CAD and healthy individuals indicated that inflammatory and immune response dysfunction were the essential pathogenetic mechanism of CAD. Functional analyses of lncRNAs suggested that lncRNAs might play important roles in CAD development by regulating mRNAs. These findings provide a solid foundation and valuable resource for assessing potential signaling pathways and causative genes involved in CAD.

## Supplementary Material

Supplementary Table S1: Summary of patient characteristics.
Supplementary Table S2: IDs of GTEx samples used to compare with the controls in the study.
Supplementary Table S3:Primer pairs used for RT-PCR experiments.
Supplementary Table S4: Detailed data obtained and mapping conditions for each sample in RNA-Seq.
Supplementary Table S5: Characteristics of the novel lncRNAs identified in this study.
Supplementary Table S6: KEGG pathways based on differentially expressed mRNAs between the CAD and healthy control subjects.
Supplementary Table S7: Functions of differentially expressed lncRNAs based on lncRNA-mRNA co-expression and co-location network.
Supplementary Figure S1: Pathways enriched in gene set enrichment analysis (GSEA) analysis.

## Figures and Tables

**Table 1:** Top 30 differentially expressed mRNAs with *p* < 5.93*E* − 08 between patients with CAD and controls.

Transcript ID	Gene ID	*p* value	log_2_Fold change
ENST00000612899	TNFAIP3	1.12*E* − 21	−4.30
ENST00000417353	FOSB	5.88*E* − 17	−7.13
ENST00000375530	EHMT2	1.26*E* − 16	−7.03
ENST00000252250	KRT6C	1.34*E* − 16	5.59
ENST00000442173	DOCK9	2.02*E* − 14	−6.29
ENST00000379980	MYH10	2.72*E* − 12	−5.61
ENST00000302247	DEFB4A	4.56*E* − 12	5.76
ENST00000560738	IQGAP1	5.08*E* − 12	5.64
ENST00000454243	PHYHIP	7.25*E* − 12	−3.56
ENST00000586615	FOSB	2.04*E* − 10	−4.63
ENST00000280904	DSC2	4.56*E* − 10	4.04
ENST00000623607	DCHS2	1.55*E* − 09	−5.24
ENST00000286523	ELMSAN1	2.65*E* − 09	−1.31
ENST00000396499	CCDC125	2.95*E* − 09	5.19
ENST00000368789	LCE3E	4.01*E* − 09	4.43
ENST00000354903	PER1	5.46*E* − 09	−3.06
ENST00000614409	AGAP1	8.44*E* − 09	−4.96
ENST00000220244	CEMIP	1.06*E* − 08	2.68
ENST00000356134	ANO2	1.88*E* − 08	−4.88
ENST00000555686	FOS	1.89*E* − 08	−3.92
ENST00000535373	ORC4	2.21*E* − 08	−4.89
ENST00000456211	ITPR1	2.24*E* − 08	−4.35
ENST00000615753	FOSB	2.44*E* − 08	−4.08
ENST00000218230	PCSK1N	2.65*E* − 08	−4.33
ENST00000369259	CHD1L	2.65*E* − 08	4.87
ENST00000305883	KLF11	2.94*E* − 08	−2.23
ENST00000359579	AKR1B10	3.60*E* − 08	3.58
ENST00000330722	KRT6A	4.83*E* − 08	3.88
ENST00000383710	CADPS	5.39*E* − 08	−4.77
ENST00000591858	FOSB	5.93*E* − 08	−4.36

**Table 2:** Top 15 differentially expressed annotated lncRNAs with *p* < 1.41*E* − 03 between patients with CAD and controls.

LncRNA gene name	Transcript ID	*p* value	log_2_Fold change
XIST	ENST00000429829.5	5.66*E* − 06	−3.96
RP11-21L23.2	ENST00000566747.1	9.13*E* − 06	2.92
AC092162.1	ENST00000428283.5	1.01*E* − 04	−2.59
RP11-356I2.4	ENST00000606998.1	1.53*E* − 04	−2.56
LINC01554	ENST00000436592.5	2.46*E* − 04	2.48
LINC01094	ENST00000504675.5	2.47*E* − 04	3.31
RP11-783K16.5	ENST00000544553.1	3.02*E* − 04	2.47
RUSC1-AS1	ENST00000450199.1	3.14*E* − 04	2.39
RP11-1112J20.2	ENST00000554921.1	3.58*E* − 04	2.87
SNHG5	ENST00000414002.5	3.72*E* − 04	2.35
RUSC1-AS1	ENST00000446880.5	6.10*E* − 04	2.18
RP3-429O6.1	ENST00000422310.2	9.61*E* − 04	2.64
PSMD5-AS1	ENST00000614216.4	1.12*E* − 03	2.54
RP11-195F19.30	ENST00000564224.1	1.35*E* − 03	−2.07
XIST	ENST00000602587.5	1.41*E* − 03	−2.88

**Table 3:** Top 15 differentially expressed novel lncRNAs with *p* < 6.68*E* − 03 between the patients with CAD and controls.

LncRNA gene name	*p* value	log_2_Fold change
LNC_000310	8.31*E* − 09	4.66
LNC_000311	4.14*E* − 06	3.73
LNC_000262	5.97*E* − 06	−3.35
LNC_000057	1.11*E* − 05	3.74
LNC_000312	2.86*E* − 05	3.54
LNC_000099	2.85*E* − 04	2.92
LNC_000030	3.11*E* − 04	2.21
LNC_000291	8.68*E* − 04	2.88
LNC_000104	9.54*E* − 04	2.50
LNC_000089	1.15*E* − 03	2.46
LNC_000097	1.92*E* − 03	2.57
LNC_000329	2.14*E* − 03	2.43
LNC_000102	3.46*E* − 03	2.33
LNC_000049	4.10*E* − 03	2.62
LNC_000098	6.68*E* − 03	2.33

**Table 4:** Properties of immune-related genes in the top 10 obviously differentially expressed mRNA transcripts.

Gene name	Immunological function
TNFAIP3	Play important role in inflammatory pathways, including NF-*κ*B pathway and TLR signaling
FOSB	Involved in the control of immune by regulatory relationships with P2RX7, NO production, and so on
EHMT2	Involved in immune response and take effect in and epigenetic modifications and so on
KRT6C	None
DOCK9	Play an important role in dendrite growth in hippocampal neurons and mediating TGF-1/Smad4 activation and so on
MYH10	None
DEFB4A	Participate in antimicrobial peptide-mediated inflammation
IQGAP1	Stimulate cell migration which impact immune surveillance and regulate granule polarization in NK cells
PHYHIP	None
FOSB	Involved in the control of immune by regulatory relationships with P2RX7, NO production, and so on
