# Supplementary material for: Genome-Wide Analysis of mRNA and Long Noncoding RNA Profiles in Chronic Actinic Dermatitis
Source: Biomed Res Int. 2017 Nov 14;2017:7479523. doi: 10.1155/2017/7479523 (PMC5735319; doi:10.1155/2017/7479523)
Supplement: Supplementary file 1 — Supplementary Table S1: Summary of patient characteristics. Supplementary Table S2: IDs of GTEx samples used to compare with the controls in the study. Supplementary Table S3:Primer pairs used for RT-PCR experiments. Supplementary Table S4: Detailed data obtained and mapping conditions for each sample in RNA-Seq. Supplementary Table S5: Characteristics of the novel lncRNAs identified in this study. Supplementary Table S6: KEGG pathways based on differentially expressed mRNAs between the CAD and healthy control subjects. Supplementary Table S7: Functions of differentially expressed lncRNAs based on lncRNA-mRNA co-expression and co-location network. Supplementary Figure S1: Pathways enriched in gene set enrichment analysis (GSEA) analysis. [file 7479523.f1.pdf]

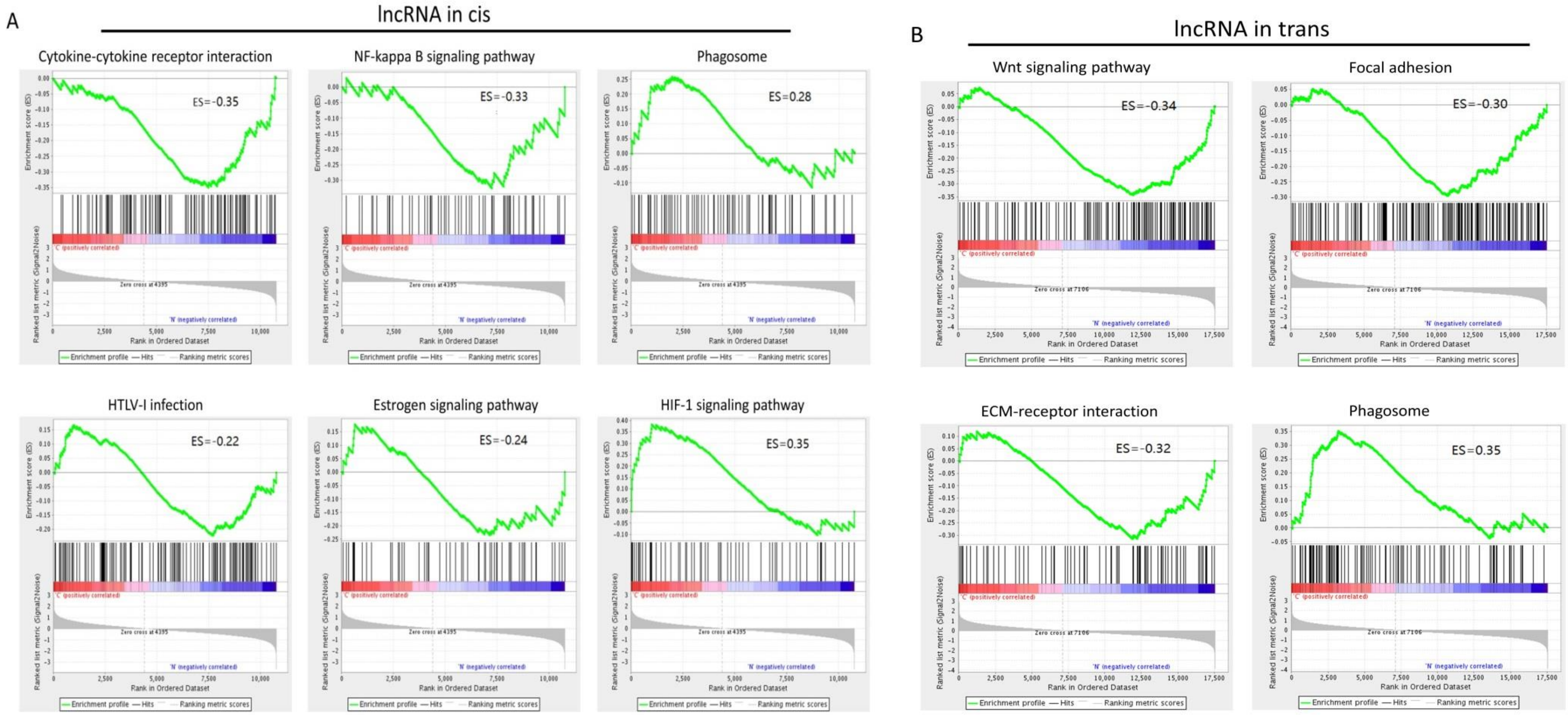

Supplementary figure S1. Pathways enriched in gene set enrichment analysis (GSEA) analysis. (A) Pathways enriched in both GSEA analysis and top 20 enriched pathways in KEGG analysis based on lncRNAs taking effect in cis. (B) Pathways enriched in both GSEA analysis and top 20 enriched pathways in KEGG analysis based on lncRNAs taking effect in trans.

Supplementary Table S1. Summary of patient characteristics.

	CAD	Control	P-value
Total no. of subjects	8	8	
Sex, male, n (%)	8(100.0)	6(75.0)	0.467*
Age (y), mean ± SD	59.9±9.5	55.3±8.6	0.324**
Methodology used			
RNA sequencing	n=4	n=4	
Age (y), mean ± SD	65.0±10.7	54.3±6.1	0.131**
Sex, male, n (%)	4(100.0)	2(50)	0.429*
qRT-PCR	n=4	n=4	
Age (y), mean ± SD	54.8±5.0	56.3±11.5	0.819**

*Statistical analyses were conducted using Fisher exact test.

**Statistical analyses were conducted using student's t-test.

Supplementary Table S2. IDs of GTEx samples used to compare with the controls in the study.

SAMPID	SMATSSCR	SMCENTER	SMRIN	SMTSD	SMUBRID	SMTSISCH	SMTSPAX	SMNABTCH	SMGEBTCH	SMGEBTCHT
GTEX-111CU-1926-SM-5GZYZ	1	B1	5.9	Skin - Sun Exposed	1511	141	568	BP-43783	LCSET-4804	TrueSeq.v1
GTEX-111FC-0126-SM-5N9DL	1	B1	7.2	Skin - Sun Exposed	1511	1036	870	BP-43989	LCSET-4904	TrueSeq.v1
GTEX-111VG-2426-SM-5GZXD	1	B1	7.4	Skin - Sun Exposed	1511	1084	873	BP-43693	LCSET-4804	TrueSeq.v1
GTEX-1122O-2126-SM-5EGIR	0	B1	7.1	Skin - Sun Exposed	1511	128	875	BP-43529	LCSET-4764	TrueSeq.v1
GTEX-1128S-2326-SM-5GZZY	0	B1	7	Skin - Sun Exposed	1511	886	959	BP-43783	LCSET-4805	TrueSeq.v1
GTEX-113IC-0126-SM-5HL6T	0	B1	7.2	Skin - Sun Exposed	1511	94	1080	BP-43529	LCSET-4806	TrueSeq.v1
GTEX-113JC-2326-SM-5EQ4E	1	B1	7.5	Skin - Sun Exposed	1511	681	940	BP-44460	LCSET-4767	TrueSeq.v1
GTEX-117XS-2726-SM-5N9BL	1	B1	6.9	Skin - Sun Exposed	1511	916	538	BP-43989	LCSET-4904	TrueSeq.v1
GTEX-117YW-2626-SM-5GZZH	1	B1	6.8	Skin - Sun Exposed	1511	982	543	BP-43507	LCSET-4805	TrueSeq.v1
GTEX-117YX-2326-SM-5H12W	0	B1	6.4	Skin - Sun Exposed	1511	147	659	BP-43783	LCSET-4805	TrueSeq.v1
GTEX-1192W-2626-SM-5Q5AF	0	B1	5.8	Skin - Sun Exposed	1511	810	500	BP-44261	LCSET-4952	TrueSeq.v1
GTEX-1192X-0226-SM-5H12D	0	B1	6	Skin - Sun Exposed	1511	856	946	BP-43783	LCSET-4805	TrueSeq.v1
GTEX-11DXW-0226-SM-5H122	1	C1	7.2	Skin - Sun Exposed	1511	1089	1347	BP-43730	LCSET-4805	TrueSeq.v1
GTEX-11DXX-2426-SM-5GZZW	1	B1	6.8	Skin - Sun Exposed	1511	161	452	BP-43783	LCSET-4805	TrueSeq.v1
GTEX-11DXY-2626-SM-5GIE7	1	B1	6.6	Skin - Sun Exposed	1511	963	1077	BP-44261	LCSET-4796	TrueSeq.v1
GTEX-11DXZ-2326-SM-5EGGV	1	B1	8.1	Skin - Sun Exposed	1511	328	873	BP-44460	LCSET-4764	TrueSeq.v1
GTEX-11DYG-0126-SM-59883	0	C1	7.7	Skin - Sun Exposed	1511	781	926	BP-44577	LCSET-4417	TrueSeq.v1
GTEX-11DZ1-0126-SM-5985Q	1	C1	7.7	Skin - Sun Exposed	1511	1193	610	BP-44902	LCSET-4417	TrueSeq.v1
GTEX-11EI6-0126-SM-5985R	0	C1	7.4	Skin - Sun Exposed	1511	969	1438	BP-44902	LCSET-4417	TrueSeq.v1
GTEX-11EM3-2426-SM-59861	1	B1	7.9	Skin - Sun Exposed	1511	240	428	BP-44902	LCSET-4417	TrueSeq.v1
GTEX-11EMC-2926-SM-5EQ4F	0	B1	6.4	Skin - Sun Exposed	1511	809	591	BP-44460	LCSET-4767	TrueSeq.v1
GTEX-11EQ8-0126-SM-5EQ54	1	C1	6.8	Skin - Sun Exposed	1511	460	647	BP-44460	LCSET-4767	TrueSeq.v1
GTEX-11EQ9-2426-SM-5HL5T	0	B1	7.5	Skin - Sun Exposed	1511	321	378	BP-43730	LCSET-4806	TrueSeq.v1
GTEX-11GS4-2726-SM-5A5LE	0	B1	7.7	Skin - Sun Exposed	1511	915	529	BP-44512	LCSET-4423	TrueSeq.v1

SAMPID	SMATSSCR	SMCENTER	SMRIN	SMTSD	SMUBRID	SMTSISCH	SMTSPAX	SMNABTCH	SMGEBTCH	SMGEBTCHT
GTEX-11GSO-2426-SM-5A5LY	1	B1	6.8	Skin - Sun Exposed	1511	969	580	BP-44512	LCSET-4423	TrueSeq.v1
GTEX-11GSP-2526-SM-5N9BP	1	B1	7.4	Skin - Sun Exposed	1511	920	416	BP-43989	LCSET-4904	TrueSeq.v1
GTEX-11H98-0126-SM-5EGHG	1	B1	7.8	Skin - Sun Exposed	1511	733	598	BP-43495	LCSET-4764	TrueSeq.v1
GTEX-11ILO-0126-SM-5A5LN	0	C1	7.1	Skin - Sun Exposed	1511	372	1103	BP-44512	LCSET-4423	TrueSeq.v1
GTEX-11LCK-1026-SM-5A5KB	0	B1	7.8	Skin - Sun Exposed	1511	259	1078	BP-44264	LCSET-4423	TrueSeq.v1
GTEX-11NSD-2226-SM-5986V	0	B1	7.2	Skin - Sun Exposed	1511	287	849	BP-44902	LCSET-4417	TrueSeq.v1
GTEX-11NUK-0126-SM-5A5L3	1	B1	7.4	Skin - Sun Exposed	1511	894	887	BP-44264	LCSET-4423	TrueSeq.v1
GTEX-11O72-0126-SM-59887	0	C1	7.5	Skin - Sun Exposed	1511	922	983	BP-44902	LCSET-4417	TrueSeq.v1
GTEX-11OC5-0126-SM-5HL6A	0	B1	6.5	Skin - Sun Exposed	1511	1272	914	BP-43730	LCSET-4806	TrueSeq.v1
GTEX-11OF3-2626-SM-5GU7F	0	B1	8.3	Skin - Sun Exposed	1511	887	917	BP-44312	LCSET-5132	TrueSeq.v1
GTEX-11P7K-2126-SM-5GU7G	0	B1	6.8	Skin - Sun Exposed	1511	351	818	BP-44312	LCSET-4803	TrueSeq.v1
GTEX-11P81-2326-SM-59875	0	B1	8.4	Skin - Sun Exposed	1511	350	525	BP-44902	LCSET-4417	TrueSeq.v1
GTEX-11P82-1626-SM-59879	0	B1	6.2	Skin - Sun Exposed	1511	194	862	BP-44902	LCSET-4417	TrueSeq.v1
GTEX-11TT1-2526-SM-5EGIB	0	B1	7.7	Skin - Sun Exposed	1511	135	400	BP-45028	LCSET-4764	TrueSeq.v1
GTEX-11TTK-0126-SM-5987B	1	C1	6.8	Skin - Sun Exposed	1511	1122	784	BP-44902	LCSET-4417	TrueSeq.v1
GTEX-11TUW-2726-SM-5EQLC	1	B1	6.5	Skin - Sun Exposed	1511	824	871	BP-44114	LCSET-4790	TrueSeq.v1
GTEX-11UD1-0126-SM-5PNYG	1	B1	5.7	Skin - Sun Exposed	1511	1357	1116	BP-47888	LCSET-4918	TrueSeq.v1
GTEX-11UD2-0226-SM-5EQKY	0	B1	7.4	Skin - Sun Exposed	1511	845	1339	BP-44114	LCSET-4790	TrueSeq.v1
GTEX-11VI4-1726-SM-5CVLH	0	B1	6.7	Skin - Sun Exposed	1511	315	958	BP-44525	LCSET-4633	TrueSeq.v1
GTEX-11WQC-2526-SM-5CVLE	1	B1	6.8	Skin - Sun Exposed	1511	818	540	BP-44525	LCSET-4633	TrueSeq.v1
GTEX-11WQK-0526-SM-5EQLD	0	B1	7.8	Skin - Sun Exposed	1511	731	945	BP-44114	LCSET-4790	TrueSeq.v1
GTEX-11XUK-2026-SM-5EQLF	0	B1	7.5	Skin - Sun Exposed	1511	334	1107	BP-44114	LCSET-4790	TrueSeq.v1
GTEX-11ZTS-0126-SM-5EQ6M	0	B1	7.4	Skin - Sun Exposed	1511	599	866	BP-45028	LCSET-4767	TrueSeq.v1
GTEX-11ZTT-2426-SM-5EQLS	1	B1	7.3	Skin - Sun Exposed	1511	247	1076	BP-44114	LCSET-4790	TrueSeq.v1
GTEX-11ZU8-2526-SM-5EQKI	0	B1	7.9	Skin - Sun Exposed	1511	1020	426	BP-44312	LCSET-4790	TrueSeq.v1

SAMPID	SMATSSCR	SMCENTER	SMRIN	SMTSD	SMUBRID	SMTSISCH	SMTSPAX	SMNABTCH	SMGEBTCH	SMGEBTCHT
GTEX-11ZUS-0626-SM-59885	1	C1	8.4	Skin - Sun Exposed	1511	1359	1131	BP-44577	LCSET-4417	TrueSeq.v1
GTEX-11ZVC-2526-SM-5FQTE	0	B1	8.2	Skin - Sun Exposed	1511	640	517	BP-43823	LCSET-4793	TrueSeq.v1
GTEX-1211K-2026-SM-5EQ4Z	0	B1	7.5	Skin - Sun Exposed	1511	145	411	BP-44399	LCSET-4767	TrueSeq.v1
GTEX-12126-0326-SM-5PNW3	1	C1	6.4	Skin - Sun Exposed	1511	927	957	BP-47888	LCSET-4917	TrueSeq.v1
GTEX-1212Z-2526-SM-5EGKP	1	B1	7.6	Skin - Sun Exposed	1511	757	1005	BP-45028	LCSET-4797	TrueSeq.v1
GTEX-12584-0126-SM-5EGKQ	1	B1	8.7	Skin - Sun Exposed	1511	926	615	BP-45028	LCSET-4797	TrueSeq.v1
GTEX-12696-2426-SM-5EQ6H	0	B1	7.1	Skin - Sun Exposed	1511	600	1095	BP-44525	LCSET-4767	TrueSeq.v1
GTEX-1269C-2526-SM-5EGJ3	1	B1	7.5	Skin - Sun Exposed	1511	985	600	BP-44399	LCSET-4797	TrueSeq.v1
GTEX-12C56-1726-SM-5EQ61	1	B1	6.6	Skin - Sun Exposed	1511	241	1190	BP-44399	LCSET-4767	TrueSeq.v1
GTEX-12KS4-0326-SM-5EQ41	0	C1	8.4	Skin - Sun Exposed	1511	464	988	BP-44399	LCSET-4767	TrueSeq.v1
GTEX-12WS9-0126-SM-5EGL7	0	C1	8.4	Skin - Sun Exposed	1511	496	1193	BP-45028	LCSET-4797	TrueSeq.v1
GTEX-12WSA-0226-SM-5BC5V	0	C1	6.2	Skin - Sun Exposed	1511	934	1417	BP-43916	LCSET-4455	TrueSeq.v1
GTEX-12WSB-0126-SM-59HJN	0	C1	7.8	Skin - Sun Exposed	1511	1106	548	BP-44902	LCSET-4419	TrueSeq.v1
GTEX-12WSC-0126-SM-5EQ4P	1	C1	8.3	Skin - Sun Exposed	1511	787	479	BP-44399	LCSET-4767	TrueSeq.v1
GTEX-12WSD-0126-SM-59HKN	0	C1	6.2	Skin - Sun Exposed	1511	484	801	BP-44577	LCSET-4419	TrueSeq.v1
GTEX-12WSG-2826-SM-5FQUS	1	B1	7.7	Skin - Sun Exposed	1511	427	1204	BP-44157	LCSET-4793	TrueSeq.v1
GTEX-12WSJ-1826-SM-5GCNY	1	B1	7.3	Skin - Sun Exposed	1511	208	1018	BP-43881	LCSET-4795	TrueSeq.v1
GTEX-12WSK-2326-SM-5GCOH	0	B1	7.7	Skin - Sun Exposed	1511	208	378	BP-44157	LCSET-4795	TrueSeq.v1
GTEX-12WSL-2526-SM-5GCNJ	1	B1	8.1	Skin - Sun Exposed	1511	353	500	BP-44157	LCSET-4795	TrueSeq.v1
GTEX-12WSN-2326-SM-5DUVG	1	B1	8	Skin - Sun Exposed	1511	290	599	BP-44437	LCSET-4634	TrueSeq.v1
GTEX-12ZZW-0126-SM-5DUVB	0	C1	6	Skin - Sun Exposed	1511	1090	946	BP-44525	LCSET-4634	TrueSeq.v1
GTEX-12ZZX-0126-SM-5EGK9	0	C1	7.9	Skin - Sun Exposed	1511	584	718	BP-44901	LCSET-4797	TrueSeq.v1
GTEX-12ZZY-0226-SM-5LZVE	0	C1	8.7	Skin - Sun Exposed	1511	510	826	BP-43956	LCSET-4900	TrueSeq.v1
GTEX-12ZZZ-0626-SM-5DUXI	0	B1	7.3	Skin - Sun Exposed	1511	822	1063	BP-44437	LCSET-4634	TrueSeq.v1
GTEX-13111-1626-SM-5EGJY	1	B1	7.4	Skin - Sun Exposed	1511	321	448	BP-44901	LCSET-4797	TrueSeq.v1

SAMPID	SMATSSCR	SMCENTER	SMRIN	SMTSD	SMUBRID	SMTSISCH	SMTSPAX	SMNABTCH	SMGEBTCH	SMGEBTCHT
GTEX-13112-2826-SM-5DUWH	1	B1	8	Skin - Sun Exposed	1511	1355	496	BP-44437	LCSET-4634	TrueSeq.v1
GTEX-13113-1926-SM-5LZWG	0	B1	7.5	Skin - Sun Exposed	1511	296	798	BP-43956	LCSET-4900	TrueSeq.v1
GTEX-1313W-0126-SM-5LZUN	1	C1	7.2	Skin - Sun Exposed	1511	618	690	BP-45782	LCSET-4900	TrueSeq.v1
GTEX-1314G-1526-SM-5EGK2	0	B1	8	Skin - Sun Exposed	1511	477	759	BP-44901	LCSET-4797	TrueSeq.v1
GTEX-131XE-2326-SM-5PNZ2	1	B1	7.1	Skin - Sun Exposed	1511	240	613	BP-47901	LCSET-4918	TrueSeq.v1
GTEX-131XF-2126-SM-5DUWS	0	B1	6.8	Skin - Sun Exposed	1511	380	1124	BP-44437	LCSET-4634	TrueSeq.v1
GTEX-131XG-2626-SM-5KM17	0	B1	6.1	Skin - Sun Exposed	1511	258	529	BP-45499	LCSET-4820	TrueSeq.v1
GTEX-131XH-2226-SM-5DUXJ	0	B1	7.7	Skin - Sun Exposed	1511	1217	560	BP-44437	LCSET-4634	TrueSeq.v1
GTEX-131XW-0426-SM-5LZWP	1	C1	7.1	Skin - Sun Exposed	1511	655	948	BP-45782	LCSET-4900	TrueSeq.v1
GTEX-131YS-0126-SM-5KM1M	1	C1	7.2	Skin - Sun Exposed	1511	942	1013	BP-46482	LCSET-4820	TrueSeq.v1
GTEX-132NY-0326-SM-5IJBL	0	C1	6.7	Skin - Sun Exposed	1511	492	1052	BP-45499	LCSET-4814	TrueSeq.v1
GTEX-133LE-1826-SM-5J1MV	0	C1	6.7	Skin - Sun Exposed	1511	301	1272	BP-46482	LCSET-4816	TrueSeq.v1
GTEX-1399R-2726-SM-5IJBA	1	B1	7.6	Skin - Sun Exposed	1511	334	873	BP-46482	LCSET-4814	TrueSeq.v1
GTEX-1399T-2826-SM-5KLYZ	1	B1	7.5	Skin - Sun Exposed	1511	947	898	BP-46589	LCSET-4820	TrueSeq.v1
GTEX-1399U-2326-SM-5K7WY	0	B1	5.8	Skin - Sun Exposed	1511	362	528	BP-45499	LCSET-4819	TrueSeq.v1
GTEX-139D8-0226-SM-5KLZO	0	C1	7.5	Skin - Sun Exposed	1511	417	1003	BP-46589	LCSET-4820	TrueSeq.v1
GTEX-139T4-0226-SM-5HL5S	1	B1	6.4	Skin - Sun Exposed	1511	1352	1238	BP-45268	LCSET-4806	TrueSeq.v1
GTEX-139T8-0126-SM-5K7XZ	0	C1	7.2	Skin - Sun Exposed	1511	574	596	BP-45499	LCSET-4819	TrueSeq.v1
GTEX-139TS-2826-SM-5J1OO	1	B1	7.3	Skin - Sun Exposed	1511	1302	942	BP-46925	LCSET-4816	TrueSeq.v1
GTEX-139TT-0126-SM-5K7Y5	0	B1	8	Skin - Sun Exposed	1511	1165	1202	BP-45485	LCSET-4819	TrueSeq.v1
GTEX-139TU-0126-SM-5K7WH	1	C1	7.7	Skin - Sun Exposed	1511	527	830	BP-46925	LCSET-4818	TrueSeq.v1
GTEX-139UW-2826-SM-5L3E7	0	B1	7.7	Skin - Sun Exposed	1511	845	425	BP-46589	LCSET-4822	TrueSeq.v1
GTEX-13CF2-2526-SM-5LZYX	0	B1	7.3	Skin - Sun Exposed	1511	1427	586	BP-45782	LCSET-4901	TrueSeq.v1
GTEX-13CF3-2426-SM-5IFGX	1	B1	7.5	Skin - Sun Exposed	1511	213	360	BP-46589	LCSET-4813	TrueSeq.v1
GTEX-13D11-2426-SM-5KM1V	0	B1	8	Skin - Sun Exposed	1511	166	1119	BP-45499	LCSET-4820	TrueSeq.v1

SAMPID	SMATSSCR	SMCENTER	SMRIN	SMTSD	SMUBRID	SMTSISCH	SMTSPAX	SMNABTCH	SMGEBTCH	SMGEBTCHT
GTEX-13FH7-2026-SM-5IJCR	1	B1	7.3	Skin - Sun Exposed	1511	256	729	BP-46680	LCSET-4814	TrueSeq.v1
GTEX-13FHO-0226-SM-5IFGY	1	C1	7.2	Skin - Sun Exposed	1511	631	514	BP-46589	LCSET-4813	TrueSeq.v1
GTEX-13FHP-0126-SM-5IJBG	1	C1	8	Skin - Sun Exposed	1511	827	1106	BP-46978	LCSET-4814	TrueSeq.v1
GTEX-13FTW-2626-SM-5K7WL	1	B1	6.4	Skin - Sun Exposed	1511	371	470	BP-46943	LCSET-4819	TrueSeq.v1
GTEX-13FTX-1826-SM-5J1NX	1	B1	6.2	Skin - Sun Exposed	1511	213	1044	BP-46453	LCSET-4816	TrueSeq.v1
GTEX-13FTY-0526-SM-5L3EV	1	B1	6	Skin - Sun Exposed	1511	727	597	BP-46650	LCSET-4822	TrueSeq.v1
GTEX-13FXS-0226-SM-5IJCK	1	B1	6.8	Skin - Sun Exposed	1511	566	1046	BP-46943	LCSET-4814	TrueSeq.v1
GTEX-13G51-2526-SM-5LZYK	1	B1	8.2	Skin - Sun Exposed	1511	925	835	BP-46529	LCSET-4901	TrueSeq.v1
GTEX-13IVO-0126-SM-5J1NG	1	C1	6.7	Skin - Sun Exposed	1511	556	1202	BP-46650	LCSET-4816	TrueSeq.v1
GTEX-13JUV-2726-SM-5LZZ8	1	B1	7.8	Skin - Sun Exposed	1511	586	1098	BP-46529	LCSET-4901	TrueSeq.v1
GTEX-13N11-2526-SM-5K7UE	1	B1	7.2	Skin - Sun Exposed	1511	228	971	BP-46978	LCSET-4818	TrueSeq.v1
GTEX-13N1W-0126-SM-5K7VT	1	C1	7.4	Skin - Sun Exposed	1511	612	700	BP-47051	LCSET-4818	TrueSeq.v1
GTEX-13N2G-2626-SM-5J1OT	1	B1	7.1	Skin - Sun Exposed	1511	589	889	BP-46650	LCSET-4816	TrueSeq.v1
GTEX-13NYB-2526-SM-5K7Z6	0	B1	7.1	Skin - Sun Exposed	1511	1153	871	BP-46680	LCSET-4819	TrueSeq.v1
GTEX-13NYC-0326-SM-5K7WP	1	B1	6.7	Skin - Sun Exposed	1511	970	703	BP-46734	LCSET-4819	TrueSeq.v1
GTEX-13NYS-0126-SM-5MR3W	1	B1	7.7	Skin - Sun Exposed	1511	846	804	BP-46529	LCSET-4902	TrueSeq.v1
GTEX-13NZ8-0826-SM-5MR3I	1	C1	7.3	Skin - Sun Exposed	1511	1345	482	BP-46529	LCSET-4902	TrueSeq.v1
GTEX-13NZA-0226-SM-5K7Z7	2	B1	6.5	Skin - Sun Exposed	1511	1327	528	BP-46978	LCSET-4819	TrueSeq.v1
GTEX-13NZB-2526-SM-5J1OM	1	B1	6.7	Skin - Sun Exposed	1511	395	758	BP-46925	LCSET-4816	TrueSeq.v1
GTEX-13O1R-0126-SM-5L3EH	1	B1	8.4	Skin - Sun Exposed	1511	801	489	BP-47051	LCSET-4822	TrueSeq.v1
GTEX-13O21-1426-SM-5K7XI	0	B1	6	Skin - Sun Exposed	1511	222	504	BP-46978	LCSET-4819	TrueSeq.v1
GTEX-13O3O-0126-SM-5KM4E	0	C1	6.6	Skin - Sun Exposed	1511	778	925	BP-47141	LCSET-4821	TrueSeq.v1
GTEX-13O3P-0126-SM-5KM4S	0	C1	7.4	Skin - Sun Exposed	1511	405	1239	BP-47524	LCSET-4821	TrueSeq.v1
GTEX-13O3Q-2426-SM-5IJBR	1	B1	5.8	Skin - Sun Exposed	1511	1157	688	BP-47125	LCSET-4814	TrueSeq.v1
GTEX-13OVG-1926-SM-5L3DK	0	B1	7.6	Skin - Sun Exposed	1511	644	618	BP-47125	LCSET-4822	TrueSeq.v1

SAMPID	SMATSSCR	SMCENTER	SMRIN	SMTSD	SMUBRID	SMTSISCH	SMTSPAX	SMNABTCH	SMGEBTCH	SMGEBTCHT
GTEX-13OVH-0126-SM-5MR4B	0	C1	7.6	Skin - Sun Exposed	1511	562	1426	BP-47656	LCSET-4902	TrueSeq.v1
GTEX-13OVI-1426-SM-5L3EY	0	B1	8.7	Skin - Sun Exposed	1511	187	519	BP-47070	LCSET-4822	TrueSeq.v1
GTEX-13OVJ-0126-SM-5KM36	1	B1	6.7	Skin - Sun Exposed	1511	1117	816	BP-47524	LCSET-4821	TrueSeq.v1
GTEX-13OVL-0226-SM-5L3E9	1	B1	8.4	Skin - Sun Exposed	1511	855	1258	BP-47125	LCSET-4822	TrueSeq.v1
GTEX-13OW5-0126-SM-5MR3A	0	B1	7.8	Skin - Sun Exposed	1511	843	679	BP-47656	LCSET-4902	TrueSeq.v1
GTEX-13OW6-0426-SM-5L3HM	1	C1	7.8	Skin - Sun Exposed	1511	558	1283	BP-47486	LCSET-4823	TrueSeq.v1
GTEX-13OW7-0126-SM-5MR3B	0	C1	7.3	Skin - Sun Exposed	1511	802	897	BP-47656	LCSET-4902	TrueSeq.v1
GTEX-13OW8-1326-SM-5J1OW	1	B1	7.4	Skin - Sun Exposed	1511	1093	697	BP-47125	LCSET-4816	TrueSeq.v1
GTEX-13PDP-0126-SM-5L3HD	1	C1	6.7	Skin - Sun Exposed	1511	842	1068	BP-47486	LCSET-4823	TrueSeq.v1
GTEX-13PVQ-0126-SM-5SIB4	0	C1	7.4	Skin - Sun Exposed	1511	846	1177	BP-47656	LCSET-5174	TrueSeq.v1
GTEX-13PVR-2326-SM-5QGR1	0	B1	7.6	Skin - Sun Exposed	1511	242	565	BP-48448	LCSET-4954	TrueSeq.v1
GTEX-13RTJ-2426-SM-5S2Q2	1	B1	6.7	Skin - Sun Exposed	1511	930	1025	BP-48647	LCSET-4988	TrueSeq.v1
GTEX-13S7M-0126-SM-5SI6A	1	C1	7.3	Skin - Sun Exposed	1511	575	956	BP-48647	LCSET-4995	TrueSeq.v1
GTEX-13SLW-0126-SM-5SI6C	0	B1	6.1	Skin - Sun Exposed	1511	1187	713	BP-48647	LCSET-4995	TrueSeq.v1
GTEX-13SLX-0526-SM-5S2ON	1	B1	8	Skin - Sun Exposed	1511	1063	571	BP-48564	LCSET-4988	TrueSeq.v1
GTEX-13U4I-0126-SM-5LU38	0	C1	7.1	Skin - Sun Exposed	1511	396	1048	BP-47524	LCSET-4824	TrueSeq.v1
GTEX-13VXT-0126-SM-5LU4I	1	B1	7.5	Skin - Sun Exposed	1511	438	828	BP-47141	LCSET-4824	TrueSeq.v1
GTEX-13VXU-0126-SM-5SI9F	1	B1	7.2	Skin - Sun Exposed	1511	920	626	BP-47656	LCSET-5174	TrueSeq.v1
GTEX-13W3W-2426-SM-5LU5B	0	B1	6.5	Skin - Sun Exposed	1511	497	1030	BP-47486	LCSET-4824	TrueSeq.v1
GTEX-13W46-0226-SM-5SIAG	1	C1	7.4	Skin - Sun Exposed	1511	493	463	BP-47656	LCSET-5174	TrueSeq.v1
GTEX-13X6I-0626-SM-5QGP9	1	C1	7.8	Skin - Sun Exposed	1511	807	1342	BP-48057	LCSET-4954	TrueSeq.v1
GTEX-13X6J-0126-SM-5QGOU	1	C1	7.8	Skin - Sun Exposed	1511	1251	1145	BP-48057	LCSET-4954	TrueSeq.v1
GTEX-13YAN-0326-SM-5O9DF	0	C1	8.2	Skin - Sun Exposed	1511	601	1198	BP-47675	LCSET-4910	TrueSeq.v1
GTEX-144GN-2326-SM-5LU4F	0	B1	6.8	Skin - Sun Exposed	1511	251	452	BP-47591	LCSET-4824	TrueSeq.v1
GTEX-145LS-0126-SM-5QGP1	0	C1	7.6	Skin - Sun Exposed	1511	848	731	BP-48057	LCSET-4954	TrueSeq.v1

SAMPID	SMATSSCR	SMCENTER	SMRIN	SMTSD	SMUBRID	SMTSISCH	SMTSPAX	SMNABTCH	SMGEBTCH	SMGEBTCHT
GTEX-145LT-1726-SM-5QGP3	0	B1	7.2	Skin - Sun Exposed	1511	441	427	BP-48057	LCSET-4954	TrueSeq.v1
GTEX-145LV-2126-SM-5LU8N	1	B1	6.9	Skin - Sun Exposed	1511	476	703	BP-47591	LCSET-4899	TrueSeq.v1
GTEX-145ME-1826-SM-5SI9G	0	B1	7.5	Skin - Sun Exposed	1511	315	951	BP-47616	LCSET-5174	TrueSeq.v1
GTEX-145MF-0626-SM-5LUAI	0	B1	6.3	Skin - Sun Exposed	1511	934	985	BP-47616	LCSET-4899	TrueSeq.v1
GTEX-145MI-0526-SM-5O9A8	0	B1	7.8	Skin - Sun Exposed	1511	702	893	BP-47696	LCSET-4909	TrueSeq.v1
GTEX-145MN-2226-SM-5SIAU	1	B1	6.6	Skin - Sun Exposed	1511	237	620	BP-47616	LCSET-5174	TrueSeq.v1
GTEX-145MO-0326-SM-5Q5B3	0	C1	7.6	Skin - Sun Exposed	1511	658	512	BP-48437	LCSET-4952	TrueSeq.v1
GTEX-146FH-0126-SM-5QGPP	1	C1	8.1	Skin - Sun Exposed	1511	396	643	BP-48068	LCSET-4954	TrueSeq.v1
GTEX-146FQ-0126-SM-5NQA9	1	B1	6.3	Skin - Sun Exposed	1511	474	817	BP-47696	LCSET-4907	TrueSeq.v1
GTEX-146FR-1326-SM-5SIAJ	0	B1	8	Skin - Sun Exposed	1511	1635	809	BP-47616	LCSET-5174	TrueSeq.v1
GTEX-14753-0426-SM-5NQAM	1	B1	5.9	Skin - Sun Exposed	1511	808	820	BP-47696	LCSET-4907	TrueSeq.v1
GTEX-147F3-0526-SM-5SI98	1	B1	7	Skin - Sun Exposed	1511	486	1349	BP-47616	LCSET-5174	TrueSeq.v1
GTEX-147F4-0226-SM-5QGPW	0	B1	7	Skin - Sun Exposed	1511	1225	585	BP-48068	LCSET-4954	TrueSeq.v1
GTEX-147GR-0526-SM-5S2MY	0	B1	7	Skin - Sun Exposed	1511	999	871	BP-48564	LCSET-4988	TrueSeq.v1
GTEX-148VI-1626-SM-5SI6E	1	B1	7.2	Skin - Sun Exposed	1511	417	1038	BP-48651	LCSET-4995	TrueSeq.v1
GTEX-14A5H-0426-SM-5QGQF	0	B1	7.3	Skin - Sun Exposed	1511	525	575	BP-48437	LCSET-4954	TrueSeq.v1
GTEX-14A6H-0126-SM-5QGPK	0	B1	6.5	Skin - Sun Exposed	1511	477	749	BP-48068	LCSET-4954	TrueSeq.v1
GTEX-14BIL-0126-SM-5SI92	1	B1	8.2	Skin - Sun Exposed	1511	759	847	BP-47656	LCSET-5174	TrueSeq.v1
GTEX-14BMV-0126-SM-5S2PO	1	C1	8.1	Skin - Sun Exposed	1511	591	1159	BP-48647	LCSET-4988	TrueSeq.v1
GTEX-14C5O-0126-SM-5SI6N	1	C1	7.9	Skin - Sun Exposed	1511	654	511	BP-48651	LCSET-4995	TrueSeq.v1
GTEX-14DAQ-0126-SM-5S2MQ	0	C1	8	Skin - Sun Exposed	1511	448	628	BP-48576	LCSET-4988	TrueSeq.v1
GTEX-14DAR-2026-SM-5S2O3	0	B1	7.2	Skin - Sun Exposed	1511	250	619	BP-48576	LCSET-4988	TrueSeq.v1
GTEX-14E6C-0126-SM-5RQIP	1	C1	7.3	Skin - Sun Exposed	1511	981	410	BP-48576	LCSET-4955	TrueSeq.v1
GTEX-14E6E-1826-SM-5S2R7	0	B1	7.3	Skin - Sun Exposed	1511	395	381	BP-48651	LCSET-4988	TrueSeq.v1
GTEX-N7MS-0225-SM-4E3HO	1	C1	7.7	Skin - Sun Exposed	1511	1180	1563	BP-36182	LCSET-3416	TrueSeq.v1

SAMPID	SMATSSCR	SMCENTER	SMRIN	SMTSD	SMUBRID	SMTSISCH	SMTSPAX	SMNABTCH	SMGEBTCH	SMGEBTCHT
GTEX-NFK9-0226-SM-2AXU5	0	C1	9.6	Skin - Sun Exposed	1511	294	1569	BP-16993	GTExDonor1-20_Sept2011	Affymetrix Expression
GTEX-NFK9-0226-SM-2HMKQ	0	C1	9.6	Skin - Sun Exposed	1511	294	1569	BP-16993	LCSET-1156_2	TrueSeq.v1
GTEX-NPJ8-0126-SM-2QU56	0	D1	7.5	Skin - Sun Exposed	1511	311	1635	BP-17314	GTEX_AffyBatch5_March12	Affymetrix Expression
GTEX-NPJ8-0126-SM-2YUNR	0	D1	7.5	Skin - Sun Exposed	1511	311	1635	BP-17314	LCSET-1433	TrueSeq.v1
GTEX-O5YT-0126-SM-48TBW	0	B1	5.9	Skin - Sun Exposed	1511	242	1588	BP-17532	LCSET-3085	TrueSeq.v1
GTEX-O5YW-0126-SM-2D45C	0	B1	5.4	Skin - Sun Exposed	1511	194	1582	BP-17669	GTEx_96Affy3_Oct2011	Affymetrix Expression
GTEX-O5YW-0126-SM-3LK6D	0	B1	5.4	Skin - Sun Exposed	1511	194	1582	BP-17669	LCSET-2234	TrueSeq.v1
GTEX-OHPJ-0226-SM-2D45E	0	C1	5.5	Skin - Sun Exposed	1511	406	1560	BP-17749	GTEx_96Affy3_Oct2011	Affymetrix Expression
GTEX-OHPL-0126-SM-2AXUS	0	B1	7.1	Skin - Sun Exposed	1511	189	1563	BP-17822	GTExDonor1-20_Sept2011	Affymetrix Expression
GTEX-OHPL-0126-SM-2HMJ7	0	B1	7.1	Skin - Sun Exposed	1511	189	1563	BP-17822	LCSET-1156_1	TrueSeq.v1
GTEX-OHPM-0126-SM-2QU3F	0	B1	6.1	Skin - Sun Exposed	1511	149	1649	BP-20384	GTEX_AffyBatch5_March12	Affymetrix Expression
GTEX-OHPM-0126-SM-2YUN9	0	B1	6.1	Skin - Sun Exposed	1511	149	1649	BP-20384	LCSET-1433	TrueSeq.v1
GTEX-OIZF-0126-SM-2AXUE	0	B1	7.8	Skin - Sun Exposed	1511	215	256	BP-18080	GTExDonor1-20_Sept2011	Affymetrix Expression
GTEX-OIZG-0726-SM-2AXUL	0	C1	5.9	Skin - Sun Exposed	1511	730	241	BP-18122	GTExDonor1-20_Sept2011	Affymetrix Expression
GTEX-OIZG-0726-SM-2I5GS	0	C1	5.9	Skin - Sun Exposed	1511	730	241	BP-18122	LCSET-1168_5	TrueSeq.v1
GTEX-OIZG-0726-SM-33HBL	0	C1	5.9	Skin - Sun Exposed	1511	730	241	BP-18122	LCSET-1668	TrueSeq.v1
GTEX-OIZH-0126-SM-2D44Z	0	B1	6.6	Skin - Sun Exposed	1511	219	294	BP-18147	GTEx_96Affy3_Oct2011	Affymetrix Expression
GTEX-OIZH-0126-SM-2HMIS	0	B1	6.6	Skin - Sun Exposed	1511	219	294	BP-18147	LCSET-1156_1	TrueSeq.v1
GTEX-OOBK-0126-SM-2QU3Y	1	B1	7.2	Skin - Sun Exposed	1511	261	731	BP-20384	GTEX_AffyBatch5_March12	Affymetrix Expression
GTEX-OOBK-0126-SM-2YUND	1	B1	7.2	Skin - Sun Exposed	1511	261	731	BP-20384	LCSET-1433	TrueSeq.v1
GTEX-OXRK-0226-SM-2M485	1	C1	6.7	Skin - Sun Exposed	1511	280	240	BP-20384	GTEx_Batch4Affy_Jan2012	Affymetrix Expression
GTEX-OXRK-0226-SM-2YUN1	1	C1	6.7	Skin - Sun Exposed	1511	280	240	BP-20384	LCSET-1433	TrueSeq.v1
GTEX-OXRK-0226-SM-3NB2G	1	C1	6.7	Skin - Sun Exposed	1511	280	240	BP-20384	LCSET-2318	TrueSeq.v1
GTEX-OXRL-0126-SM-2M47I	1	B1	6	Skin - Sun Exposed	1511	312	852	BP-18390	GTEx_Batch4Affy_Jan2012	Affymetrix Expression
GTEX-OXRL-0126-SM-2YUMP	1	B1	6	Skin - Sun Exposed	1511	312	852	BP-18390	LCSET-1433	TrueSeq.v1

SAMPID	SMATSSCR	SMCENTER	SMRIN	SMTSD	SMUBRID	SMTSISCH	SMTSPAX	SMNABTCH	SMGEBTCH	SMGEBTCHT
GTEX-OXRN-0126-SM-48TDM	1	C1	8.6	Skin - Sun Exposed	1511	572	364	BP-29826	LCSET-3085	TrueSeq.v1
GTEX-OXRO-0126-SM-2M48E	1	D1	7	Skin - Sun Exposed	1511	835	1179	BP-18448	GTEx_Batch4Affy_Jan2012	Affymetrix Expression
GTEX-OXRO-0126-SM-2YUN4	1	D1	7	Skin - Sun Exposed	1511	835	1179	BP-18448	LCSET-1433	TrueSeq.v1
GTEX-OXRP-0126-SM-3NB32	1	C1	8.4	Skin - Sun Exposed	1511	1063	240	BP-29826	LCSET-2318	TrueSeq.v1
GTEX-OXRP-0126-SM-48TDI	1	C1	8.4	Skin - Sun Exposed	1511	1063	240	BP-29826	LCSET-3085	TrueSeq.v1
GTEX-P44G-0226-SM-2D44P	1	C1	6.3	Skin - Sun Exposed	1511	667	248	BP-18460	GTEx_96Affy3_Oct2011	Affymetrix Expression
GTEX-P44G-0226-SM-2I3F3	1	C1	6.3	Skin - Sun Exposed	1511	667	248	BP-18460	LCSET-1167_4	TrueSeq.v1
GTEX-P44H-0226-SM-2XCEU	1	C1	7	Skin - Sun Exposed	1511	458	461	BP-18616	LCSET-1562	TrueSeq.v1
GTEX-P44H-0226-SM-2XV67	1	C1	7	Skin - Sun Exposed	1511	458	461	BP-18616	GTEx_AffyBatch9_May12	Affymetrix Expression
GTEX-P4PP-0126-SM-2D442	1	B1	6.1	Skin - Sun Exposed	1511	196	854	BP-18662	GTEx_96Affy3_Oct2011	Affymetrix Expression
GTEX-P4PP-0126-SM-3LK69	1	B1	6.1	Skin - Sun Exposed	1511	196	854	BP-18662	LCSET-2234	TrueSeq.v1
GTEX-P4PQ-0126-SM-2M47B	1	B1	6	Skin - Sun Exposed	1511	235	1220	BP-18708	GTEx_Batch4Affy_Jan2012	Affymetrix Expression
GTEX-P4PQ-0126-SM-2S1NM	1	B1	6	Skin - Sun Exposed	1511	235	1220	BP-18708	LCSET-1464	TrueSeq.v1
GTEX-P4QR-0326-SM-2M47P	1	C1	6.2	Skin - Sun Exposed	1511	411	712	BP-18830	GTEx_Batch4Affy_Jan2012	Affymetrix Expression
GTEX-P4QR-0326-SM-2S1NU	1	C1	6.2	Skin - Sun Exposed	1511	411	712	BP-18830	LCSET-1464	TrueSeq.v1
GTEX-P4QR-0326-SM-5SI8P	1	C1	6.2	Skin - Sun Exposed	1511	411	712	BP-18830	LCSET-4995	TrueSeq.v1
GTEX-P4QT-0126-SM-2I3FL	1	B1	6.3	Skin - Sun Exposed	1511	212	369	BP-18729	LCSET-1167_3	TrueSeq.v1
GTEX-PLZ4-0126-SM-2QU4D	1	C1	6.1	Skin - Sun Exposed	1511	652	843	BP-19215	GTEX_AffyBatch5_March12	Affymetrix Expression
GTEX-PLZ4-0126-SM-2S1OG	1	C1	6.1	Skin - Sun Exposed	1511	652	843	BP-19215	LCSET-1464	TrueSeq.v1
GTEX-PLZ4-0126-SM-5SI8O	1	C1	6.1	Skin - Sun Exposed	1511	652	843	BP-19215	LCSET-4995	TrueSeq.v1
GTEX-PLZ5-2026-SM-2M48B	1	B1	6.7	Skin - Sun Exposed	1511	210	1146	BP-19182	GTEx_Batch4Affy_Jan2012	Affymetrix Expression
GTEX-PLZ5-2026-SM-2S1O4	1	B1	6.7	Skin - Sun Exposed	1511	210	1146	BP-19182	LCSET-1464	TrueSeq.v1
GTEX-PLZ6-1426-SM-2QU4V	1	B1	7	Skin - Sun Exposed	1511	231	1131	BP-19256	GTEX_AffyBatch5_March12	Affymetrix Expression
GTEX-PLZ6-1426-SM-2S1OQ	1	B1	7	Skin - Sun Exposed	1511	231	1131	BP-19256	LCSET-1464	TrueSeq.v1
GTEX-PLZ6-1426-SM-5S2TR	1	B1	7	Skin - Sun Exposed	1511	231	1131	BP-19256	LCSET-4996	TrueSeq.v1

SAMPID	SMATSSCR	SMCENTER	SMRIN	SMTSD	SMUBRID	SMTSISCH	SMTSPAX	SMNABTCH	SMGEBTCH	SMGEBTCHT
GTEX-POMQ-2226-SM-2I5FK	0	B1	7.1	Skin - Sun Exposed	1511	362	1308	BP-19351	LCSET-1168_5	TrueSeq.v1
GTEX-POMQ-2226-SM-2M48S	0	B1	7.1	Skin - Sun Exposed	1511	362	1308	BP-19351	GTEx_Batch4Affy_Jan2012	Affymetrix Expression
GTEX-PSDG-0226-SM-2I5FR	0	C1	7.4	Skin - Sun Exposed	1511	411	1337	BP-19411	LCSET-1168_5	TrueSeq.v1
GTEX-PSDG-0226-SM-2M48X	0	C1	7.4	Skin - Sun Exposed	1511	411	1337	BP-19411	GTEx_Batch4Affy_Jan2012	Affymetrix Expression
GTEX-PSDG-0226-SM-33HC1	0	C1	7.4	Skin - Sun Exposed	1511	411	1337	BP-19411	LCSET-1668	TrueSeq.v1
GTEX-PVOW-0126-SM-2XCFA	1	C1	7.6	Skin - Sun Exposed	1511	634	832	BP-19441	LCSET-1562	TrueSeq.v1
GTEX-PVOW-0126-SM-2XV68	1	C1	7.6	Skin - Sun Exposed	1511	634	832	BP-19441	GTEx_AffyBatch9_May12	Affymetrix Expression
GTEX-PW2O-1526-SM-2IZJD	1	B1	5.5	Skin - Sun Exposed	1511	278	1299	BP-19459	GTEx_Batch3Affy_Dec2011	Affymetrix Expression
GTEX-PWCY-1826-SM-2QU4P	1	B1	6.2	Skin - Sun Exposed	1511	281	747	BP-19461	GTEX_AffyBatch5_March12	Affymetrix Expression
GTEX-PWCY-1826-SM-2S1OK	1	B1	6.2	Skin - Sun Exposed	1511	281	747	BP-19461	LCSET-1464	TrueSeq.v1
GTEX-PWCY-1826-SM-5SI7U	1	B1	6.2	Skin - Sun Exposed	1511	281	747	BP-19461	LCSET-4995	TrueSeq.v1
GTEX-PWN1-0126-SM-2I3FK	1	B1	7.3	Skin - Sun Exposed	1511	212	1078	BP-19817	LCSET-1167_4	TrueSeq.v1
GTEX-PWN1-0126-SM-2IZIM	1	B1	7.3	Skin - Sun Exposed	1511	212	1078	BP-19817	GTEx_Batch3Affy_Dec2011	Affymetrix Expression
GTEX-PWO3-1526-SM-48TCM	1	D1	5.9	Skin - Sun Exposed	1511	1290	1187	BP-19825	LCSET-3085	TrueSeq.v1
GTEX-PX3G-0126-SM-2I3EN	1	B1	7.5	Skin - Sun Exposed	1511	247	373	BP-19882	LCSET-1167_3	TrueSeq.v1
GTEX-PX3G-0126-SM-2IZIC	1	B1	7.5	Skin - Sun Exposed	1511	247	373	BP-19882	GTEx_Batch3Affy_Dec2011	Affymetrix Expression
GTEX-Q2AG-0126-SM-2HMLB	1	C1	7.4	Skin - Sun Exposed	1511	696	1171	BP-19901	LCSET-1156_2	TrueSeq.v1
GTEX-Q2AG-0126-SM-2IZJX	1	C1	7.4	Skin - Sun Exposed	1511	696	1171	BP-19901	GTEx_Batch3Affy_Dec2011	Affymetrix Expression
GTEX-Q2AG-0126-SM-33HBV	1	C1	7.4	Skin - Sun Exposed	1511	696	1171	BP-19901	LCSET-1668	TrueSeq.v1
GTEX-Q2AH-1626-SM-2QU36	1	B1	6.9	Skin - Sun Exposed	1511	186	1120	BP-20000	GTEX_AffyBatch5_March12	Affymetrix Expression
GTEX-Q2AH-1626-SM-3GAF8	1	B1	6.9	Skin - Sun Exposed	1511	186	1120	BP-20000	LCSET-2015	TrueSeq.v1
GTEX-Q2AI-1326-SM-2QU41	1	B1	7.3	Skin - Sun Exposed	1511	185	558	BP-20043	GTEX_AffyBatch5_March12	Affymetrix Expression
GTEX-Q2AI-1326-SM-2S1PL	1	B1	7.3	Skin - Sun Exposed	1511	185	558	BP-20043	LCSET-1465	TrueSeq.v1
GTEX-Q734-1926-SM-2I5EV	0	B1	5.4	Skin - Sun Exposed	1511	130	1066	BP-20047	LCSET-1168_5	TrueSeq.v1
GTEX-Q734-1926-SM-2IZIV	0	B1	5.4	Skin - Sun Exposed	1511	130	1066	BP-20047	GTEx_Batch3Affy_Dec2011	Affymetrix Expression

SAMPID	SMATSSCR	SMCENTER	SMRIN	SMTSD	SMUBRID	SMTSISCH	SMTSPAX	SMNABTCH	SMGEBTCH	SMGEBTCHT
GTEX-QCQG-1726-SM-2QU57	1	B1	6.7	Skin - Sun Exposed	1511	298	989	BP-20114	GTEX_AffyBatch5_March12	Affymetrix Expression
GTEX-QCQG-1726-SM-3GIJ9	1	B1	6.7	Skin - Sun Exposed	1511	298	989	BP-20114	LCSET-2024	TrueSeq.v1
GTEX-QDT8-0126-SM-48TZ1	1	C1	5.9	Skin - Sun Exposed	1511	446	731	BP-20205	LCSET-3084	TrueSeq.v1
GTEX-QDVJ-1726-SM-2I5FX	1	C1	6.5	Skin - Sun Exposed	1511	535	393	BP-20260	LCSET-1168_6	TrueSeq.v1
GTEX-QDVJ-1726-SM-2M492	1	C1	6.5	Skin - Sun Exposed	1511	535	393	BP-20260	GTEx_Batch4Affy_Jan2012	Affymetrix Expression
GTEX-QDVN-2026-SM-2QU4I	1	B1	6	Skin - Sun Exposed	1511	260	771	BP-20294	GTEX_AffyBatch5_March12	Affymetrix Expression
GTEX-QDVN-2026-SM-3GAEP	1	B1	6	Skin - Sun Exposed	1511	260	771	BP-20294	LCSET-2015	TrueSeq.v1
GTEX-QEG4-0226-SM-2QU52	1	C1	7.3	Skin - Sun Exposed	1511	893	1099	BP-20319	GTEX_AffyBatch5_March12	Affymetrix Expression
GTEX-QEG4-0226-SM-2S1PY	1	C1	7.3	Skin - Sun Exposed	1511	893	1099	BP-20319	LCSET-1465	TrueSeq.v1
GTEX-QEG5-0226-SM-2I5GI	1	C1	7.3	Skin - Sun Exposed	1511	590	808	BP-20333	LCSET-1168_5	TrueSeq.v1
GTEX-QEG5-0226-SM-2M49K	1	C1	7.3	Skin - Sun Exposed	1511	590	808	BP-20333	GTEx_Batch4Affy_Jan2012	Affymetrix Expression
GTEX-QEL4-0226-SM-2TWAW	1	C1	7.5	Skin - Sun Exposed	1511	476	1387	BP-20412	GTEx_Affy_Batch7_Apr12	Affymetrix Expression
GTEX-QESD-1426-SM-2QU4L	1	B1	6.8	Skin - Sun Exposed	1511	326	1313	BP-20481	GTEX_AffyBatch5_March12	Affymetrix Expression
GTEX-QESD-1426-SM-2S1R9	1	B1	6.8	Skin - Sun Exposed	1511	326	1313	BP-20481	LCSET-1466	TrueSeq.v1
GTEX-QLQ7-1626-SM-2QU45	1	B1	6.6	Skin - Sun Exposed	1511	178	563	BP-20571	GTEX_AffyBatch5_March12	Affymetrix Expression
GTEX-QLQ7-1626-SM-2S1R8	1	B1	6.6	Skin - Sun Exposed	1511	178	563	BP-20571	LCSET-1466	TrueSeq.v1
GTEX-QLQW-1126-SM-2S1Q8	1	B1	7.3	Skin - Sun Exposed	1511	159	1087	BP-20691	LCSET-1466	TrueSeq.v1
GTEX-QLQW-1126-SM-2TO72	1	B1	7.3	Skin - Sun Exposed	1511	159	1087	BP-20691	GTEX_Affy_Batch6_Apr12	Affymetrix Expression
GTEX-QV44-1926-SM-2S1RF	1	B1	6.4	Skin - Sun Exposed	1511	196	954	BP-20869	LCSET-1466	TrueSeq.v1
GTEX-QV44-1926-SM-2TO4T	1	B1	6.4	Skin - Sun Exposed	1511	196	954	BP-20869	GTEX_Affy_Batch6_Apr12	Affymetrix Expression
GTEX-QVJO-1626-SM-2S1QW	1	D1	6.5	Skin - Sun Exposed	1511	1222	1052	BP-20981	LCSET-1466	TrueSeq.v1
GTEX-QVJO-1626-SM-2TO5E	1	D1	6.5	Skin - Sun Exposed	1511	1222	1052	BP-20981	GTEX_Affy_Batch6_Apr12	Affymetrix Expression
GTEX-R3RS-0126-SM-2TO5G	1	C1	6.6	Skin - Sun Exposed	1511	1151	755	BP-21198	GTEX_Affy_Batch6_Apr12	Affymetrix Expression
GTEX-R3RS-0126-SM-3GIJL	1	C1	6.6	Skin - Sun Exposed	1511	1151	755	BP-21198	LCSET-2024	TrueSeq.v1
GTEX-R55D-0226-SM-2TWBG	0	C1	6.1	Skin - Sun Exposed	1511	360	1166	BP-21287	GTEx_Affy_Batch7_Apr12	Affymetrix Expression

SAMPID	SMATSSCR	SMCENTER	SMRIN	SMTSD	SMUBRID	SMTSISCH	SMTSPAX	SMNABTCH	SMGEBTCH	SMGEBTCHT
GTEX-R55E-0126-SM-2TC5Y	1	C1	7.6	Skin - Sun Exposed	1511	924	1258	BP-21443	LCSET-1477	TrueSeq.v1
GTEX-R55E-0126-SM-2TO6U	1	C1	7.6	Skin - Sun Exposed	1511	924	1258	BP-21443	GTEX_Affy_Batch6_Apr12	Affymetrix Expression
GTEX-R55F-0126-SM-48FCK	1	D1	5.8	Skin - Sun Exposed	1511	1287	1129	BP-21404	LCSET-3057	TrueSeq.v1
GTEX-R55G-2526-SM-2TC6D	0	B1	7	Skin - Sun Exposed	1511	189	729	BP-21547	LCSET-1477	TrueSeq.v1
GTEX-RM2N-1426-SM-2TF4H	0	B1	6.5	Skin - Sun Exposed	1511	227	1020	BP-21576	LCSET-1479	TrueSeq.v1
GTEX-RM2N-1426-SM-2TO53	0	B1	6.5	Skin - Sun Exposed	1511	227	1020	BP-21576	GTEX_Affy_Batch6_Apr12	Affymetrix Expression
GTEX-RN64-0126-SM-2TC68	1	C1	7.2	Skin - Sun Exposed	1511	676	1113	BP-21626	LCSET-1477	TrueSeq.v1
GTEX-RN64-0126-SM-2TO55	1	C1	7.2	Skin - Sun Exposed	1511	676	1113	BP-21626	GTEX_Affy_Batch6_Apr12	Affymetrix Expression
GTEX-RNOR-0126-SM-2TF57	1	C1	7.4	Skin - Sun Exposed	1511	827	636	BP-21668	LCSET-1479	TrueSeq.v1
GTEX-RNOR-0126-SM-2TWAZ	1	C1	7.4	Skin - Sun Exposed	1511	827	636	BP-21668	GTEx_Affy_Batch7_Apr12	Affymetrix Expression
GTEX-RTLS-0126-SM-447CA	1	C1	5.9	Skin - Sun Exposed	1511	632	783	BP-21669	LCSET-2912	TrueSeq.v1
GTEX-RU72-0926-SM-2TF6B	0	C1	6.8	Skin - Sun Exposed	1511	457	1399	BP-21837	LCSET-1480	TrueSeq.v1
GTEX-RU72-0926-SM-2TWAP	0	C1	6.8	Skin - Sun Exposed	1511	457	1399	BP-21837	GTEx_Affy_Batch7_Apr12	Affymetrix Expression
GTEX-RUSQ-1526-SM-2TF62	0	B1	6.2	Skin - Sun Exposed	1511	288	1178	BP-21934	LCSET-1480	TrueSeq.v1
GTEX-RUSQ-1526-SM-2TWBQ	0	B1	6.2	Skin - Sun Exposed	1511	288	1178	BP-21934	GTEx_Affy_Batch7_Apr12	Affymetrix Expression
GTEX-RVPU-2226-SM-2XCAQ	1	C1	7.2	Skin - Sun Exposed	1511	1431	1327	BP-22343	LCSET-1559	TrueSeq.v1
GTEX-RVPU-2226-SM-2XU9A	1	C1	7.2	Skin - Sun Exposed	1511	1431	1327	BP-22343	GTEx_AffyBatch8_May12	Affymetrix Expression
GTEX-RWS6-2026-SM-2XCB5	1	B1	6.6	Skin - Sun Exposed	1511	235	895	BP-22521	LCSET-1559	TrueSeq.v1
GTEX-RWS6-2026-SM-2XU8X	1	B1	6.6	Skin - Sun Exposed	1511	235	895	BP-22521	GTEx_AffyBatch8_May12	Affymetrix Expression
GTEX-RWSA-0126-SM-2XCBB	0	C1	8	Skin - Sun Exposed	1511	1284	1425	BP-22521	LCSET-1559	TrueSeq.v1
GTEX-RWSA-0126-SM-2XU9X	0	C1	8	Skin - Sun Exposed	1511	1284	1425	BP-22521	GTEx_AffyBatch8_May12	Affymetrix Expression
GTEX-S32W-2126-SM-2XCB1	1	B1	7.9	Skin - Sun Exposed	1511	393	842	BP-22722	LCSET-1559	TrueSeq.v1
GTEX-S32W-2126-SM-2XU8S	1	B1	7.9	Skin - Sun Exposed	1511	393	842	BP-22722	GTEx_AffyBatch8_May12	Affymetrix Expression
GTEX-S341-1726-SM-2XU8C	0	B1	6.1	Skin - Sun Exposed	1511	224	985	BP-22843	GTEx_AffyBatch8_May12	Affymetrix Expression
GTEX-S341-1726-SM-3K2AK	0	B1	6.1	Skin - Sun Exposed	1511	224	985	BP-22843	LCSET-2147	TrueSeq.v1

SAMPID	SMATSSCR	SMCENTER	SMRIN	SMTSD	SMUBRID	SMTSISCH	SMTSPAX	SMNABTCH	SMGEBTCH	SMGEBTCHT
GTEX-S3XE-1726-SM-2XU9F	0	B1	7.8	Skin - Sun Exposed	1511	207	375	BP-22893	GTEx_AffyBatch8_May12	Affymetrix Expression
GTEX-S3XE-1726-SM-3K2AM	0	B1	7.8	Skin - Sun Exposed	1511	207	375	BP-22893	LCSET-2147	TrueSeq.v1
GTEX-S4UY-0126-SM-2XU9R	0	C1	7.4	Skin - Sun Exposed	1511	323	750	BP-22873	GTEx_AffyBatch8_May12	Affymetrix Expression
GTEX-S4UY-0126-SM-3K2BB	0	C1	7.4	Skin - Sun Exposed	1511	323	750	BP-22873	LCSET-2147	TrueSeq.v1
GTEX-S7PM-0126-SM-4AD6S	1	C1	7.2	Skin - Sun Exposed	1511	1266	1177	BP-23146	LCSET-3098	TrueSeq.v1
GTEX-S7SE-0126-SM-2XCD5	1	C1	6.8	Skin - Sun Exposed	1511	936	1233	BP-23169	LCSET-1561	TrueSeq.v1
GTEX-S7SE-0126-SM-2XV4Z	1	C1	6.8	Skin - Sun Exposed	1511	936	1233	BP-23169	GTEx_AffyBatch9_May12	Affymetrix Expression
GTEX-S7SF-1626-SM-2XU9K	1	B1	6.8	Skin - Sun Exposed	1511	339	754	BP-23171	GTEx_AffyBatch8_May12	Affymetrix Expression
GTEX-S7SF-1626-SM-3K2AY	1	B1	6.8	Skin - Sun Exposed	1511	339	754	BP-23171	LCSET-2147	TrueSeq.v1
GTEX-S95S-1226-SM-4GICG	0	B1	5.8	Skin - Sun Exposed	1511	275	1011	BP-23283	LCSET-3479	TrueSeq.v1
GTEX-SIU7-1726-SM-2XCDZ	1	B1	7.1	Skin - Sun Exposed	1511	168	979	BP-23467	LCSET-1561	TrueSeq.v1
GTEX-SIU7-1726-SM-2XV4K	1	B1	7.1	Skin - Sun Exposed	1511	168	979	BP-23467	GTEx_AffyBatch9_May12	Affymetrix Expression
GTEX-SIU8-0126-SM-2XCDT	1	C1	6.2	Skin - Sun Exposed	1511	1231	1063	BP-23696	LCSET-1561	TrueSeq.v1
GTEX-SIU8-0126-SM-2XV58	1	C1	6.2	Skin - Sun Exposed	1511	1231	1063	BP-23696	GTEx_AffyBatch9_May12	Affymetrix Expression
GTEX-SJXC-0126-SM-2XCFF	1	C1	7.4	Skin - Sun Exposed	1511	973	753	BP-23696	LCSET-1562	TrueSeq.v1
GTEX-SJXC-0126-SM-2XV4I	1	C1	7.4	Skin - Sun Exposed	1511	973	753	BP-23696	GTEx_AffyBatch9_May12	Affymetrix Expression
GTEX-SN8G-0126-SM-32PLI	1	C1	7.2	Skin - Sun Exposed	1511	1034	1043	BP-23791	LCSET-1666	TrueSeq.v1
GTEX-SNMC-1226-SM-2XCFP	1	B1	7.7	Skin - Sun Exposed	1511	269	1009	BP-23820	LCSET-1562	TrueSeq.v1
GTEX-SNMC-1226-SM-2XV54	1	B1	7.7	Skin - Sun Exposed	1511	269	1009	BP-23820	GTEx_AffyBatch9_May12	Affymetrix Expression
GTEX-SSA3-0126-SM-32QPU	0	B1	7.1	Skin - Sun Exposed	1511	40	1310	BP-24519	LCSET-1667	TrueSeq.v1
GTEX-SUCS-1926-SM-32PM3	1	B1	6.9	Skin - Sun Exposed	1511	209	1131	BP-24618	LCSET-1666	TrueSeq.v1
GTEX-T2IS-0126-SM-4DM6O	0	C1	7.8	Skin - Sun Exposed	1511	1076	1357	BP-24455	LCSET-3205	TrueSeq.v1
GTEX-T2YK-0526-SM-32QPJ	1	C1	7	Skin - Sun Exposed	1511	859	1152	BP-24618	LCSET-1667	TrueSeq.v1
GTEX-T5JC-0426-SM-32PLO	1	C1	8.2	Skin - Sun Exposed	1511	663	828	BP-24678	LCSET-1666	TrueSeq.v1
GTEX-T5JC-0426-SM-5S2SL	1	C1	8.2	Skin - Sun Exposed	1511		828	BP-24678	LCSET-4990	

SAMPID	SMATSSCR	SMCENTER	SMRIN	SMTSD	SMUBRID	SMTSISCH	SMTSPAX	SMNABTCH	SMGEBTCH	SMGEBTCHT
GTEX-T5JW-1626-SM-3GADZ	0	B1	6.8	Skin - Sun Exposed	1511	235	891	BP-26092	LCSET-2014	TrueSeq.v1
GTEX-T6MN-0126-SM-32PLP	1	C1	7.8	Skin - Sun Exposed	1511	689	899	BP-24678	LCSET-1666	TrueSeq.v1
GTEX-T6MN-0126-SM-5S2TD	1	C1	7.8	Skin - Sun Exposed	1511		899	BP-24678	LCSET-4990	
GTEX-T6MO-1626-SM-32QOM	0	B1	7	Skin - Sun Exposed	1511	182	730	BP-24889	LCSET-1667	TrueSeq.v1
GTEX-T8EM-1026-SM-3DB7M	0	C1	7.3	Skin - Sun Exposed	1511	378	1043	BP-24889	LCSET-1897	TrueSeq.v1
GTEX-TKQ1-1026-SM-4GICL	0	B1	7.8	Skin - Sun Exposed	1511	186	735	BP-29546	LCSET-3479	TrueSeq.v1
GTEX-TMKS-0126-SM-3DB7S	0	B1	7.2	Skin - Sun Exposed	1511	37	1051	BP-25490	LCSET-1897	TrueSeq.v1
GTEX-TML8-1926-SM-32QOS	1	B1	7.2	Skin - Sun Exposed	1511	256	972	BP-25076	LCSET-1667	TrueSeq.v1
GTEX-TMMY-0226-SM-33HBA	0	C1	6.8	Skin - Sun Exposed	1511	359	1250	BP-25072	LCSET-1668	TrueSeq.v1
GTEX-TMZS-0126-SM-3DB9Q	0	B1	6.4	Skin - Sun Exposed	1511	49	1082	BP-25493	LCSET-1898	TrueSeq.v1
GTEX-TSE9-0126-SM-3DB83	1	C1	8	Skin - Sun Exposed	1511	1023	1424	BP-25188	LCSET-1897	TrueSeq.v1
GTEX-U3ZH-2026-SM-3DB78	0	B1	8	Skin - Sun Exposed	1511	248	1147	BP-25326	LCSET-1897	TrueSeq.v1
GTEX-U3ZM-1526-SM-3DB9D	0	B1	6.3	Skin - Sun Exposed	1511	249	731	BP-25562	LCSET-1898	TrueSeq.v1
GTEX-U3ZN-2326-SM-3DB7W	0	B1	6.7	Skin - Sun Exposed	1511	176	956	BP-25495	LCSET-1897	TrueSeq.v1
GTEX-U412-0426-SM-3DB9O	1	C1	6.8	Skin - Sun Exposed	1511	1359	1381	BP-25560	LCSET-1898	TrueSeq.v1
GTEX-U4B1-1826-SM-4DXSU	0	B1	6.9	Skin - Sun Exposed	1511	245	1171	BP-25601	LCSET-3206	TrueSeq.v1
GTEX-U8T8-0126-SM-3DB94	1	C1	6.5	Skin - Sun Exposed	1511	975	863	BP-25630	LCSET-1898	TrueSeq.v1
GTEX-U8XE-0326-SM-3DB8P	0	C1	7.1	Skin - Sun Exposed	1511	358	900	BP-25740	LCSET-1898	TrueSeq.v1
GTEX-UJHI-1526-SM-3DB99	1	B1	6.6	Skin - Sun Exposed	1511	287	916	BP-25965	LCSET-1898	TrueSeq.v1
GTEX-UPIC-1626-SM-4IHKT	0	B1	5.8	Skin - Sun Exposed	1511	247	634	BP-30376	LCSET-3596	TrueSeq.v1
GTEX-UPJH-0326-SM-3GADU	1	C1	7.5	Skin - Sun Exposed	1511	799	1291	BP-26354	LCSET-2014	TrueSeq.v1
GTEX-UPK5-0726-SM-4IHL7	1	C1	5.8	Skin - Sun Exposed	1511	983	1071	BP-26451	LCSET-3596	TrueSeq.v1
GTEX-V1D1-2226-SM-3NMAX	0	B1	7.1	Skin - Sun Exposed	1511	472	1043	BP-26868	LCSET-2351	TrueSeq.v1
GTEX-VJWN-0126-SM-3GIK7	2	C1	7.5	Skin - Sun Exposed	1511	1045	1007	BP-26938	LCSET-2024	TrueSeq.v1
GTEX-VJYA-1126-SM-3GIJU	0	B1	7.2	Skin - Sun Exposed	1511	257	1062	BP-26934	LCSET-2024	TrueSeq.v1

SAMPID	SMATSSCR	SMCENTER	SMRIN	SMTSD	SMUBRID	SMTSISCH	SMTSPAX	SMNABTCH	SMGEBTCH	SMGEBTCHT
GTEX-VUSG-2526-SM-4KL1V	0	B1	7.2	Skin - Sun Exposed	1511	289	474	BP-30217	LCSET-3626	TrueSeq.v1
GTEX-VUSG-2526-SM-5S2S9	0	B1	7.2	Skin - Sun Exposed	1511		474	BP-30217	LCSET-4990	
GTEX-W5WG-1826-SM-4KL2Y	0	B1	6.6	Skin - Sun Exposed	1511	266	1082	BP-27175	LCSET-3626	TrueSeq.v1
GTEX-WEY5-1826-SM-5CHRT_rep1	1	B1	5.4	Skin - Sun Exposed	1511		672	BP-27394		
GTEX-WEY5-1826-SM-5CHRT_rep2	1	B1	5.4	Skin - Sun Exposed	1511		672	BP-27394		
GTEX-WFG8-2226-SM-3GIL9	0	B1	6.2	Skin - Sun Exposed	1511	333	680	BP-27446	LCSET-2025	TrueSeq.v1
GTEX-WFG8-2226-SM-5CHSS_rep1	0	B1	6.2	Skin - Sun Exposed	1511		680	BP-27446		
GTEX-WFON-2126-SM-3LK7O	0	B1	7.7	Skin - Sun Exposed	1511	219	446	BP-27491	LCSET-2234	TrueSeq.v1
GTEX-WFON-2126-SM-5CHR4_rep1	0	B1	7.7	Skin - Sun Exposed	1511		446	BP-27491		
GTEX-WFON-2126-SM-5S2T9	0	B1	7.7	Skin - Sun Exposed	1511		446	BP-27491	LCSET-5404	
GTEX-WH7G-2326-SM-3NMBC	1	B1	6.2	Skin - Sun Exposed	1511	376	879	BP-27853	LCSET-2385	TrueSeq.v1
GTEX-WI4N-1026-SM-3LK7N	0	C1	7	Skin - Sun Exposed	1511	688	846	BP-27941	LCSET-2234	TrueSeq.v1
GTEX-WOFL-0126-SM-3MJG2	1	C1	7.4	Skin - Sun Exposed	1511	576	1100	BP-28093	LCSET-2269	TrueSeq.v1
GTEX-WQUQ-0426-SM-3MJFU	1	C1	7.1	Skin - Sun Exposed	1511	404	1287	BP-28275	LCSET-2269	TrueSeq.v1
GTEX-WRHK-1426-SM-3MJF9	0	B1	7	Skin - Sun Exposed	1511	299	375	BP-28509	LCSET-2269	TrueSeq.v1
GTEX-WVJS-0126-SM-4MVOT	1	C1	6.2	Skin - Sun Exposed	1511	726	1434	BP-36182	LCSET-3652	TrueSeq.v1
GTEX-WXYG-2326-SM-4E3I6	0	B1	7.8	Skin - Sun Exposed	1511	213	591	BP-36063	LCSET-3416	TrueSeq.v1
GTEX-WY7C-2326-SM-3NB2U	1	B1	7.1	Skin - Sun Exposed	1511	235	848	BP-29478	LCSET-2318	TrueSeq.v1
GTEX-WYBS-0126-SM-3PYKA	2	C1		Skin - Sun Exposed	1511	853	610	BP-29327	GTEx_batch9_EX_001	Illumina Human Exome SNP Array
GTEX-WYBS-0126-SM-3PZ7C	2	C1		Skin - Sun Exposed	1511	853	610	BP-29327	GTEx_batch9_OM5_001	Illumina OMNI SNP Array
GTEX-WYBS-0126-SM-4SVQC	2	C1		Skin - Sun Exposed	1511	853	610	BP-29327	LCSET-4029	Standard Exome Sequencing v3
GTEX-WYJK-0126-SM-3NMAB	0	C1	6.9	Skin - Sun Exposed	1511	704	750	BP-29433	LCSET-2351	TrueSeq.v1
GTEX-WYVS-2126-SM-3NMA3	0	B1	7.7	Skin - Sun Exposed	1511	311	798	BP-29831	LCSET-2351	TrueSeq.v1
GTEX-WZTO-0126-SM-3NM95	0	C1	7.7	Skin - Sun Exposed	1511	478	838	BP-29770	LCSET-2351	TrueSeq.v1
GTEX-X261-0126-SM-3NMD6	1	B1	8	Skin - Sun Exposed	1511	741	1067	BP-29824	LCSET-2385	TrueSeq.v1

SAMPID	SMATSSCR	SMCENTER	SMRIN	SMTSD	SMUBRID	SMTSISCH	SMTSPAX	SMNABTCH	SMGEBTCH	SMGEBTCHT
GTEX-X4EO-0326-SM-3P5YO	2	C1	7.8	Skin - Sun Exposed	1511	779	1338	BP-30679	LCSET-2386	TrueSeq.v1
GTEX-X4EP-0126-SM-3P5YV	0	C1	6	Skin - Sun Exposed	1511	1104	1409	BP-30276	LCSET-2386	TrueSeq.v1
GTEX-X4XX-0126-SM-3NMC2	0	C1	6.5	Skin - Sun Exposed	1511	287	1437	BP-30738	LCSET-2385	TrueSeq.v1
GTEX-X4XY-0226-SM-4E3IZ	1	C1	8.5	Skin - Sun Exposed	1511	609	1122	BP-36201	LCSET-3416	TrueSeq.v1
GTEX-X585-0226-SM-4QAS2	1	C1	5.7	Skin - Sun Exposed	1511	780	1127	BP-31273	LCSET-3737	TrueSeq.v1
GTEX-X5EB-2226-SM-46MW4	0	B1	7.4	Skin - Sun Exposed	1511	274	947	BP-31273	LCSET-2933	TrueSeq.v1
GTEX-X62O-0126-SM-4E3JN	1	C1	6.4	Skin - Sun Exposed	1511	851	793	BP-36063	LCSET-3416	TrueSeq.v1
GTEX-X638-0126-SM-47JZ8	0	B1	7.4	Skin - Sun Exposed	1511	42	1048	BP-36063	LCSET-2974	TrueSeq.v1
GTEX-X88G-0126-SM-47JZ3	0	B1	6.6	Skin - Sun Exposed	1511	130	1147	BP-32413	LCSET-2974	TrueSeq.v1
GTEX-X8HC-0126-SM-4E3JW	1	C1	6.6	Skin - Sun Exposed	1511	749	921	BP-36259	LCSET-3416	TrueSeq.v1
GTEX-XAJ8-0826-SM-47JY6	0	B1	8	Skin - Sun Exposed	1511	168	1093	BP-31549	LCSET-2974	TrueSeq.v1
GTEX-XBED-2226-SM-47JYQ	1	B1	8.5	Skin - Sun Exposed	1511	211	378	BP-31585	LCSET-2974	TrueSeq.v1
GTEX-XBEW-0626-SM-4QASP	0	C1	8.9	Skin - Sun Exposed	1511	439	1409	BP-36349	LCSET-3737	TrueSeq.v1
GTEX-XMD2-0126-SM-4YCDU	1	C1	7.7	Skin - Sun Exposed	1511	1339	1174	BP-36411	LCSET-4197	TrueSeq.v1
GTEX-XOTO-0126-SM-4B66N	0	C1	8.1	Skin - Sun Exposed	1511	592	1238	BP-33606	LCSET-3141	TrueSeq.v1
GTEX-XPVG-2626-SM-4B669	1	B1	7.4	Skin - Sun Exposed	1511	221	914	BP-34001	LCSET-3141	TrueSeq.v1
GTEX-XQ3S-1526-SM-4BOOC	1	C1	7.1	Skin - Sun Exposed	1511	894	1115	BP-34117	LCSET-3145	TrueSeq.v1
GTEX-XQ8I-0426-SM-4BOPO	1	C1	7.7	Skin - Sun Exposed	1511	412	1103	BP-33923	LCSET-3145	TrueSeq.v1
GTEX-XUJ4-2426-SM-4BOO3	0	B1	6.2	Skin - Sun Exposed	1511	197	503	BP-34317	LCSET-3145	TrueSeq.v1
GTEX-XUW1-0426-SM-4BOOT	0	C1	7.6	Skin - Sun Exposed	1511	337	997	BP-34129	LCSET-3145	TrueSeq.v1
GTEX-XUYS-0126-SM-47JWZ	0	B1	8.8	Skin - Sun Exposed	1511	27	1230	BP-34929	LCSET-2974	TrueSeq.v1
GTEX-XUZC-1726-SM-4BRWS	0	B1	6.3	Skin - Sun Exposed	1511	163	466	BP-34543	LCSET-3152	TrueSeq.v1
GTEX-XV7Q-2526-SM-4BRV9	0	B1	6.7	Skin - Sun Exposed	1511	146	370	BP-34738	LCSET-3152	TrueSeq.v1
GTEX-XXEK-2226-SM-4BRUM	0	B1	6.2	Skin - Sun Exposed	1511	215	1132	BP-34799	LCSET-3152	TrueSeq.v1
GTEX-XXEK-2226-SM-5S2SA	0	B1	6.2	Skin - Sun Exposed	1511		1132	BP-34799	LCSET-4990	

SAMPID	SMATSSCR	SMCENTER	SMRIN	SMTSD	SMUBRID	SMTSISCH	SMTSPAX	SMNABTCH	SMGEBTCH	SMGEBTCHT
GTEX-XYKS-2126-SM-4E3IB	1	B1	8.4	Skin - Sun Exposed	1511	218	1073	BP-34929	LCSET-3416	TrueSeq.v1
GTEX-Y111-0126-SM-4SOIV	2	B1	7.3	Skin - Sun Exposed	1511	1399	1157	BP-34830	LCSET-3995	TrueSeq.v1
GTEX-Y114-2426-SM-4TT8A	0	B1	7.6	Skin - Sun Exposed	1511	228	466	BP-35758	LCSET-4016	TrueSeq.v1
GTEX-Y3I4-2126-SM-4TT7C	0	B1	7.7	Skin - Sun Exposed	1511	179	393	BP-41604	LCSET-4016	TrueSeq.v1
GTEX-Y3IK-2426-SM-4WWDU	0	B1	8.3	Skin - Sun Exposed	1511	174	380	BP-35836	LCSET-4180	TrueSeq.v1
GTEX-Y5LM-1926-SM-5RQJH	1	B1	7	Skin - Sun Exposed	1511	187	964	BP-48564	LCSET-4955	TrueSeq.v1
GTEX-Y5V5-2326-SM-4V6GA	0	B1	8.4	Skin - Sun Exposed	1511	266	419	BP-41604	LCSET-4035	TrueSeq.v1
GTEX-Y5V6-2426-SM-4VDSB	0	B1	7.6	Skin - Sun Exposed	1511	212	1261	BP-42185	LCSET-4040	TrueSeq.v1
GTEX-Y8DK-0126-SM-4TT3L	0	B1	8.2	Skin - Sun Exposed	1511	1100	581	BP-41767	LCSET-4014	TrueSeq.v1
GTEX-Y8E4-0626-SM-4WWDL	1	C1	8	Skin - Sun Exposed	1511	376	629	BP-41604	LCSET-4180	TrueSeq.v1
GTEX-Y8LW-1826-SM-5S2MV	0	B1	6.3	Skin - Sun Exposed	1511	341	1027	BP-48564	LCSET-4988	TrueSeq.v1
GTEX-YB5E-2026-SM-5IFIS	0	B1	7.5	Skin - Sun Exposed	1511	261	856	BP-45247	LCSET-4830	TrueSeq.v1
GTEX-YB5K-2126-SM-4WWDJ	1	B1	7.2	Skin - Sun Exposed	1511	284	1221	BP-41767	LCSET-4180	TrueSeq.v1
GTEX-YEC4-2126-SM-5IFJH	0	B1	6.6	Skin - Sun Exposed	1511	253	1008	BP-45247	LCSET-4830	TrueSeq.v1
GTEX-YF7O-2326-101833-SM-5CVN9		B1	6	Skin - Sun Exposed				BP-45390	LCSET-4633	TrueSeq.v1
GTEX-YFC4-0126-101855-SM-5CVLZ		C1	7.4	Skin - Sun Exposed				BP-45390	LCSET-4633	TrueSeq.v1
GTEX-YFC4-0126-SM-5CVLY	0	C1	6.8	Skin - Sun Exposed	1511	688	1403	BP-45390	LCSET-4633	TrueSeq.v1
GTEX-YJ89-0126-SM-4TT3X	1	C1	7.9	Skin - Sun Exposed	1511	1135	1439	BP-42128	LCSET-4014	TrueSeq.v1
GTEX-YJ8A-0426-SM-5IFID	0	B1	7.8	Skin - Sun Exposed	1511	211	479	BP-45395	LCSET-4830	TrueSeq.v1
GTEX-Z93S-0126-SM-5HL7M	0	C1	7.1	Skin - Sun Exposed	1511	1038	1261	BP-45395	LCSET-4807	TrueSeq.v1
GTEX-Z93T-0126-SM-5HL5N	0	B1	6.9	Skin - Sun Exposed	1511	17	868	BP-45247	LCSET-4806	TrueSeq.v1
GTEX-Z9EW-1626-SM-5CVMN	0	B1	7.8	Skin - Sun Exposed	1511	239	1058	BP-45390	LCSET-4633	TrueSeq.v1
GTEX-ZAB4-0226-SM-5N9F5	1	C1	5.7	Skin - Sun Exposed	1511	465	535	BP-45390	LCSET-4905	TrueSeq.v1
GTEX-ZAB5-1826-SM-5HL7C	0	B1	6.6	Skin - Sun Exposed	1511	223	621	BP-45395	LCSET-4807	TrueSeq.v1
GTEX-ZAJG-0126-SM-5HL94	0	C1	6.5	Skin - Sun Exposed	1511	724	625	BP-45395	LCSET-4807	TrueSeq.v1

SAMPID	SMATSSCR	SMCENTER	SMRIN	SMTSD	SMUBRID	SMTSISCH	SMTSPAX	SMNABTCH	SMGEBTCH	SMGEBTCHT
GTEX-ZAK1-0126-SM-5IJD3	0	C1	7.3	Skin - Sun Exposed	1511	710	1296	BP-45485	LCSET-4814	TrueSeq.v1
GTEX-ZC5H-0226-SM-4WAY9	0	B1	6.6	Skin - Sun Exposed	1511	212	491	BP-42024	LCSET-4162	TrueSeq.v1
GTEX-ZDTS-0126-SM-4WAY5	0	C1	7.5	Skin - Sun Exposed	1511	628	563	BP-42528	LCSET-4162	TrueSeq.v1
GTEX-ZDTT-2726-SM-4WKF8	0	B1	6.7	Skin - Sun Exposed	1511	183	360	BP-42588	LCSET-4170	TrueSeq.v1
GTEX-ZDXO-3026-SM-5J1N5	0	B1	6.1	Skin - Sun Exposed	1511	1073	1152	BP-45485	LCSET-4816	TrueSeq.v1
GTEX-ZDYS-2026-SM-5HL5L	0	C1	7.6	Skin - Sun Exposed	1511	205	398	BP-45395	LCSET-4806	TrueSeq.v1
GTEX-ZE7O-0126-SM-57WC1	1	B1	7.2	Skin - Sun Exposed	1511	1065	1116	BP-42826	LCSET-4415	TrueSeq.v1
GTEX-ZE9C-2726-SM-57WB5	0	C1	7.3	Skin - Sun Exposed	1511	682	1279	BP-42743	LCSET-4788	TrueSeq.v1
GTEX-ZEX8-2426-SM-4WKG4	1	B1	5.7	Skin - Sun Exposed	1511	195	878	BP-42869	LCSET-4170	TrueSeq.v1
GTEX-ZF28-0126-SM-4WKGK	2	C1	5.8	Skin - Sun Exposed	1511	463	613	BP-42869	LCSET-4170	TrueSeq.v1
GTEX-ZF2S-2326-SM-4WWCE	1	B1	8.5	Skin - Sun Exposed	1511	184	839	BP-43141	LCSET-4179	TrueSeq.v1
GTEX-ZGAY-0526-SM-4WWBB	0	C1	8	Skin - Sun Exposed	1511	229	1358	BP-43072	LCSET-4179	TrueSeq.v1
GTEX-ZP4G-2426-SM-57WEY	0	B1	7.2	Skin - Sun Exposed	1511	270	1032	BP-43308	LCSET-4416	TrueSeq.v1
GTEX-ZPIC-1726-SM-57WF2	0	B1	7.5	Skin - Sun Exposed	1511	162	1180	BP-43231	LCSET-4416	TrueSeq.v1
GTEX-ZQG8-1626-SM-5HL6H	2	B1	7.7	Skin - Sun Exposed	1511	180	1104	BP-45268	LCSET-4806	TrueSeq.v1
GTEX-ZQUD-1426-SM-57WER	0	B1	6.5	Skin - Sun Exposed	1511	221	1047	BP-43231	LCSET-4416	TrueSeq.v1
GTEX-ZT9W-2426-SM-57WFJ	0	B1	7.3	Skin - Sun Exposed	1511	180	726	BP-43441	LCSET-4416	TrueSeq.v1
GTEX-ZTPG-0326-SM-5O9AX	0	B1	7.3	Skin - Sun Exposed	1511	208	726	BP-43639	LCSET-4909	TrueSeq.v1
GTEX-ZTSS-1926-SM-57WEW	0	B1	7	Skin - Sun Exposed	1511	244	1234	BP-43441	LCSET-4416	TrueSeq.v1
GTEX-ZTX8-1426-SM-5DUVO	0	B1	7.5	Skin - Sun Exposed	1511	184	831	BP-43308	LCSET-4634	TrueSeq.v1
GTEX-ZU9S-0226-SM-5E441	1	C1	7.4	Skin - Sun Exposed	1511	493	1185	BP-43308	LCSET-4635	TrueSeq.v1
GTEX-ZUA1-0126-SM-5GU76	0	C1	6.1	Skin - Sun Exposed	1511	865	754	BP-43823	LCSET-4803	TrueSeq.v1
GTEX-ZV68-0126-SM-59HKS	0	B1	8	Skin - Sun Exposed	1511	1237	1033	BP-43231	LCSET-4419	TrueSeq.v1
GTEX-ZV6S-2326-SM-4YCEY	1	B1	7.1	Skin - Sun Exposed	1511	433	503	BP-43375	LCSET-4197	TrueSeq.v1
GTEX-ZVE2-0226-SM-59HJB	1	C1	7.7	Skin - Sun Exposed	1511	521	838	BP-43441	LCSET-4419	TrueSeq.v1

SAMPID	SMATSSCR	SMCENTER	SMRIN	SMTSD	SMUBRID	SMTSISCH	SMTSPAX	SMNABTCH	SMGEBTCH	SMGEBTCHT
GTEX-ZVT2-2526-SM-51MT1	0	B1	7.8	Skin - Sun Exposed	1511	149	1058	BP-43375	LCSET-4207	TrueSeq.v1
GTEX-ZVT3-0126-SM-5GU6I	0	C1	7	Skin - Sun Exposed	1511	572	496	BP-43823	LCSET-4803	TrueSeq.v1
GTEX-ZVT4-0126-SM-51MS1	0	C1	7.5	Skin - Sun Exposed	1511	602	711	BP-43375	LCSET-4207	TrueSeq.v1
GTEX-ZVTK-0426-SM-51MRS	1	C1	7.2	Skin - Sun Exposed	1511	945	1235	BP-43375	LCSET-4207	TrueSeq.v1
GTEX-ZVZO-0126-SM-5A5L9	0	B1	6.9	Skin - Sun Exposed	1511	143	1170	BP-43495	LCSET-4423	TrueSeq.v1
GTEX-ZVZP-2426-SM-59HKJ	0	B1	7.2	Skin - Sun Exposed	1511	158	973	BP-43441	LCSET-4419	TrueSeq.v1
GTEX-ZVZQ-0326-SM-59HLL	0	C1	7.7	Skin - Sun Exposed	1511	1110	1423	BP-43441	LCSET-4419	TrueSeq.v1
GTEX-ZWKS-0126-SM-5SIAA	0	C1	7.1	Skin - Sun Exposed	1511	954	1039	BP-43956	LCSET-5174	TrueSeq.v1
GTEX-ZXES-1826-SM-5E43S	0	B1	6.1	Skin - Sun Exposed	1511	150	910	BP-43495	LCSET-4635	TrueSeq.v1
GTEX-ZXG5-0126-SM-5GIEU	1	B1	6.9	Skin - Sun Exposed	1511	1058	765	BP-43823	LCSET-4796	TrueSeq.v1
GTEX-ZY6K-1826-SM-5GZXK	0	B1	7	Skin - Sun Exposed	1511	268	1259	BP-43507	LCSET-4804	TrueSeq.v1
GTEX-ZYFC-0226-SM-5NQ75	1	C1	7.6	Skin - Sun Exposed	1511	1321	1306	BP-43639	LCSET-4906	TrueSeq.v1
GTEX-ZYFD-0126-SM-5GIDL	0	C1	8.2	Skin - Sun Exposed	1511	525	519	BP-44261	LCSET-5161	TrueSeq.v1
GTEX-ZYFG-2326-SM-5E44B	0	B1	5.9	Skin - Sun Exposed	1511	147	443	BP-44437	LCSET-4635	TrueSeq.v1
GTEX-ZYT6-0226-SM-5NQ6T	0	C1	8.3	Skin - Sun Exposed	1511	1402	587	BP-43529	LCSET-4906	TrueSeq.v1
GTEX-ZYW4-0126-SM-5E44A	1	C1	7.4	Skin - Sun Exposed	1511	745	1069	BP-44437	LCSET-4635	TrueSeq.v1
GTEX-ZYY3-0126-SM-5GZY5	0	C1	6.6	Skin - Sun Exposed	1511	484	696	BP-43693	LCSET-4804	TrueSeq.v1
GTEX-ZZ64-1726-SM-5GZYB	0	B1	8	Skin - Sun Exposed	1511	236	1188	BP-43753	LCSET-4804	TrueSeq.v1
GTEX-ZZPT-0226-SM-5E43X	0	C1	7.2	Skin - Sun Exposed	1511	1094	475	BP-43529	LCSET-4635	TrueSeq.v1

Supplementary Table S3. Primer pairs used for RT-PCR experiments.

**Gene Name**	**Transcript ID**	**Forward Primer (5’-3’)**	**Reverse Primer (5’-3’)**
TNFAIP3	ENST00000612899	CATGGGTGTGTCTGTGGAAG	GCTGGCAACTGGAGTCTCTC
VEGFA	ENST00000523950	GCAGTAGCTGCGCTGATAGA	CCTTGCTGCTCTACCTCCAC
SLC27A4	ENST00000372870	CTTCACCTTCAGGAGGACCA	TGGGATTCTCCCTGTTGTTC
RP11-356I2.4	ENST00000606998.1	CACGTTCATACCTGCCACTG	ACAAGGCTGCCAGAAGATGT
LNC_000057	XLOC_010023	ATAAAGTTGAAGATAAATAGGCACCA	CTCTCTCTCTCTCTCTCACACACACA
LNC_000104	XLOC_023709	GAAAGGGAAAGAGATAGACTACTGGA	ATGAAAGACTGAGAAAAAACAAAACC
LNC_000310	XLOC_070305	CCAACTTTCTTCTGTTCACTTTCTTA	TAGTCCTTTTCTTGTTTGTTTTTCAT
LNC_000311	XLOC_070305	CCAACTTTCTTCTGTTCACTTTCTT	AGTCCTTTTCTTGTTTGTTTTTCAT

Supplementary Table S4. Detailed data obtained and mapping conditions for each sample in RNA-Seq.

**Sample name**	**C_1**	**C_2**	**C_3**	**C_4**	**N_1**	**N_2**	**N_3**	**N_4**
Raw reads	96353430	103811982	100482406	111491218	106459578	84573866	94447152	112828900
Clean reads	91789150	99540764	96144170	107047198	102061624	81167320	90605064	108129618
Total mapped*	85173573 (92.79%)	92315000 (92.74%)	89370499 (92.95%)	99494797 (92.94%)	93562741 (91.67%)	73840947 (90.97%)	81609212 (90.07%)	100243701 (92.71%)
Multiple mapped*	4158857 (4.53%)	3626588 (3.64%)	3198479 (3.33%)	3330127 (3.11%)	6844185 (6.71%)	3804493 (4.69%)	3771308 (4.16%)	3614248 (3.34%)
Uniquely mapped*	81014716 (88.26%)	88688412 (89.1%)	86172020 (89.63%)	96164670 (89.83%)	86718556 (84.97%)	70036454 (86.29%)	77837904 (85.91%)	96629453 (89.36%)
Reads map to '+'strand*	40546609 (44.17%)	44332199 (44.54%)	43112516 (44.84%)	48092028 (44.93%)	43404302 (42.53%)	35032941 (43.16%)	38911651 (42.95%)	48327813 (44.69%)
Reads map to '-'strand*	40468107 (44.09%)	44356213 (44.56%)	43059504 (44.79%)	48072642 (44.91%)	43314254 (42.44%)	35003513 (43.13%)	38926253 (42.96%)	48301640 (44.67%)
Non-splice reads*	65047794 (70.87%)	70812442 (71.14%)	69834764 (72.64%)	77679404 (72.57%)	72229256 (70.77%)	56013723 (69.01%)	65104779 (71.86%)	81949199 (75.79%)
Splice reads*	15966922 (17.4%)	17875970 (17.96%)	16337256 (16.99%)	18485266 (17.27%)	14489300 (14.2%)	14022731 (17.28%)	12733125 (14.05%)	14680254 (13.58%)
Reads mapped in proper pairs*	75531322 (82.29%)	81603896 (81.98%)	79630656 (82.82%)	87997446 (82.2%)	79920816 (78.31%)	64758876 (79.78%)	71727698 (79.17%)	88821342 (82.14%)

*Numbers in the brackets stand for percentages of clean reads.

Supplementary Table S5. Characteristics of the novel lncRNAs identified in this study.

Novel_lncRNA ID	Novel_lncRNA Gene_ID	Gene_Type	Status	Chromosome	Start	End	Strand	Exon number	Length	ORF
LNC_000302	XLOC_069409	lincRNA	Novel_lncRNA	chr8	1753956	1755622	-	2	1255	84
LNC_000311	XLOC_070305	lincRNA	Novel_lncRNA	chr8	85973919	85978858	-	3	2281	129
LNC_000228	XLOC_054681	lincRNA	Novel_lncRNA	chr5	2695138	2696876	+	2	1171	184
LNC_000229	XLOC_054707	lincRNA	Novel_lncRNA	chr5	6774631	6777820	+	2	3015	166
LNC_000217	XLOC_052430	lincRNA	Novel_lncRNA	chr4	1.75E+08	1.75E+08	+	2	1844	140
LNC_000103	XLOC_022757	lincRNA	Novel_lncRNA	chr14	1.05E+08	1.05E+08	-	4	3500	231
LNC_000215	XLOC_052164	lincRNA	Novel_lncRNA	chr4	1.54E+08	1.54E+08	+	3	1168	147
LNC_000214	XLOC_052164	lincRNA	Novel_lncRNA	chr4	1.54E+08	1.54E+08	+	3	3162	147
LNC_000059	XLOC_010833	lincRNA	Novel_lncRNA	chr11	13051501	13054740	+	2	1747	110
LNC_000058	XLOC_010604	lincRNA	Novel_lncRNA	chr11	1043199	1049601	+	2	1912	206
LNC_000211	XLOC_051728	lincRNA	Novel_lncRNA	chr4	1.23E+08	1.23E+08	+	2	395	103
LNC_000102	XLOC_022585	lincRNA	Novel_lncRNA	chr14	98366094	98370286	-	2	3622	155
LNC_000055	XLOC_009995	antisense_lncRNA	Novel_lncRNA	chr10	86960405	86961871	-	2	1385	143
LNC_000054	XLOC_009988	lincRNA	Novel_lncRNA	chr10	86536969	86556167	-	4	1032	162
LNC_000057	XLOC_010023	lincRNA	Novel_lncRNA	chr10	88068564	88089263	-	6	8326	120
LNC_000056	XLOC_010023	lincRNA	Novel_lncRNA	chr10	88068564	88089259	-	5	8305	120
LNC_000051	XLOC_009328	lincRNA	Novel_lncRNA	chr10	29913907	29928614	-	2	1489	98
LNC_000050	XLOC_009103	lincRNA	Novel_lncRNA	chr10	8571497	8594433	-	2	1990	99
LNC_000053	XLOC_009933	lincRNA	Novel_lncRNA	chr10	79685266	79688046	-	2	1883	131
LNC_000052	XLOC_009490	lincRNA	Novel_lncRNA	chr10	45218655	45224445	-	2	289	83
LNC_000114	XLOC_027001	lincRNA	Novel_lncRNA	chr16	17569821	17572438	-	2	1848	122
LNC_000115	XLOC_027065	lincRNA	Novel_lncRNA	chr16	22809921	22812326	-	2	761	90

Novel_lncRNA ID	Novel_lncRNA Gene_ID	Gene_Type	Status	Chromosome	Start	End	Strand	Exon number	Length	ORF
LNC_000116	XLOC_027510	lincRNA	Novel_lncRNA	chr16	63408477	63409663	-	2	857	79
LNC_000117	XLOC_027609	lincRNA	Novel_lncRNA	chr16	68589667	68596281	-	3	1716	200
LNC_000110	XLOC_025142	lincRNA	Novel_lncRNA	chr15	81915407	81926440	-	3	688	94
LNC_000111	XLOC_025251	antisense_lncRNA	Novel_lncRNA	chr15	89107619	89122584	-	2	1356	141
LNC_000112	XLOC_026566	lincRNA	Novel_lncRNA	chr16	85302532	85308600	+	2	1571	130
LNC_000113	XLOC_026670	lincRNA	Novel_lncRNA	chr16	89973785	89974650	+	2	472	66
LNC_000297	XLOC_067763	lincRNA	Novel_lncRNA	chr8	1041962	1045729	+	2	2372	145
LNC_000296	XLOC_067556	lincRNA	Novel_lncRNA	chr7	1.49E+08	1.49E+08	-	2	2120	70
LNC_000295	XLOC_067390	lincRNA	Novel_lncRNA	chr7	1.31E+08	1.31E+08	-	2	658	56
LNC_000294	XLOC_066942	lincRNA	Novel_lncRNA	chr7	94276500	94385514	-	4	2691	142
LNC_000118	XLOC_027710	lincRNA	Novel_lncRNA	chr16	72473685	72478757	-	2	2868	94
LNC_000119	XLOC_027922	lincRNA	Novel_lncRNA	chr16	88184573	88186800	-	2	2146	370
LNC_000291	XLOC_066346	antisense_lncRNA	Novel_lncRNA	chr7	48042778	48052816	-	2	2489	132
LNC_000290	XLOC_066346	antisense_lncRNA	Novel_lncRNA	chr7	48042778	48052791	-	3	1615	116
LNC_000319	XLOC_070960	lincRNA	Novel_lncRNA	chr8	1.45E+08	1.45E+08	-	2	3406	152
LNC_000198	XLOC_048620	antisense_lncRNA	Novel_lncRNA	chr3	15014667	15017887	-	2	1506	87
LNC_000199	XLOC_049351	lincRNA	Novel_lncRNA	chr3	1.04E+08	1.04E+08	-	2	1892	83
LNC_000194	XLOC_048215	lincRNA	Novel_lncRNA	chr3	1.94E+08	1.94E+08	+	2	619	90
LNC_000195	XLOC_048383	lincRNA	Novel_lncRNA	chr3	3914378	3963045	-	2	14091	111
LNC_000196	XLOC_048484	lincRNA	Novel_lncRNA	chr3	7915327	7919836	-	2	3027	137
LNC_000197	XLOC_048594	lincRNA	Novel_lncRNA	chr3	13259055	13263073	-	2	3930	158
LNC_000190	XLOC_047474	antisense_lncRNA	Novel_lncRNA	chr3	1.39E+08	1.39E+08	+	2	2813	131
LNC_000191	XLOC_047586	lincRNA	Novel_lncRNA	chr3	1.44E+08	1.44E+08	+	3	6733	125
LNC_000192	XLOC_047679	lincRNA	Novel_lncRNA	chr3	1.53E+08	1.53E+08	+	2	2858	92

Novel_lncRNA ID	Novel_lncRNA Gene_ID	Gene_Type	Status	Chromosome	Start	End	Strand	Exon number	Length	ORF
LNC_000193	XLOC_047711	antisense_lncRNA	Novel_lncRNA	chr3	1.56E+08	1.56E+08	+	3	2533	116
LNC_000298	XLOC_067846	lincRNA	Novel_lncRNA	chr8	2623434	2666960	+	3	1269	104
LNC_000208	XLOC_051008	lincRNA	Novel_lncRNA	chr4	38155518	38158405	+	2	1465	95
LNC_000337	XLOC_076360	lincRNA	Novel_lncRNA	chrX	275345	276227	-	2	643	214
LNC_000340	XLOC_078144	lincRNA	Novel_lncRNA	chrY	19744977	19756246	+	3	1969	114
LNC_000260	XLOC_059904	lincRNA	Novel_lncRNA	chr6	66259125	66276962	+	3	2743	115
LNC_000060	XLOC_011599	lincRNA	Novel_lncRNA	chr11	65666152	65671704	+	2	348	67
LNC_000061	XLOC_012005	lincRNA	Novel_lncRNA	chr11	88372999	88401040	+	3	1776	112
LNC_000062	XLOC_012392	lincRNA	Novel_lncRNA	chr11	1.2E+08	1.2E+08	+	2	2644	164
LNC_000063	XLOC_012577	lincRNA	Novel_lncRNA	chr11	1.31E+08	1.31E+08	+	2	2053	127
LNC_000064	XLOC_012888	antisense_lncRNA	Novel_lncRNA	chr11	8092360	8101630	-	2	8693	170
LNC_000065	XLOC_012942	lincRNA	Novel_lncRNA	chr11	10927204	10931671	-	2	1889	243
LNC_000066	XLOC_013171	lincRNA	Novel_lncRNA	chr11	35061230	35067730	-	2	5134	171
LNC_000067	XLOC_013785	lincRNA	Novel_lncRNA	chr11	73242767	73243832	-	2	736	97
LNC_000068	XLOC_014248	lincRNA	Novel_lncRNA	chr11	1.1E+08	1.1E+08	-	2	1862	130
LNC_000069	XLOC_014306	lincRNA	Novel_lncRNA	chr11	1.16E+08	1.16E+08	-	3	1661	203
LNC_000101	XLOC_022584	lincRNA	Novel_lncRNA	chr14	98319977	98325828	-	2	914	84
LNC_000100	XLOC_022536	lincRNA	Novel_lncRNA	chr14	95383051	95387284	-	2	526	89
LNC_000107	XLOC_024688	lincRNA	Novel_lncRNA	chr15	47998842	48007907	-	2	5314	131
LNC_000106	XLOC_024598	lincRNA	Novel_lncRNA	chr15	40869269	40872020	-	2	1249	152
LNC_000105	XLOC_024531	lincRNA	Novel_lncRNA	chr15	38730859	38751115	-	3	1343	86
LNC_000104	XLOC_023709	lincRNA	Novel_lncRNA	chr15	70254976	70295670	+	3	4380	154
LNC_000262	XLOC_060045	lincRNA	Novel_lncRNA	chr6	84430227	84526675	+	3	7052	167
LNC_000263	XLOC_060363	lincRNA	Novel_lncRNA	chr6	1.14E+08	1.14E+08	+	2	659	57

Novel_lncRNA ID	Novel_lncRNA Gene_ID	Gene_Type	Status	Chromosome	Start	End	Strand	Exon number	Length	ORF
LNC_000209	XLOC_051025	lincRNA	Novel_lncRNA	chr4	38750233	38753481	+	2	2557	150
LNC_000261	XLOC_060045	lincRNA	Novel_lncRNA	chr6	84352896	84376675	+	2	6258	162
LNC_000266	XLOC_061260	lincRNA	Novel_lncRNA	chr6	1.69E+08	1.69E+08	+	3	1417	192
LNC_000267	XLOC_061394	lincRNA	Novel_lncRNA	chr6	3853336	3868716	-	2	927	99
LNC_000264	XLOC_060669	lincRNA	Novel_lncRNA	chr6	1.22E+08	1.22E+08	+	3	974	84
LNC_000216	XLOC_052343	lincRNA	Novel_lncRNA	chr4	1.66E+08	1.66E+08	+	2	1581	161
LNC_000312	XLOC_070305	lincRNA	Novel_lncRNA	chr8	85973919	85978858	-	3	2811	129
LNC_000313	XLOC_070305	lincRNA	Novel_lncRNA	chr8	85973919	85979068	-	4	2619	129
LNC_000268	XLOC_061467	lincRNA	Novel_lncRNA	chr6	6873338	6875963	-	2	1542	168
LNC_000265	XLOC_060733	lincRNA	Novel_lncRNA	chr6	1.25E+08	1.25E+08	+	2	2668	148
LNC_000316	XLOC_070543	lincRNA	Novel_lncRNA	chr8	1.03E+08	1.03E+08	-	2	1390	224
LNC_000259	XLOC_059702	lincRNA	Novel_lncRNA	chr6	39941935	39963571	+	3	432	77
LNC_000314	XLOC_070305	lincRNA	Novel_lncRNA	chr8	85973919	85979680	-	3	2499	129
LNC_000315	XLOC_070477	antisense_lncRNA	Novel_lncRNA	chr8	99770190	99782715	-	2	971	93
LNC_000183	XLOC_045217	antisense_lncRNA	Novel_lncRNA	chr22	46250982	46256530	+	3	1193	96
LNC_000182	XLOC_045171	lincRNA	Novel_lncRNA	chr22	43917617	43923489	+	4	735	180
LNC_000181	XLOC_044588	lincRNA	Novel_lncRNA	chr22	19688129	19692046	+	3	1043	133
LNC_000180	XLOC_044580	lincRNA	Novel_lncRNA	chr22	19291918	19330183	+	3	798	100
LNC_000187	XLOC_046603	lincRNA	Novel_lncRNA	chr3	47185152	47194864	+	2	2000	125
LNC_000186	XLOC_046173	lincRNA	Novel_lncRNA	chr3	12470820	12477582	+	5	2119	155
LNC_000185	XLOC_045926	lincRNA	Novel_lncRNA	chr22	46139836	46144104	-	2	1989	180
LNC_000184	XLOC_045667	antisense_lncRNA	Novel_lncRNA	chr22	32352399	32355033	-	2	803	126
LNC_000213	XLOC_051838	lincRNA	Novel_lncRNA	chr4	1.28E+08	1.28E+08	+	2	5535	142
LNC_000189	XLOC_046816	lincRNA	Novel_lncRNA	chr3	71897151	71899277	+	2	1370	113

Novel_lncRNA ID	Novel_lncRNA Gene_ID	Gene_Type	Status	Chromosome	Start	End	Strand	Exon number	Length	ORF
LNC_000188	XLOC_046740	antisense_lncRNA	Novel_lncRNA	chr3	59738510	59756691	+	2	2325	103
LNC_000288	XLOC_066001	lincRNA	Novel_lncRNA	chr7	19025604	19053511	-	2	2226	109
LNC_000310	XLOC_070305	lincRNA	Novel_lncRNA	chr8	85973919	85978858	-	2	4538	129
LNC_000212	XLOC_051787	lincRNA	Novel_lncRNA	chr4	1.24E+08	1.24E+08	+	2	5580	122
LNC_000324	XLOC_072882	lincRNA	Novel_lncRNA	chr9	5576655	5609006	-	2	923	74
LNC_000335	XLOC_075031	lincRNA	Novel_lncRNA	chrX	39337705	39387521	+	3	1721	106
LNC_000269	XLOC_061511	lincRNA	Novel_lncRNA	chr6	10349216	10352060	-	2	526	100
LNC_000331	XLOC_073790	lincRNA	Novel_lncRNA	chr9	87197313	87277293	-	5	1712	132
LNC_000210	XLOC_051209	lincRNA	Novel_lncRNA	chr4	57358881	57372827	+	2	6097	150
LNC_000317	XLOC_070768	lincRNA	Novel_lncRNA	chr8	1.27E+08	1.27E+08	-	3	785	72
LNC_000077	XLOC_015346	lincRNA	Novel_lncRNA	chr12	41168353	41172291	+	2	1927	100
LNC_000076	XLOC_014962	lincRNA	Novel_lncRNA	chr12	10723424	10726744	+	2	1945	101
LNC_000075	XLOC_014943	lincRNA	Novel_lncRNA	chr12	9649568	9650496	+	2	827	81
LNC_000074	XLOC_014918	lincRNA	Novel_lncRNA	chr12	9322031	9324356	+	2	1378	124
LNC_000073	XLOC_014918	lincRNA	Novel_lncRNA	chr12	9321995	9340677	+	3	1265	124
LNC_000072	XLOC_014918	lincRNA	Novel_lncRNA	chr12	9321995	9328034	+	3	1410	124
LNC_000071	XLOC_014745	lincRNA	Novel_lncRNA	chr12	5160312	5273966	+	6	853	99
LNC_000070	XLOC_014558	lincRNA	Novel_lncRNA	chr11	1.29E+08	1.29E+08	-	3	2267	153
LNC_000176	XLOC_043128	lincRNA	Novel_lncRNA	chr20	51336304	51338483	-	2	2090	120
LNC_000177	XLOC_043650	antisense_lncRNA	Novel_lncRNA	chr21	37687228	37717235	+	4	598	101
LNC_000174	XLOC_043098	lincRNA	Novel_lncRNA	chr20	47840904	47844401	-	4	1146	162
LNC_000175	XLOC_043098	lincRNA	Novel_lncRNA	chr20	47841680	47844721	-	3	1192	144
LNC_000172	XLOC_042939	lincRNA	Novel_lncRNA	chr20	40299720	40306708	-	2	3383	96
LNC_000173	XLOC_043098	lincRNA	Novel_lncRNA	chr20	47840904	47843495	-	3	967	201

Novel_lncRNA ID	Novel_lncRNA Gene_ID	Gene_Type	Status	Chromosome	Start	End	Strand	Exon number	Length	ORF
LNC_000079	XLOC_016271	lincRNA	Novel_lncRNA	chr12	1.11E+08	1.11E+08	+	2	1508	98
LNC_000078	XLOC_015401	lincRNA	Novel_lncRNA	chr12	46090058	46095367	+	2	3043	104
LNC_000271	XLOC_062017	antisense_lncRNA	Novel_lncRNA	chr6	36240436	36268388	-	8	4100	141
LNC_000270	XLOC_061815	lincRNA	Novel_lncRNA	chr6	29489928	29494143	-	2	1742	132
LNC_000273	XLOC_062461	lincRNA	Novel_lncRNA	chr6	75024612	75076296	-	2	2249	93
LNC_000272	XLOC_062079	lincRNA	Novel_lncRNA	chr6	40886426	40889784	-	2	1528	89
LNC_000275	XLOC_062527	lincRNA	Novel_lncRNA	chr6	77270389	77369159	-	3	1660	140
LNC_000274	XLOC_062527	lincRNA	Novel_lncRNA	chr6	77143552	77316705	-	5	8390	105
LNC_000277	XLOC_063439	lincRNA	Novel_lncRNA	chr6	1.56E+08	1.56E+08	-	2	3952	119
LNC_000276	XLOC_062825	lincRNA	Novel_lncRNA	chr6	1E+08	1E+08	-	2	1726	111
LNC_000279	XLOC_063841	lincRNA	Novel_lncRNA	chr7	7819380	7820193	+	2	751	85
LNC_000278	XLOC_063610	lincRNA	Novel_lncRNA	chr6	1.68E+08	1.68E+08	-	2	2446	121
LNC_000303	XLOC_069485	lincRNA	Novel_lncRNA	chr8	6858787	6865615	-	2	622	75
LNC_000242	XLOC_056704	lincRNA	Novel_lncRNA	chr5	859255	860880	-	2	1530	158
LNC_000305	XLOC_069585	antisense_lncRNA	Novel_lncRNA	chr8	10674576	10681882	-	3	4675	251
LNC_000304	XLOC_069485	lincRNA	Novel_lncRNA	chr8	6860508	6865568	-	2	2035	158
LNC_000307	XLOC_069585	antisense_lncRNA	Novel_lncRNA	chr8	10674576	10682758	-	3	5483	251
LNC_000306	XLOC_069585	antisense_lncRNA	Novel_lncRNA	chr8	10674576	10681886	-	2	7050	251
LNC_000219	XLOC_052817	lincRNA	Novel_lncRNA	chr4	9598987	9605321	-	2	2673	93
LNC_000218	XLOC_052525	lincRNA	Novel_lncRNA	chr4	1.84E+08	1.84E+08	+	2	1281	82
LNC_000318	XLOC_070932	lincRNA	Novel_lncRNA	chr8	1.45E+08	1.45E+08	-	2	1466	115
LNC_000332	XLOC_073790	lincRNA	Novel_lncRNA	chr9	87197313	87277322	-	6	1873	132
LNC_000323	XLOC_072856	lincRNA	Novel_lncRNA	chr9	4879462	4887713	-	2	1430	132
LNC_000289	XLOC_066309	lincRNA	Novel_lncRNA	chr7	44375008	44378887	-	2	3061	176

Novel_lncRNA ID	Novel_lncRNA Gene_ID	Gene_Type	Status	Chromosome	Start	End	Strand	Exon number	Length	ORF
LNC_000322	XLOC_072529	lincRNA	Novel_lncRNA	chr9	1.22E+08	1.22E+08	+	2	1016	115
LNC_000339	XLOC_077966	lincRNA	Novel_lncRNA	chrY	7091959	7098262	+	2	6061	151
LNC_000321	XLOC_071884	antisense_lncRNA	Novel_lncRNA	chr9	70601713	70604529	+	2	1546	119
LNC_000320	XLOC_071282	lincRNA	Novel_lncRNA	chr9	29566868	29575210	+	2	1346	84
LNC_000169	XLOC_042469	lincRNA	Novel_lncRNA	chr20	1860128	1875849	-	3	7694	177
LNC_000168	XLOC_042436	lincRNA	Novel_lncRNA	chr20	1413752	1418936	-	3	759	64
LNC_000165	XLOC_042274	lincRNA	Novel_lncRNA	chr20	56975139	56978703	+	3	1217	197
LNC_000164	XLOC_042202	lincRNA	Novel_lncRNA	chr20	51349769	51367200	+	2	1043	112
LNC_000167	XLOC_042335	lincRNA	Novel_lncRNA	chr20	60060955	60067655	+	2	5562	154
LNC_000166	XLOC_042300	lincRNA	Novel_lncRNA	chr20	58622893	58626352	+	2	1518	195
LNC_000161	XLOC_041654	lincRNA	Novel_lncRNA	chr20	11375792	11433037	+	2	1314	100
LNC_000160	XLOC_040607	lincRNA	Novel_lncRNA	chr2	1.71E+08	1.71E+08	-	2	2370	93
LNC_000163	XLOC_042040	lincRNA	Novel_lncRNA	chr20	40656738	40679878	+	2	735	135
LNC_000162	XLOC_041665	lincRNA	Novel_lncRNA	chr20	11943147	11944726	+	2	1094	97
LNC_000002	XLOC_000119	lincRNA	Novel_lncRNA	GL000219.1	48990	51090	-	2	1995	150
LNC_000003	XLOC_000348	lincRNA	Novel_lncRNA	KI270742.1	20129	34973	-	2	4305	145
LNC_000246	XLOC_057913	lincRNA	Novel_lncRNA	chr5	1.09E+08	1.09E+08	-	2	1402	92
LNC_000001	XLOC_000086	lincRNA	Novel_lncRNA	GL000205.2	137757	140747	-	2	721	111
LNC_000006	XLOC_001537	lincRNA	Novel_lncRNA	chr1	58718104	58751903	+	2	1718	95
LNC_000007	XLOC_001759	lincRNA	Novel_lncRNA	chr1	78667025	78672485	+	2	5354	117
LNC_000004	XLOC_000376	lincRNA	Novel_lncRNA	KI270744.1	41741	44618	+	2	1334	118
LNC_000005	XLOC_000466	lincRNA	Novel_lncRNA	chr1	2215380	2216345	+	2	780	187
LNC_000334	XLOC_075028	lincRNA	Novel_lncRNA	chrX	39017187	39020435	+	2	1740	91
LNC_000247	XLOC_058349	lincRNA	Novel_lncRNA	chr5	1.45E+08	1.45E+08	-	3	4215	169

Novel_lncRNA ID	Novel_lncRNA Gene_ID	Gene_Type	Status	Chromosome	Start	End	Strand	Exon number	Length	ORF
LNC_000008	XLOC_001903	lincRNA	Novel_lncRNA	chr1	94563192	94636988	+	2	1620	135
LNC_000009	XLOC_001910	lincRNA	Novel_lncRNA	chr1	95092493	95094817	+	2	1327	101
LNC_000248	XLOC_058349	lincRNA	Novel_lncRNA	chr5	1.45E+08	1.45E+08	-	2	2855	148
LNC_000249	XLOC_058402	lincRNA	Novel_lncRNA	chr5	1.46E+08	1.46E+08	-	2	3437	94
LNC_000237	XLOC_056320	lincRNA	Novel_lncRNA	chr5	1.53E+08	1.53E+08	+	5	3429	110
LNC_000333	XLOC_074661	lincRNA	Novel_lncRNA	chrX	1240670	1250042	+	2	1583	124
LNC_000088	XLOC_018890	lincRNA	Novel_lncRNA	chr13	80757216	80824945	+	4	1510	114
LNC_000089	XLOC_019043	lincRNA	Novel_lncRNA	chr13	98784933	98787957	+	2	1912	160
LNC_000233	XLOC_055734	lincRNA	Novel_lncRNA	chr5	1.16E+08	1.16E+08	+	2	865	130
LNC_000082	XLOC_017266	antisense_lncRNA	Novel_lncRNA	chr12	53117316	53123580	-	2	1337	172
LNC_000083	XLOC_017609	antisense_lncRNA	Novel_lncRNA	chr12	80315839	80319218	-	2	2468	94
LNC_000080	XLOC_016795	lincRNA	Novel_lncRNA	chr12	12968124	12974341	-	2	2316	127
LNC_000081	XLOC_016984	lincRNA	Novel_lncRNA	chr12	31750349	31753330	-	2	1188	97
LNC_000086	XLOC_018334	lincRNA	Novel_lncRNA	chr13	20295682	20326221	+	3	1063	129
LNC_000087	XLOC_018632	lincRNA	Novel_lncRNA	chr13	48581111	48599869	+	3	971	159
LNC_000084	XLOC_017749	lincRNA	Novel_lncRNA	chr12	94701555	94711863	-	2	7789	140
LNC_000085	XLOC_018227	lincRNA	Novel_lncRNA	chr12	1.31E+08	1.31E+08	-	2	1267	157
LNC_000206	XLOC_050889	lincRNA	Novel_lncRNA	chr4	14271127	14274816	+	2	3585	117
LNC_000327	XLOC_073141	lincRNA	Novel_lncRNA	chr9	23684973	23688844	-	2	3381	86
LNC_000293	XLOC_066867	lincRNA	Novel_lncRNA	chr7	91515050	91555096	-	3	711	82
LNC_000207	XLOC_050903	lincRNA	Novel_lncRNA	chr4	14894093	14909459	+	3	442	111
LNC_000236	XLOC_056273	lincRNA	Novel_lncRNA	chr5	1.49E+08	1.49E+08	+	2	999	102
LNC_000292	XLOC_066753	lincRNA	Novel_lncRNA	chr7	81250801	81267568	-	2	1459	104
LNC_000150	XLOC_039013	lincRNA	Novel_lncRNA	chr2	44267378	44270751	-	2	1704	121

Novel_lncRNA ID	Novel_lncRNA Gene_ID	Gene_Type	Status	Chromosome	Start	End	Strand	Exon number	Length	ORF
LNC_000151	XLOC_039197	lincRNA	Novel_lncRNA	chr2	60336696	60339790	-	2	2214	119
LNC_000152	XLOC_039388	lincRNA	Novel_lncRNA	chr2	72009746	72015293	-	2	4687	147
LNC_000153	XLOC_039388	lincRNA	Novel_lncRNA	chr2	72009746	72033469	-	3	5202	194
LNC_000154	XLOC_039388	lincRNA	Novel_lncRNA	chr2	72009746	72034055	-	2	6498	223
LNC_000155	XLOC_039530	lincRNA	Novel_lncRNA	chr2	88136485	88138896	-	2	1379	96
LNC_000156	XLOC_039540	lincRNA	Novel_lncRNA	chr2	88688244	88691466	-	2	777	134
LNC_000157	XLOC_040152	lincRNA	Novel_lncRNA	chr2	1.38E+08	1.38E+08	-	4	965	95
LNC_000158	XLOC_040301	lincRNA	Novel_lncRNA	chr2	1.51E+08	1.51E+08	-	3	6133	106
LNC_000159	XLOC_040444	lincRNA	Novel_lncRNA	chr2	1.57E+08	1.57E+08	-	2	2519	156
LNC_000251	XLOC_058686	lincRNA	Novel_lncRNA	chr5	1.69E+08	1.69E+08	-	2	3649	141
LNC_000250	XLOC_058535	lincRNA	Novel_lncRNA	chr5	1.52E+08	1.52E+08	-	2	496	65
LNC_000257	XLOC_059453	lincRNA	Novel_lncRNA	chr6	29945966	29949287	+	2	2376	157
LNC_000256	XLOC_059203	lincRNA	Novel_lncRNA	chr6	14431743	14463397	+	2	6755	102
LNC_000255	XLOC_059131	lincRNA	Novel_lncRNA	chr6	8788752	8792275	+	2	2014	120
LNC_000254	XLOC_059018	lincRNA	Novel_lncRNA	chr6	4635989	4637950	+	2	1508	122
LNC_000011	XLOC_002195	lincRNA	Novel_lncRNA	chr1	1.17E+08	1.17E+08	+	2	1625	119
LNC_000010	XLOC_002130	lincRNA	Novel_lncRNA	chr1	1.13E+08	1.13E+08	+	2	1300	177
LNC_000013	XLOC_002479	lincRNA	Novel_lncRNA	chr1	1.52E+08	1.52E+08	+	2	2656	90
LNC_000012	XLOC_002397	lincRNA	Novel_lncRNA	chr1	1.49E+08	1.49E+08	+	2	1465	99
LNC_000015	XLOC_002571	lincRNA	Novel_lncRNA	chr1	1.55E+08	1.55E+08	+	3	1334	128
LNC_000014	XLOC_002534	antisense_lncRNA	Novel_lncRNA	chr1	1.54E+08	1.54E+08	+	2	1345	94
LNC_000017	XLOC_002999	lincRNA	Novel_lncRNA	chr1	1.84E+08	1.84E+08	+	4	1178	78
LNC_000016	XLOC_002901	lincRNA	Novel_lncRNA	chr1	1.79E+08	1.79E+08	+	2	936	153
LNC_000019	XLOC_003421	lincRNA	Novel_lncRNA	chr1	2.19E+08	2.19E+08	+	2	931	116

Novel_lncRNA ID	Novel_lncRNA Gene_ID	Gene_Type	Status	Chromosome	Start	End	Strand	Exon number	Length	ORF
LNC_000018	XLOC_003268	lincRNA	Novel_lncRNA	chr1	2.05E+08	2.05E+08	+	2	1763	119
LNC_000329	XLOC_073645	antisense_lncRNA	Novel_lncRNA	chr9	69130999	69171123	-	2	511	77
LNC_000328	XLOC_073337	lincRNA	Novel_lncRNA	chr9	36477502	36482010	-	2	762	70
LNC_000258	XLOC_059651	lincRNA	Novel_lncRNA	chr6	36122479	36124122	+	2	946	136
LNC_000099	XLOC_022153	lincRNA	Novel_lncRNA	chr14	63155186	63158426	-	2	2285	149
LNC_000098	XLOC_021043	lincRNA	Novel_lncRNA	chr14	63197439	63202289	+	2	1653	98
LNC_000341	XLOC_078297	lincRNA	Novel_lncRNA	chrY	7820917	7823136	-	3	1332	101
LNC_000091	XLOC_019789	lincRNA	Novel_lncRNA	chr13	73582065	73593789	-	2	1247	79
LNC_000090	XLOC_019582	lincRNA	Novel_lncRNA	chr13	45306830	45329776	-	2	1039	106
LNC_000093	XLOC_019982	lincRNA	Novel_lncRNA	chr13	85966270	85996026	-	3	996	118
LNC_000092	XLOC_019894	lincRNA	Novel_lncRNA	chr13	77353401	77375044	-	2	1002	109
LNC_000095	XLOC_020130	lincRNA	Novel_lncRNA	chr13	1.06E+08	1.06E+08	-	2	2962	82
LNC_000094	XLOC_019982	lincRNA	Novel_lncRNA	chr13	85966270	85997935	-	2	1229	118
LNC_000097	XLOC_021040	lincRNA	Novel_lncRNA	chr14	63178163	63181409	+	2	2336	160
LNC_000096	XLOC_021031	lincRNA	Novel_lncRNA	chr14	61113797	61121082	+	2	6986	178
LNC_000244	XLOC_056804	lincRNA	Novel_lncRNA	chr5	6515735	6517187	-	2	584	71
LNC_000325	XLOC_072984	lincRNA	Novel_lncRNA	chr9	13881674	14018300	-	7	1074	94
LNC_000245	XLOC_057280	lincRNA	Novel_lncRNA	chr5	60503438	60505554	-	2	1141	98
LNC_000220	XLOC_052859	lincRNA	Novel_lncRNA	chr4	12887752	12892857	-	2	2706	100
LNC_000147	XLOC_037982	lincRNA	Novel_lncRNA	chr2	2.17E+08	2.17E+08	+	4	1250	96
LNC_000146	XLOC_036816	lincRNA	Novel_lncRNA	chr2	1.22E+08	1.22E+08	+	2	1485	122
LNC_000145	XLOC_036743	lincRNA	Novel_lncRNA	chr2	1.22E+08	1.22E+08	+	2	1411	172
LNC_000144	XLOC_036634	lincRNA	Novel_lncRNA	chr2	1.18E+08	1.18E+08	+	2	4302	128
LNC_000143	XLOC_036634	lincRNA	Novel_lncRNA	chr2	1.18E+08	1.18E+08	+	2	4288	128

Novel_lncRNA ID	Novel_lncRNA Gene_ID	Gene_Type	Status	Chromosome	Start	End	Strand	Exon number	Length	ORF
LNC_000142	XLOC_035982	lincRNA	Novel_lncRNA	chr2	71696870	71743896	+	2	1218	121
LNC_000141	XLOC_035660	lincRNA	Novel_lncRNA	chr2	40717091	40721349	+	2	2732	105
LNC_000140	XLOC_035648	lincRNA	Novel_lncRNA	chr2	39465593	39466902	+	2	1172	195
LNC_000226	XLOC_053612	lincRNA	Novel_lncRNA	chr4	84526892	84542704	-	5	2237	80
LNC_000227	XLOC_054500	lincRNA	Novel_lncRNA	chr4	1.85E+08	1.85E+08	-	2	2617	160
LNC_000224	XLOC_053509	lincRNA	Novel_lncRNA	chr4	75178672	75181073	-	3	544	131
LNC_000225	XLOC_053612	lincRNA	Novel_lncRNA	chr4	84526892	84538511	-	3	1890	70
LNC_000222	XLOC_053170	antisense_lncRNA	Novel_lncRNA	chr4	37602311	37603777	-	2	1383	122
LNC_000223	XLOC_053220	lincRNA	Novel_lncRNA	chr4	41910873	41934652	-	3	803	93
LNC_000149	XLOC_038926	lincRNA	Novel_lncRNA	chr2	38186592	38193192	-	2	1087	91
LNC_000148	XLOC_038906	antisense_lncRNA	Novel_lncRNA	chr2	36518290	36528589	-	2	1516	80
LNC_000024	XLOC_004009	lincRNA	Novel_lncRNA	chr1	5766803	5771462	-	2	1627	106
LNC_000025	XLOC_004012	lincRNA	Novel_lncRNA	chr1	5855250	5861992	-	2	6238	251
LNC_000026	XLOC_005313	lincRNA	Novel_lncRNA	chr1	91544894	91569495	-	4	2811	183
LNC_000027	XLOC_005944	antisense_lncRNA	Novel_lncRNA	chr1	1.53E+08	1.53E+08	-	3	4315	125
LNC_000020	XLOC_003421	lincRNA	Novel_lncRNA	chr1	2.19E+08	2.19E+08	+	2	825	108
LNC_000021	XLOC_003434	lincRNA	Novel_lncRNA	chr1	2.19E+08	2.19E+08	+	2	2464	110
LNC_000022	XLOC_003928	lincRNA	Novel_lncRNA	chr1	1381875	1383115	-	2	1049	109
LNC_000023	XLOC_004009	lincRNA	Novel_lncRNA	chr1	5766803	5771454	-	2	1624	106
LNC_000243	XLOC_056803	lincRNA	Novel_lncRNA	chr5	6510524	6512801	-	2	1243	104
LNC_000028	XLOC_005944	antisense_lncRNA	Novel_lncRNA	chr1	1.53E+08	1.53E+08	-	4	3148	125
LNC_000029	XLOC_005944	antisense_lncRNA	Novel_lncRNA	chr1	1.53E+08	1.53E+08	-	5	3217	125
LNC_000280	XLOC_063904	lincRNA	Novel_lncRNA	chr7	11250465	11252721	+	2	1478	80
LNC_000338	XLOC_076537	antisense_lncRNA	Novel_lncRNA	chrX	14691712	14757825	-	3	955	119

Novel_lncRNA ID	Novel_lncRNA Gene_ID	Gene_Type	Status	Chromosome	Start	End	Strand	Exon number	Length	ORF
LNC_000281	XLOC_063998	lincRNA	Novel_lncRNA	chr7	16990248	17033228	+	2	11910	112
LNC_000336	XLOC_075959	lincRNA	Novel_lncRNA	chrX	1.27E+08	1.27E+08	+	2	394	62
LNC_000240	XLOC_056599	lincRNA	Novel_lncRNA	chr5	1.78E+08	1.78E+08	+	3	989	163
LNC_000326	XLOC_072984	lincRNA	Novel_lncRNA	chr9	13939786	14018824	-	7	1224	83
LNC_000241	XLOC_056617	lincRNA	Novel_lncRNA	chr5	1.79E+08	1.79E+08	+	3	1077	106
LNC_000232	XLOC_055330	lincRNA	Novel_lncRNA	chr5	79547316	79579557	+	3	1671	121
LNC_000178	XLOC_043785	lincRNA	Novel_lncRNA	chr21	44748926	44752598	+	2	2881	245
LNC_000330	XLOC_073790	lincRNA	Novel_lncRNA	chr9	87195383	87198243	-	2	1205	89
LNC_000179	XLOC_043844	lincRNA	Novel_lncRNA	chr21	6359083	6376027	-	2	2077	170
LNC_000253	XLOC_058927	lincRNA	Novel_lncRNA	chr6	744937	747527	+	2	1368	145
LNC_000285	XLOC_064785	lincRNA	Novel_lncRNA	chr7	76786514	76822133	+	2	2214	103
LNC_000299	XLOC_067871	antisense_lncRNA	Novel_lncRNA	chr8	6542681	6551374	+	2	8549	149
LNC_000235	XLOC_056256	lincRNA	Novel_lncRNA	chr5	1.48E+08	1.48E+08	+	2	719	98
LNC_000039	XLOC_007520	lincRNA	Novel_lncRNA	chr10	10798332	10847120	+	2	1544	130
LNC_000038	XLOC_007429	antisense_lncRNA	Novel_lncRNA	chr10	4871347	4929712	+	3	2088	87
LNC_000239	XLOC_056439	lincRNA	Novel_lncRNA	chr5	1.61E+08	1.61E+08	+	3	1773	117
LNC_000238	XLOC_056320	lincRNA	Novel_lncRNA	chr5	1.53E+08	1.53E+08	+	2	3129	110
LNC_000234	XLOC_056024	lincRNA	Novel_lncRNA	chr5	1.29E+08	1.29E+08	+	2	1542	92
LNC_000033	XLOC_006163	antisense_lncRNA	Novel_lncRNA	chr1	1.6E+08	1.6E+08	-	2	1642	163
LNC_000032	XLOC_006151	antisense_lncRNA	Novel_lncRNA	chr1	1.6E+08	1.6E+08	-	2	975	120
LNC_000031	XLOC_006149	lincRNA	Novel_lncRNA	chr1	1.6E+08	1.6E+08	-	2	1579	98
LNC_000030	XLOC_005944	antisense_lncRNA	Novel_lncRNA	chr1	1.53E+08	1.53E+08	-	3	7511	166
LNC_000037	XLOC_007418	lincRNA	Novel_lncRNA	chr10	3961761	3964937	+	2	2153	113
LNC_000036	XLOC_007130	lincRNA	Novel_lncRNA	chr1	2.31E+08	2.31E+08	-	2	326	70

Novel_lncRNA ID	Novel_lncRNA Gene_ID	Gene_Type	Status	Chromosome	Start	End	Strand	Exon number	Length	ORF
LNC_000035	XLOC_007130	lincRNA	Novel_lncRNA	chr1	2.31E+08	2.31E+08	-	2	396	70
LNC_000034	XLOC_006923	lincRNA	Novel_lncRNA	chr1	2.21E+08	2.22E+08	-	2	624	65
LNC_000132	XLOC_032497	antisense_lncRNA	Novel_lncRNA	chr19	8415767	8417033	+	2	491	84
LNC_000133	XLOC_032873	lincRNA	Novel_lncRNA	chr19	23986237	23997129	+	4	1732	144
LNC_000130	XLOC_032242	lincRNA	Novel_lncRNA	chr18	73400378	73691258	-	6	1385	273
LNC_000131	XLOC_032242	lincRNA	Novel_lncRNA	chr18	73647032	73655335	-	3	1587	273
LNC_000136	XLOC_034343	lincRNA	Novel_lncRNA	chr19	27724462	27729938	-	2	4648	108
LNC_000137	XLOC_034673	antisense_lncRNA	Novel_lncRNA	chr19	43414732	43436845	-	3	821	122
LNC_000134	XLOC_033218	antisense_lncRNA	Novel_lncRNA	chr19	42870736	42912933	+	2	3387	196
LNC_000135	XLOC_033399	lincRNA	Novel_lncRNA	chr19	49221866	49236637	+	2	441	79
LNC_000231	XLOC_055235	lincRNA	Novel_lncRNA	chr5	70865159	70868674	+	2	941	127
LNC_000138	XLOC_035235	lincRNA	Novel_lncRNA	chr2	9752906	9757273	+	2	3266	175
LNC_000139	XLOC_035415	lincRNA	Novel_lncRNA	chr2	21266177	21286838	+	2	2212	99
LNC_000252	XLOC_058744	lincRNA	Novel_lncRNA	chr5	1.74E+08	1.74E+08	-	2	1480	125
LNC_000230	XLOC_054777	lincRNA	Novel_lncRNA	chr5	14888694	14889945	+	2	766	117
LNC_000170	XLOC_042620	lincRNA	Novel_lncRNA	chr20	17815797	17829728	-	2	696	101
LNC_000171	XLOC_042733	lincRNA	Novel_lncRNA	chr20	24878892	24887427	-	2	7787	137
LNC_000309	XLOC_069864	lincRNA	Novel_lncRNA	chr8	36959822	36981870	-	4	1352	112
LNC_000308	XLOC_069711	antisense_lncRNA	Novel_lncRNA	chr8	19390895	19403126	-	3	1032	123
LNC_000200	XLOC_050257	lincRNA	Novel_lncRNA	chr3	1.77E+08	1.77E+08	-	2	3722	105
LNC_000201	XLOC_050458	lincRNA	Novel_lncRNA	chr3	1.96E+08	1.96E+08	-	2	5324	279
LNC_000202	XLOC_050625	lincRNA	Novel_lncRNA	chr4	2621141	2623482	+	2	861	95
LNC_000203	XLOC_050697	lincRNA	Novel_lncRNA	chr4	4755576	4764792	+	2	8331	134
LNC_000204	XLOC_050735	lincRNA	Novel_lncRNA	chr4	6763518	6765347	+	2	431	79

Novel_lncRNA ID	Novel_lncRNA Gene_ID	Gene_Type	Status	Chromosome	Start	End	Strand	Exon number	Length	ORF
LNC_000205	XLOC_050838	lincRNA	Novel_lncRNA	chr4	11328629	11337686	+	3	515	92
LNC_000048	XLOC_009036	lincRNA	Novel_lncRNA	chr10	3754601	3758746	-	3	2282	163
LNC_000049	XLOC_009083	lincRNA	Novel_lncRNA	chr10	6255493	6256589	-	2	621	132
LNC_000046	XLOC_008685	antisense_lncRNA	Novel_lncRNA	chr10	1.14E+08	1.14E+08	+	2	342	63
LNC_000047	XLOC_009031	lincRNA	Novel_lncRNA	chr10	3462440	3467750	-	2	3968	161
LNC_000044	XLOC_008644	lincRNA	Novel_lncRNA	chr10	1.11E+08	1.11E+08	+	3	1900	133
LNC_000045	XLOC_008685	antisense_lncRNA	Novel_lncRNA	chr10	1.14E+08	1.14E+08	+	3	505	92
LNC_000042	XLOC_008208	antisense_lncRNA	Novel_lncRNA	chr10	76348631	76354723	+	2	6000	123
LNC_000043	XLOC_008644	lincRNA	Novel_lncRNA	chr10	1.11E+08	1.11E+08	+	4	11786	151
LNC_000040	XLOC_007917	lincRNA	Novel_lncRNA	chr10	47207444	47225226	+	4	941	105
LNC_000041	XLOC_008034	lincRNA	Novel_lncRNA	chr10	63630323	63634497	+	2	1241	248
LNC_000121	XLOC_028742	lincRNA	Novel_lncRNA	chr17	42913899	42923510	+	2	1238	93
LNC_000120	XLOC_027950	lincRNA	Novel_lncRNA	chr16	89604404	89606206	-	2	1676	251
LNC_000123	XLOC_029417	lincRNA	Novel_lncRNA	chr17	83052518	83055072	+	2	2198	170
LNC_000122	XLOC_029206	lincRNA	Novel_lncRNA	chr17	73955433	73959260	+	2	977	174
LNC_000125	XLOC_030446	lincRNA	Novel_lncRNA	chr17	50886283	50893548	-	2	1646	112
LNC_000124	XLOC_029447	lincRNA	Novel_lncRNA	chr17	198759	201400	-	2	2238	125
LNC_000127	XLOC_030980	lincRNA	Novel_lncRNA	chr18	10561662	10566861	+	2	4364	188
LNC_000126	XLOC_030975	lincRNA	Novel_lncRNA	chr18	10405233	10421748	+	2	1964	107
LNC_000129	XLOC_031801	lincRNA	Novel_lncRNA	chr18	30325126	30387616	-	3	1680	99
LNC_000128	XLOC_031386	lincRNA	Novel_lncRNA	chr18	63828353	63833608	+	2	2182	96
LNC_000282	XLOC_064511	lincRNA	Novel_lncRNA	chr7	64572315	64574832	+	3	989	111
LNC_000283	XLOC_064774	lincRNA	Novel_lncRNA	chr7	76680016	76689485	+	4	4042	119
LNC_000284	XLOC_064785	lincRNA	Novel_lncRNA	chr7	76775035	76790060	+	2	12754	126

Novel_lncRNA ID	Novel_lncRNA Gene_ID	Gene_Type	Status	Chromosome	Start	End	Strand	Exon number	Length	ORF
LNC_000109	XLOC_024895	lincRNA	Novel_lncRNA	chr15	64730988	64737203	-	2	1927	161
LNC_000286	XLOC_065217	lincRNA	Novel_lncRNA	chr7	1.21E+08	1.21E+08	+	2	941	121
LNC_000287	XLOC_065714	lincRNA	Novel_lncRNA	chr7	1.53E+08	1.53E+08	+	4	1793	128
LNC_000301	XLOC_068791	lincRNA	Novel_lncRNA	chr8	95195550	95203352	+	2	4707	150
LNC_000221	XLOC_053121	lincRNA	Novel_lncRNA	chr4	26098573	26110517	-	2	510	142
LNC_000108	XLOC_024712	antisense_lncRNA	Novel_lncRNA	chr15	48262721	48273781	-	3	812	93
LNC_000300	XLOC_067961	lincRNA	Novel_lncRNA	chr8	8919815	8937545	+	3	2372	111

Supplementary Table S6. KEGG pathways based on differentially expressed mRNAs between the CAD and healthy control subjects.

#Term	ID	Input number	Background number	P-Value	Corrected P-Value
Alzheimer's disease	hsa05010	68	168	0.000184	0.044807
Non-alcoholic fatty liver disease (NAFLD)	hsa04932	61	151	0.000398	0.044807
Oxidative phosphorylation	hsa00190	55	133	0.000480	0.044807
Huntington's disease	hsa05016	68	183	0.001179	0.082525
Parkinson's disease	hsa05012	55	143	0.001806	0.093954
Metabolic pathways	hsa01100	341	1213	0.002013	0.093954
Carbon metabolism	hsa01200	43	106	0.002477	0.099067
cGMP-PKG signaling pathway	hsa04022	58	167	0.008071	0.265728
Citrate cycle (TCA cycle)	hsa00020	16	30	0.008771	0.265728
Biosynthesis of amino acids	hsa01230	30	74	0.010298	0.265728
Peroxisome	hsa04146	32	81	0.010971	0.265728
Steroid biosynthesis	hsa00100	12	20	0.011549	0.265728
PPAR signaling pathway	hsa03320	28	69	0.012743	0.265728
Fatty acid metabolism	hsa01212	21	47	0.013286	0.265728
Ribosome	hsa03010	46	134	0.019464	0.36332
2-Oxocarboxylic acid metabolism	hsa01210	10	17	0.022355	0.379369
Fatty acid degradation	hsa00071	19	44	0.023033	0.379369
Fatty acid elongation	hsa00062	12	23	0.024425	0.379944
Pathways in cancer	hsa05200	97	327	0.027982	0.412361
HTLV-I infection	hsa05166	79	261	0.031448	0.440274
Glyoxylate and dicarboxylate metabolism	hsa00630	12	25	0.037464	0.482967
Valine, leucine and isoleucine degradation	hsa00280	18	44	0.038122	0.482967
Cardiac muscle contraction	hsa04260	28	78	0.039672	0.482967

#Term	ID	Input number	Background number	P-Value	Corrected P-Value
Prostate cancer	hsa05215	31	89	0.042020	0.490229
Pyruvate metabolism	hsa00620	16	40	0.055585	0.589577
Biosynthesis of unsaturated fatty acids	hsa01040	10	21	0.056852	0.589577
Terpenoid backbone biosynthesis	hsa00900	10	21	0.056852	0.589577
Estrogen signaling pathway	hsa04915	32	100	0.083257	0.832567
Thyroid hormone synthesis	hsa04918	24	72	0.091634	0.884738
Cell cycle	hsa04110	38	124	0.095338	0.889823
Synthesis and degradation of ketone bodies	hsa00072	5	9	0.110801	1
Osteoclast differentiation	hsa04380	39	131	0.118896	1
Protein processing in endoplasmic reticulum	hsa04141	48	168	0.137780	1
Proteasome	hsa03050	15	44	0.141687	1
GnRH signaling pathway	hsa04912	28	92	0.142378	1
Glycolysis / Gluconeogenesis	hsa00010	21	66	0.143710	1
Glycerolipid metabolism	hsa00561	18	55	0.143858	1
Legionellosis	hsa05134	18	55	0.143858	1
One carbon pool by folate	hsa00670	8	20	0.149478	1
Viral carcinogenesis	hsa05203	57	206	0.159374	1
Bladder cancer	hsa05219	13	38	0.160934	1
Glutathione metabolism	hsa00480	16	49	0.163065	1
TNF signaling pathway	hsa04668	32	110	0.171939	1
Propanoate metabolism	hsa00640	10	28	0.174359	1
alpha-Linolenic acid metabolism	hsa00592	9	25	0.186515	1
Arachidonic acid metabolism	hsa00590	19	62	0.192028	1
AMPK signaling pathway	hsa04152	35	124	0.197964	1
Insulin secretion	hsa04911	25	86	0.208120	1

#Term	ID	Input number	Background number	P-Value	Corrected P-Value
Vascular smooth muscle contraction	hsa04270	34	121	0.208273	1
Cell adhesion molecules (CAMs)	hsa04514	40	145	0.212926	1
Adrenergic signaling in cardiomyocytes	hsa04261	41	149	0.213480	1
Oocyte meiosis	hsa04114	31	110	0.217109	1
ECM-receptor interaction	hsa04512	25	87	0.222092	1
Acute myeloid leukemia	hsa05221	17	57	0.235553	1
Steroid hormone biosynthesis	hsa00140	17	57	0.235553	1
Circadian rhythm	hsa04710	10	31	0.245156	1
Glycosaminoglycan biosynthesis - chondroitin sulfate / dermatan sulfate	hsa00532	7	20	0.245359	1
Fatty acid biosynthesis	hsa00061	5	13	0.249550	1
Non-homologous end-joining	hsa03450	5	13	0.249550	1
Dorso-ventral axis formation	hsa04320	8	24	0.256036	1
Adipocytokine signaling pathway	hsa04920	20	70	0.260350	1
Small cell lung cancer	hsa05222	24	86	0.265545	1
Ovarian steroidogenesis	hsa04913	15	51	0.268049	1
HIF-1 signaling pathway	hsa04066	29	106	0.268824	1
Sulfur metabolism	hsa00920	4	10	0.270510	1
Ubiquinone and other terpenoid-quinone biosynthesis	hsa00130	4	10	0.270510	1
Focal adhesion	hsa04510	54	207	0.275446	1
Notch signaling pathway	hsa04330	14	48	0.286441	1
Fanconi anemia pathway	hsa03460	15	53	0.309993	1
VEGF signaling pathway	hsa04370	17	61	0.312151	1
Melanogenesis	hsa04916	27	101	0.313352	1
Circadian entrainment	hsa04713	26	97	0.313640	1
Transcriptional misregulation in cancer	hsa05202	46	179	0.325680	1

#Term	ID	Input number	Background number	P-Value	Corrected P-Value
Oxytocin signaling pathway	hsa04921	41	159	0.330776	1
Colorectal cancer	hsa05210	17	62	0.332344	1
Renal cell carcinoma	hsa05211	18	66	0.332444	1
Axon guidance	hsa04360	33	127	0.338979	1
Mismatch repair	hsa03430	7	23	0.343826	1
Protein export	hsa03060	7	23	0.343826	1
Antigen processing and presentation	hsa04612	21	79	0.350246	1
Tyrosine metabolism	hsa00350	10	35	0.351556	1
Mineral absorption	hsa04978	14	51	0.353689	1
Endometrial cancer	hsa05213	14	52	0.376746	1
ABC transporters	hsa02010	12	44	0.378394	1
Glycine, serine and threonine metabolism	hsa00260	11	40	0.378978	1
Fructose and mannose metabolism	hsa00051	9	32	0.379349	1
Arginine and proline metabolism	hsa00330	16	61	0.396296	1
Caffeine metabolism	hsa00232	2	5	0.397808	1
Cyanoamino acid metabolism	hsa00460	2	5	0.397808	1
Phenylalanine, tyrosine and tryptophan biosynthesis	hsa00400	2	5	0.397808	1
NOD-like receptor signaling pathway	hsa04621	15	57	0.398135	1
N-Glycan biosynthesis	hsa00510	13	49	0.401830	1
Thyroid hormone signaling pathway	hsa04919	30	119	0.403521	1
Epstein-Barr virus infection	hsa05169	50	202	0.403573	1
Wnt signaling pathway	hsa04310	35	140	0.407828	1
Linoleic acid metabolism	hsa00591	8	29	0.410538	1
Thyroid cancer	hsa05216	8	29	0.410538	1
MAPK signaling pathway	hsa04010	63	257	0.412660	1

#Term	ID	Input number	Background number	P-Value	Corrected P-Value
Cocaine addiction	hsa05030	13	50	0.426108	1
Proteoglycans in cancer	hsa05205	50	204	0.427641	1
Hepatitis B	hsa05161	36	146	0.434216	1
Glycerophospholipid metabolism	hsa00564	23	92	0.437703	1
Cholinergic synapse	hsa04725	28	113	0.440336	1
Basal cell carcinoma	hsa05217	14	55	0.446654	1
Lysine degradation	hsa00310	13	51	0.450378	1
Amphetamine addiction	hsa05031	17	68	0.457624	1
Ether lipid metabolism	hsa00565	11	43	0.458547	1
Folate biosynthesis	hsa00790	4	14	0.458931	1
Gap junction	hsa04540	22	89	0.459127	1
Long-term depression	hsa04730	15	60	0.465591	1
Phosphatidylinositol signaling system	hsa04070	20	81	0.466391	1
Adherens junction	hsa04520	18	73	0.474340	1
Salivary secretion	hsa04970	22	90	0.477514	1
Glioma	hsa05214	16	65	0.483146	1
Proximal tubule bicarbonate reclamation	hsa04964	6	23	0.486302	1
Inflammatory mediator regulation of TRP channels	hsa04750	24	99	0.487575	1
Inositol phosphate metabolism	hsa00562	15	61	0.487944	1
Alcoholism	hsa05034	43	180	0.496217	1
Calcium signaling pathway	hsa04020	43	180	0.496217	1
Pentose and glucuronate interconversions	hsa00040	9	36	0.497120	1
Prion diseases	hsa05020	9	36	0.497120	1
ErbB signaling pathway	hsa04012	21	87	0.500230	1
Pancreatic cancer	hsa05212	16	66	0.504643	1

#Term	ID	Input number	Background number	P-Value	Corrected P-Value
Tight junction	hsa04530	32	134	0.505447	1
Pancreatic secretion	hsa04972	23	96	0.509442	1
Vasopressin-regulated water reabsorption	hsa04962	11	45	0.510885	1
Pentose phosphate pathway	hsa00030	7	28	0.512447	1
Gastric acid secretion	hsa04971	18	75	0.514798	1
Porphyrin and chlorophyll metabolism	hsa00860	10	41	0.517941	1
Dopaminergic synapse	hsa04728	31	131	0.524467	1
Long-term potentiation	hsa04720	16	67	0.525933	1
Drug metabolism - other enzymes	hsa00983	11	46	0.536523	1
Epithelial cell signaling in Helicobacter pylori infection	hsa05120	16	68	0.546962	1
Cysteine and methionine metabolism	hsa00270	9	38	0.553840	1
Progesterone-mediated oocyte maturation	hsa04914	20	86	0.561313	1
beta-Alanine metabolism	hsa00410	7	30	0.575979	1
Arrhythmogenic right ventricular cardiomyopathy (ARVC)	hsa05412	17	74	0.580751	1
Pantothenate and CoA biosynthesis	hsa00770	4	17	0.588868	1
Platelet activation	hsa04611	30	131	0.589111	1
Glycosphingolipid biosynthesis - lacto and neolacto series	hsa00601	6	26	0.589881	1
Butanoate metabolism	hsa00650	6	26	0.589881	1
Alanine, aspartate and glutamate metabolism	hsa00250	8	35	0.592845	1
Ubiquitin mediated proteolysis	hsa04120	31	137	0.613047	1
Rap1 signaling pathway	hsa04015	48	211	0.613848	1
B cell receptor signaling pathway	hsa04662	16	72	0.627494	1
Other glycan degradation	hsa00511	4	18	0.627892	1
Phenylalanine metabolism	hsa00360	4	18	0.627892	1
PI3K-Akt signaling pathway	hsa04151	79	347	0.630751	1

#Term	ID	Input number	Background number	P-Value	Corrected P-Value
p53 signaling pathway	hsa04115	15	68	0.636137	1
Endocytosis	hsa04144	48	213	0.636553	1
Apoptosis	hsa04210	19	86	0.640224	1
Salmonella infection	hsa05132	19	86	0.640224	1
Histidine metabolism	hsa00340	5	23	0.640790	1
NF-kappa B signaling pathway	hsa04064	20	91	0.649803	1
Homologous recombination	hsa03440	6	28	0.652410	1
Amoebiasis	hsa05146	24	109	0.653789	1
Viral myocarditis	hsa05416	13	60	0.655339	1
Amyotrophic lateral sclerosis (ALS)	hsa05014	11	51	0.656062	1
Regulation of actin cytoskeleton	hsa04810	48	215	0.658665	1
Systemic lupus erythematosus	hsa05322	30	136	0.660302	1
Sphingolipid metabolism	hsa00600	10	47	0.668379	1
Vitamin digestion and absorption	hsa04977	5	24	0.672975	1
Morphine addiction	hsa05032	20	93	0.682557	1
Endocrine and other factor-regulated calcium reabsorption	hsa04961	10	48	0.690607	1
Type II diabetes mellitus	hsa04930	10	48	0.690607	1
Glutamatergic synapse	hsa04724	25	116	0.691256	1
Glycosphingolipid biosynthesis - ganglio series	hsa00604	3	15	0.695732	1
Retrograde endocannabinoid signaling	hsa04723	22	103	0.698013	1
Bacterial invasion of epithelial cells	hsa05100	16	76	0.700447	1
Dilated cardiomyopathy	hsa05414	19	90	0.706462	1
GABAergic synapse	hsa04727	19	90	0.706462	1
Prolactin signaling pathway	hsa04917	15	72	0.710205	1
Butirosin and neomycin biosynthesis	hsa00524	1	5	0.719255	1

#Term	ID	Input number	Background number	P-Value	Corrected P-Value
Tryptophan metabolism	hsa00380	8	40	0.720491	1
Insulin signaling pathway	hsa04910	30	141	0.724821	1
Carbohydrate digestion and absorption	hsa04973	9	45	0.726403	1
Chronic myeloid leukemia	hsa05220	15	73	0.727200	1
Mucin type O-Glycan biosynthesis	hsa00512	6	31	0.734394	1
Galactose metabolism	hsa00052	6	31	0.734394	1
Other types of O-glycan biosynthesis	hsa00514	6	31	0.734394	1
Neurotrophin signaling pathway	hsa04722	25	120	0.744831	1
Fc epsilon RI signaling pathway	hsa04664	14	70	0.754148	1
Ascorbate and aldarate metabolism	hsa00053	5	27	0.757599	1
Shigellosis	hsa05131	12	61	0.760821	1
Primary bile acid biosynthesis	hsa00120	3	17	0.764154	1
Selenocompound metabolism	hsa00450	3	17	0.764154	1
Nitrogen metabolism	hsa00910	3	17	0.764154	1
Melanoma	hsa05218	14	71	0.769884	1
Influenza A	hsa05164	37	177	0.772841	1
Riboflavin metabolism	hsa00740	2	12	0.778043	1
Phagosome	hsa04145	32	155	0.780097	1
Base excision repair	hsa03410	6	33	0.780824	1
Serotonergic synapse	hsa04726	23	114	0.783660	1
Fc gamma R-mediated phagocytosis	hsa04666	18	91	0.787021	1
Signaling pathways regulating pluripotency of stem cells	hsa04550	29	142	0.787619	1
Drug metabolism - cytochrome P450	hsa00982	13	68	0.796105	1
Allograft rejection	hsa05330	7	39	0.800789	1
Phototransduction	hsa04744	5	29	0.804148	1

#Term	ID	Input number	Background number	P-Value	Corrected P-Value
Hypertrophic cardiomyopathy (HCM)	hsa05410	16	83	0.805613	1
Metabolism of xenobiotics by cytochrome P450	hsa00980	14	74	0.812872	1
Hippo signaling pathway	hsa04390	31	154	0.815105	1
Type I diabetes mellitus	hsa04940	8	45	0.818220	1
Nicotine addiction	hsa05033	7	40	0.818904	1
Pathogenic Escherichia coli infection	hsa05130	10	55	0.819198	1
Leukocyte transendothelial migration	hsa04670	23	118	0.827352	1
Chemical carcinogenesis	hsa05204	15	80	0.827932	1
Non-small cell lung cancer	hsa05223	10	56	0.833711	1
Fat digestion and absorption	hsa04975	7	41	0.835706	1
DNA replication	hsa03030	6	36	0.838545	1
Glycosphingolipid biosynthesis - globo series	hsa00603	2	14	0.838748	1
mRNA surveillance pathway	hsa03015	17	91	0.843401	1
Rheumatoid arthritis	hsa05323	17	91	0.843401	1
Purine metabolism	hsa00230	35	176	0.844519	1
FoxO signaling pathway	hsa04068	26	134	0.845030	1
Toxoplasmosis	hsa05145	23	120	0.846636	1
Staphylococcus aureus infection	hsa05150	10	57	0.847308	1
Hedgehog signaling pathway	hsa04340	9	52	0.847806	1
Nucleotide excision repair	hsa03420	8	47	0.849033	1
Toll-like receptor signaling pathway	hsa04620	20	106	0.849408	1
Herpes simplex infection	hsa05168	37	186	0.849930	1
Taurine and hypotaurine metabolism	hsa00430	1	8	0.851262	1
Asthma	hsa05310	5	32	0.860323	1
RNA polymerase	hsa03020	5	32	0.860323	1

#Term	ID	Input number	Background number	P-Value	Corrected P-Value
Hematopoietic cell lineage	hsa04640	16	88	0.862517	1
Glycosaminoglycan biosynthesis - keratan sulfate	hsa00533	2	15	0.863079	1
Graft-versus-host disease	hsa05332	7	43	0.865569	1
Collecting duct acid secretion	hsa04966	4	27	0.868700	1
Leishmaniasis	hsa05140	13	74	0.870634	1
Retinol metabolism	hsa00830	11	64	0.871021	1
Protein digestion and absorption	hsa04974	16	89	0.872173	1
cAMP signaling pathway	hsa04024	39	200	0.881148	1
Aldosterone-regulated sodium reabsorption	hsa04960	6	39	0.883309	1
Pyrimidine metabolism	hsa00240	19	105	0.883502	1
Nicotinate and nicotinamide metabolism	hsa00760	4	28	0.884572	1
RNA transport	hsa03013	31	163	0.885584	1
SNARE interactions in vesicular transport	hsa04130	5	34	0.889723	1
Starch and sucrose metabolism	hsa00500	9	56	0.895221	1
Tuberculosis	hsa05152	34	179	0.896314	1
Renin-angiotensin system	hsa04614	2	17	0.901919	1
Sulfur relay system	hsa04122	1	10	0.902619	1
Amino sugar and nucleotide sugar metabolism	hsa00520	7	47	0.911948	1
T cell receptor signaling pathway	hsa04660	18	104	0.912419	1
Primary immunodeficiency	hsa05340	5	36	0.913627	1
Natural killer cell mediated cytotoxicity	hsa04650	24	134	0.914488	1
RNA degradation	hsa03018	12	74	0.915836	1
Cytosolic DNA-sensing pathway	hsa04623	10	64	0.919487	1
Neuroactive ligand-receptor interaction	hsa04080	53	275	0.925160	1
Vibrio cholerae infection	hsa05110	8	54	0.925362	1

#Term	ID	Input number	Background number	P-Value	Corrected P-Value
Autoimmune thyroid disease	hsa05320	8	54	0.925362	1
Malaria	hsa05144	7	49	0.929456	1
Glycosaminoglycan degradation	hsa00531	2	19	0.930264	1
Ribosome biogenesis in eukaryotes	hsa03008	13	84	0.945332	1
Synaptic vesicle cycle	hsa04721	9	63	0.948557	1
African trypanosomiasis	hsa05143	4	34	0.948895	1
TGF-beta signaling pathway	hsa04350	12	80	0.951796	1
Lysosome	hsa04142	20	122	0.952367	1
Taste transduction	hsa04742	7	53	0.955546	1
RIG-I-like receptor signaling pathway	hsa04622	10	70	0.955937	1
Ras signaling pathway	hsa04014	41	228	0.957301	1
Spliceosome	hsa03040	21	130	0.962084	1
Chagas disease (American trypanosomiasis)	hsa05142	16	104	0.962355	1
Bile secretion	hsa04976	10	72	0.964318	1
Chemokine signaling pathway	hsa04062	32	189	0.969978	1
Hepatitis C	hsa05160	21	133	0.970291	1
Glycosaminoglycan biosynthesis - heparan sulfate / heparin	hsa00534	2	24	0.971031	1
Pertussis	hsa05133	10	75	0.974223	1
mTOR signaling pathway	hsa04150	7	60	0.981199	1
Jak-STAT signaling pathway	hsa04630	24	156	0.983781	1
Intestinal immune network for IgA production	hsa04672	5	49	0.984930	1
Complement and coagulation cascades	hsa04610	8	69	0.986939	1
Measles	hsa05162	19	134	0.989454	1
Inflammatory bowel disease (IBD)	hsa05321	7	67	0.992489	1
Maturity onset diabetes of the young	hsa04950	1	25	0.995941	1

#Term	ID	Input number	Background number	P-Value	Corrected P-Value
Glycosylphosphatidylinositol(GPI)-anchor biosynthesis	hsa00563	1	25	0.995941	1
Basal transcription factors	hsa03022	3	45	0.997118	1
Regulation of autophagy	hsa04140	2	39	0.998196	1
MicroRNAs in cancer	hsa05206	45	297	0.998618	1
Cytokine-cytokine receptor interaction	hsa04060	37	265	0.999457	1
Aminoacyl-tRNA biosynthesis	hsa00970	3	66	0.999934	1
Olfactory transduction	hsa04740	12	405	1.000000	1

Supplementary Table S7. Functions of differentially expressed lncRNAs based on lncRNA-mRNA co-expression and co-location network. co-expression (in trans)

lncRNA_ID	lncRNA_Gene_ID	Status	mRNA_Gene_ID	mRNA_Gene Symbol	PCC	*P* value
LNC_000049	XLOC_009083	Novel_lncRNA	ENSG00000134755	DSC2	0.954711164	0.000224
LNC_000049	XLOC_009083	Novel_lncRNA	ENSG00000185966	LCE3E	0.963192173	0.000121
LNC_000057	XLOC_010023	Novel_lncRNA	ENSG00000140519	RHCG	0.966894691	8.85E-05
LNC_000057	XLOC_010023	Novel_lncRNA	ENSG00000063176	SPHK2	0.965867375	9.69E-05
LNC_000057	XLOC_010023	Novel_lncRNA	ENSG00000090905	TNRC6A	0.992353896	1.11E-06
LNC_000089	XLOC_019043	Novel_lncRNA	ENSG00000171711	DEFB4A	0.966450708	0.000092
LNC_000089	XLOC_019043	Novel_lncRNA	ENSG00000140519	RHCG	0.977316092	2.87E-05
LNC_000097	XLOC_021040	Novel_lncRNA	ENSG00000157985	AGAP1	0.959665983	0.000159
LNC_000097	XLOC_021040	Novel_lncRNA	ENSG00000186832	KRT16	0.983806403	1.05E-05
LNC_000097	XLOC_021040	Novel_lncRNA	ENSG00000205420	KRT6A	0.964893411	0.000105
LNC_000097	XLOC_021040	Novel_lncRNA	ENSG00000185479	KRT6B	0.984935012	8.45E-06
LNC_000097	XLOC_021040	Novel_lncRNA	ENSG00000170465	KRT6C	0.983193541	1.17E-05
LNC_000097	XLOC_021040	Novel_lncRNA	ENSG00000185966	LCE3E	0.964183444	0.000112
LNC_000098	XLOC_021043	Novel_lncRNA	ENSG00000171711	DEFB4A	0.952327975	0.000261
LNC_000098	XLOC_021043	Novel_lncRNA	ENSG00000134755	DSC2	0.968326513	7.76E-05
LNC_000099	XLOC_022153	Novel_lncRNA	ENSG00000140519	RHCG	0.995352861	2.5E-07
LNC_000102	XLOC_022585	Novel_lncRNA	ENSG00000171711	DEFB4A	0.984766445	8.74E-06
LNC_000104	XLOC_023709	Novel_lncRNA	ENSG00000072518	MARK2	0.959347568	0.000163
LNC_000262	XLOC_060045	Novel_lncRNA	ENSG00000143452	HORMAD1	0.992997346	8.54E-07
LNC_000262	XLOC_060045	Novel_lncRNA	ENSG00000150995	ITPR1	0.992039029	1.25E-06
LNC_000291	XLOC_066346	Novel_lncRNA	ENSG00000185479	KRT6B	0.984626445	8.98E-06
LNC_000291	XLOC_066346	Novel_lncRNA	ENSG00000185966	LCE3E	0.996999162	6.74E-08
LNC_000310	XLOC_070305	Novel_lncRNA	ENSG00000186832	KRT16	0.96474883	0.000107

lncRNA_ID	lncRNA_Gene_ID	Status	mRNA_Gene_ID	mRNA_Gene Symbol	PCC	*P* value
LNC_000311	XLOC_070305	Novel_lncRNA	ENSG00000157985	AGAP1	0.971898129	5.43E-05
LNC_000311	XLOC_070305	Novel_lncRNA	ENSG00000131778	CHD1L	0.950737963	0.000288
LNC_000311	XLOC_070305	Novel_lncRNA	ENSG00000186832	KRT16	0.986797601	5.7E-06
LNC_000311	XLOC_070305	Novel_lncRNA	ENSG00000205420	KRT6A	0.996406379	1.16E-07
LNC_000311	XLOC_070305	Novel_lncRNA	ENSG00000170465	KRT6C	0.993553555	6.66E-07
LNC_000329	XLOC_073645	Novel_lncRNA	ENSG00000214517	PPME1	0.970375012	6.36E-05
AC092162.1	ENSG00000230552.5	Annotated_lncRNA	ENSG00000168490	PHYHIP	0.951735811	0.000271
LINC01094	ENSG00000251442.5	Annotated_lncRNA	ENSG00000205420	KRT6A	0.95073002	0.000288
PSMD5-AS1	ENSG00000226752.7	Annotated_lncRNA	ENSG00000150995	ITPR1	0.971957562	0.000054
RP11-1112J20.2	ENSG00000259093.1	Annotated_lncRNA	ENSG00000171711	DEFB4A	0.95464201	0.000225
RP11-356I2.4	ENSG00000237499.6	Annotated_lncRNA	ENSG00000170345	FOS	0.98741927	4.93E-06
RP11-356I2.4	ENSG00000237499.6	Annotated_lncRNA	ENSG00000125740	FOSB	0.961295984	0.000141
RP11-356I2.4	ENSG00000237499.6	Annotated_lncRNA	ENSG00000168490	PHYHIP	0.974579649	4.03E-05
RP11-783K16.5	ENSG00000256940.1	Annotated_lncRNA	ENSG00000198074	AKR1B10	0.951570847	0.000274
RP11-783K16.5	ENSG00000256940.1	Annotated_lncRNA	ENSG00000134755	DSC2	0.98150861	1.56E-05
RP11-783K16.5	ENSG00000256940.1	Annotated_lncRNA	ENSG00000185966	LCE3E	0.961640025	0.000137
SNHG5	ENSG00000203875.10	Annotated_lncRNA	ENSG00000125740	FOSB	0.964646052	0.000108
XIST	ENSG00000229807.9	Annotated_lncRNA	ENSG00000115947	ORC4	0.982184894	1.39E-05
co-expression(in trans)						
Chromosome	lncRNA_Gene_ID	lncRNA_Gene_Symbol	lncRNA_Status	mRNA_Gene_Id	mRNA_Gene_Symbol	Distance
chr6	ENSG00000237499.6	RP11-356I2.4	Annotated_lncRNA	ENSG00000118503	TNFAIP3	-261
